# A Review of Anode Materials for Dual-Ion Batteries

**DOI:** 10.1007/s40820-024-01470-w

**Published:** 2024-07-24

**Authors:** Hongzheng Wu, Shenghao Luo, Hubing Wang, Li Li, Yaobing Fang, Fan Zhang, Xuenong Gao, Zhengguo Zhang, Wenhui Yuan

**Affiliations:** 1 School of Chemistry and Chemical Engineering, Guangdong Province, South China University of Technology, Guangzhou, 510641 People’s Republic of China; 2 Zhuhai Modern Industrial Innovation Research Institute of South China University of Technology, Zhuhai, 519125 Guangdong Province People’s Republic of China; 3 School of Environment and Energy, Guangdong Province, South China University of Technology, Guangzhou, 510641 People’s Republic of China

**Keywords:** Dual-ion batteries, Anode, Carbonaceous materials, Metallic materials, Organic materials, Optimization strategies

## Abstract

Distinct from "rocking-chair" lithium-ion batteries (LIBs), the unique anionic intercalation chemistry on the cathode side of dual-ion batteries (DIBs) endows them with intrinsic advantages of low cost, high voltage, and eco-friendly, which is attracting widespread attention, and is expected to achieve the next generation of large-scale energy storage applications. Although the electrochemical reactions on the anode side of DIBs are similar to that of LIBs, in fact, to match the rapid insertion kinetics of anions on the cathode side and consider the compatibility with electrolyte system which also serves as an active material, the anode materials play a very important role, and there is an urgent demand for rational structural design and performance optimization. A review and summarization of previous studies will facilitate the exploration and optimization of DIBs in the future. Here, we summarize the development process and working mechanism of DIBs and exhaustively categorize the latest research of DIBs anode materials and their applications in different battery systems. Moreover, the structural design, reaction mechanism and electrochemical performance of anode materials are briefly discussed. Finally, the fundamental challenges, potential strategies and perspectives are also put forward. It is hoped that this review could shed some light for researchers to explore more superior anode materials and advanced systems to further promote the development of DIBs.

## Highlights

- The development history and working mechanism of dual-ion batteries are reviewed, with an emphasis on the latest advancement in anode materials.
- A comprehensive and detailed summary of the synthesis strategies, structural optimization, performance characterization, and reaction principles of four types of anode materials for dual-ion batteries is presented.
- The current challenges of anode materials are highlighted, and the optimization strategies of advanced anode materials and battery systems are discussed, providing future research directions for the design of commercial dual-ion batteries.

## Graphical abstract

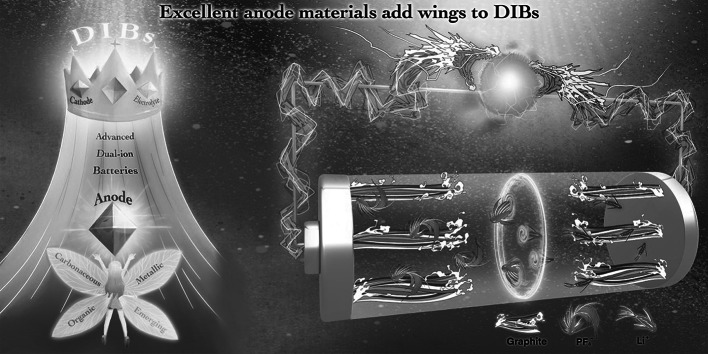

## Introduction

The development based on traditional fossil fuels is unsustainable and will result in serious pollution to the environment [1, 2]. With the increasing severity of energy and environmental issues, it is vital to develop a clean and sustainable energy supply, hence the emergence of electrochemical energy storage technology. Since the commercialization in 1991, lithium-ion batteries (LIBs) have dominated the market for electric vehicles and portable electronic devices due to their high energy density and long cycle life [3–5]. However, the operating voltage of LIBs is relatively low compared to the future demand for electrochemical energy storage, and the scarcity and uneven distribution of lithium and cobalt resources make cathode materials unsustainable and expensive, which also enhance the manufacturing and utilization costs of LIBs [6–8]. Furthermore, LIBs display a relatively high security risk. As a matter of fact, the news about the spontaneous combustion or explosion of electric vehicles using LIBs has been frequent in recent years [9, 10]. Therefore, LIBs are not suitable for large-scale energy storage applications, and the exploration and development of novel high-performance, non-polluting, and low-cost energy storage devices has become an urgent global issue, which requires the joint efforts of researchers.

**Fig. 1:**
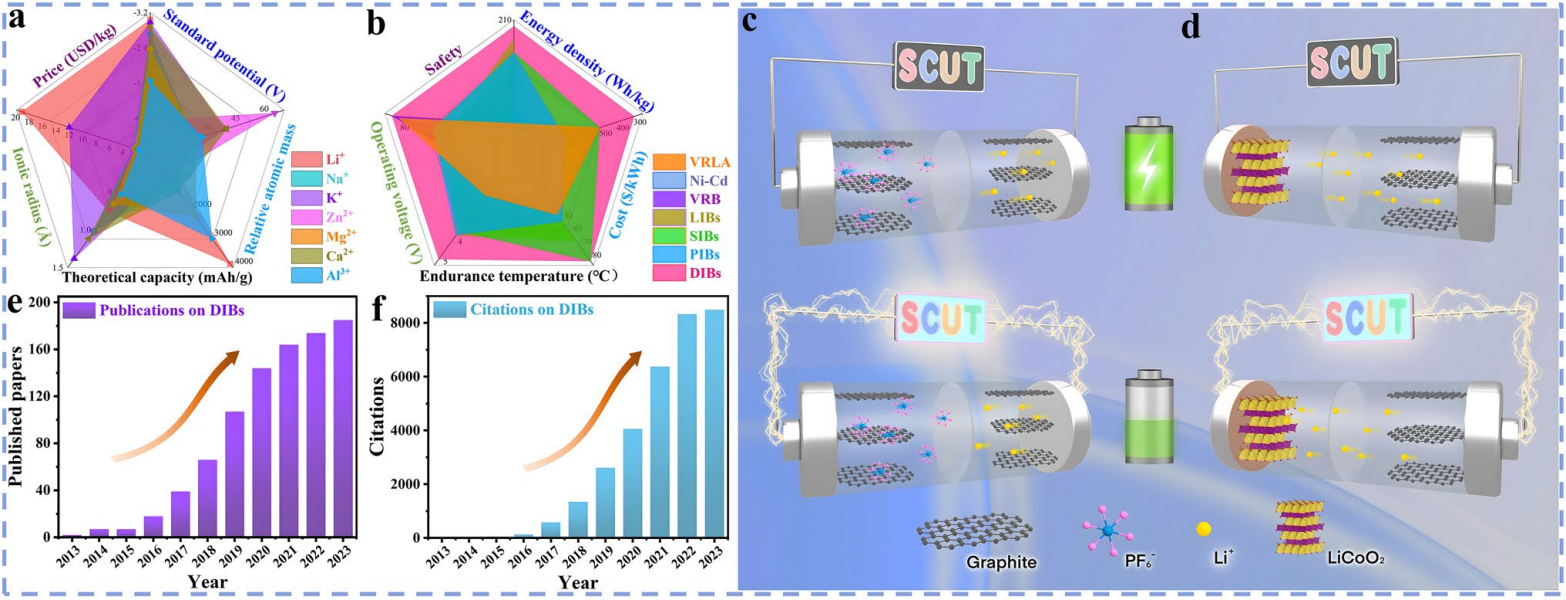
**a** Comparison of comprehensive properties of various metal elements. **b** Comparison of the overall performance of various types of batteries. **c** Schematic of the working mechanism of LIBs and **d** the operating mechanism of DIBs. **e** The number of articles on DIBs published in the past decade. **f** The citations of articles on DIBs in the last ten years

Currently, research based on the earth-abundant cations has been reported one after another [11–13]. As shown in Fig. 1a, these cations such as Na^+^ and K^+^ exhibit similar properties to Li^+^ but with higher elemental abundance in the crust (2.7 wt% Na and 2.4 wt% K, vs. 0.002 wt% Li) and lower cost [14, 15]. Therefore, a series of innovative energy storage battery systems have been subsequently developed, for instance, monovalent sodium-ion batteries (SIBs) [16, 17], potassium-ion batteries (PIBs) [18, 19]; bi-valent zinc-ion batteries (ZIBs) [20–22], magnesium-ion batteries (MIBs) [23, 24], calcium-ion batteries (CIBs) [25, 26]; tri-valent aluminum-ion batteries (AIBs) [27–29] as well as dual-ion batteries (DIBs) [30–33], etc. Some battery systems exhibit superior electrochemical performance, lower cost, and greater safety than conventional batteries (e.g., lead-acid batteries, nickel–cadmium batteries, and LIBs) as illustrated in Fig. 1b. Among them, DIBs are a battery system that balances both cations and anions, exhibiting a unique working mechanism different from that of "rocking-chair" batteries, in which the anions also act as charge carriers and participate in the electrochemical reactions. Specifically, to visualize the differences in the working mechanism between the two systems, as depicted in Fig. 1c, d, different from LIBs, during charging, active anions derived from the electrolyte move toward the cathode and intercalated into the electrode structure. Meanwhile, active cations shift to the anode and participate in the electrochemical reactions such as intercalation, alloying, conversion, and enolization reactions [34–36]. The discharging process is opposite, with active anions and cations returning to the electrolyte from the cathode and anode, respectively. The electrode reactions involved are indicated in Eqs. (1–3) (taking the classical dual-carbon electrodes and LiPF_6_ electrolyte as an example):

1 $${\text{Negative}}\;{\text{electrode}}:{\text{C}}_{{\text{A}}} + {\text{xLi}}^{ + } + {\text{xe}}^{ - } \Leftrightarrow {\text{Li}}_{{\text{x}}} {\text{C}}_{{\text{A}}}$$

2 $${\text{Positive}}\;{\text{electrode}}:{\text{C}}_{{\text{C}}} + {\text{yPF}}_{6}^{ - } \Leftrightarrow \left( {{\text{PF}}_{6} } \right)_{{\text{y}}} {\text{C}}_{{\text{C}}}$$

3 $${\text{Overall}}:{\text{C}}_{{\text{A}}} + {\text{xLi}}^{ + } + {\text{C}}_{{\text{C}}} + {\text{yPF}}_{6}^{ - } \Leftrightarrow {\text{Li}}_{{\text{x}}} {\text{C}}_{{\text{A}}} + \left( {{\text{PF}}_{6} } \right)_{{\text{y}}} {\text{C}}_{{\text{C}}}$$

where C_A_ and C_C_ are negative and positive carbon, respectively. In particular, as the single source of active ions in the DIBs system, the electrolyte acts as both an ion transport medium and an active material, which plays a key role in enhancing energy density and electrochemical energy storage of DIBs. Therefore, rational design and optimization of the electrolyte system is important to improve the electrochemical performance of the target anode materials. Besides, cost and safety are also important issues to be considered in the practical application of electrolyte design. For example, a variety of phosphate esters, phosphonates, and ionic liquids with flame-retardant properties can be employed as electrolyte and demonstrate good compatibility with the electrode materials of DIBs, which significantly improves the safety and electrochemical performance of DIBs [37–39]. In addition, graphite, which is completely free of metal elements, can be used as the cathode active material for DIBs, thus remarkably reducing the cost compared to the commonly used expensive cathode materials for LIBs (LiCoO₂, LiMn₂O₄, LiFePO₄, and Ni-Co-Mn ternary materials, etc.) [40, 41]. Further, since the high positive potential (~ 5.0 V vs. Li/Li^+^) of anion insertion into graphite results in higher working voltage and energy density of DIBs compared to commercial LIBs [42, 43]. The special reaction mechanism of DIBs requires that the cathode materials should possess fast ionic insertion kinetics, excellent structural stability, and abundant active sites to rapidly and repeatedly store as many large-sized reactive anions as possible and is expected to solve the capacity mismatch of the cathode and anode. The electrolyte should be stable at high potentials without oxidative decomposition and co-insertion of solvent molecules, with no side reactions and corrosion of the collector during cycling, and good compatibility with the electrodes. To sum up, owing to the advantages of low cost, environmentally friendly, high operating voltage, DIBs have attracted extensive attention in recent years and gradually evolved into the state-of-the-art battery energy storage system, which is highly promising to become a strong candidate for the next generation of efficient large-scale energy storage batteries. The continued increase in research interest can also be evidenced in Fig. 1e, f, where the number of publications and citations related to DIBs has increased rapidly over the last decade [44–47]. However, although some review articles about DIBs have been published sequentially, most of them basically focus on the preparation and performance studies of cathode materials, the exploration and optimization of electrolyte systems or a general and brief overview of the research progress of DIBs. Most important of all, to the best of our knowledge, up to now there is no dedicated review to summarize the synthesis, performance research, structure characterization and mechanism derivation of DIBs anode materials in detail. Actually, for the anode, it should feature high theoretical capacity and low operating potential to essentially achieve the high specific discharge capacity, remarkable working voltage and energy density of DIBs. More importantly, the anode materials should have efficient cation reduction reaction and rapid transport kinetics to match the fast insertion kinetics of anions on the cathode side and achieve superior rate capability; high binding capability to avoid spontaneous release of active ions, thereby reducing self-discharge rate; favorable compatibility with the electrolyte to form a solid electrolyte interphase (SEI) with good mechanical strength and ion transport, and to realize the stabilization and concentration requirements of the electrolyte under high voltage. Therefore, the anode materials equally play an essential role, and a rational design and performance optimization of the anode material is urgently needed. It is necessary to go for a systematic review of the latest research progress of DIBs anode materials, including synthesis, modification, performance testing, structural characterization, and mechanism exploration. Finally, some outstanding challenges faced by the current DIBs anode materials are discussed and corresponding improvement strategies and perspectives are proposed, aiming to provide researchers with a clearer understanding and better promote the realization of advanced dual-ion batteries with low cost and high performance.

## Development History of DIBs

**Fig. 2:**
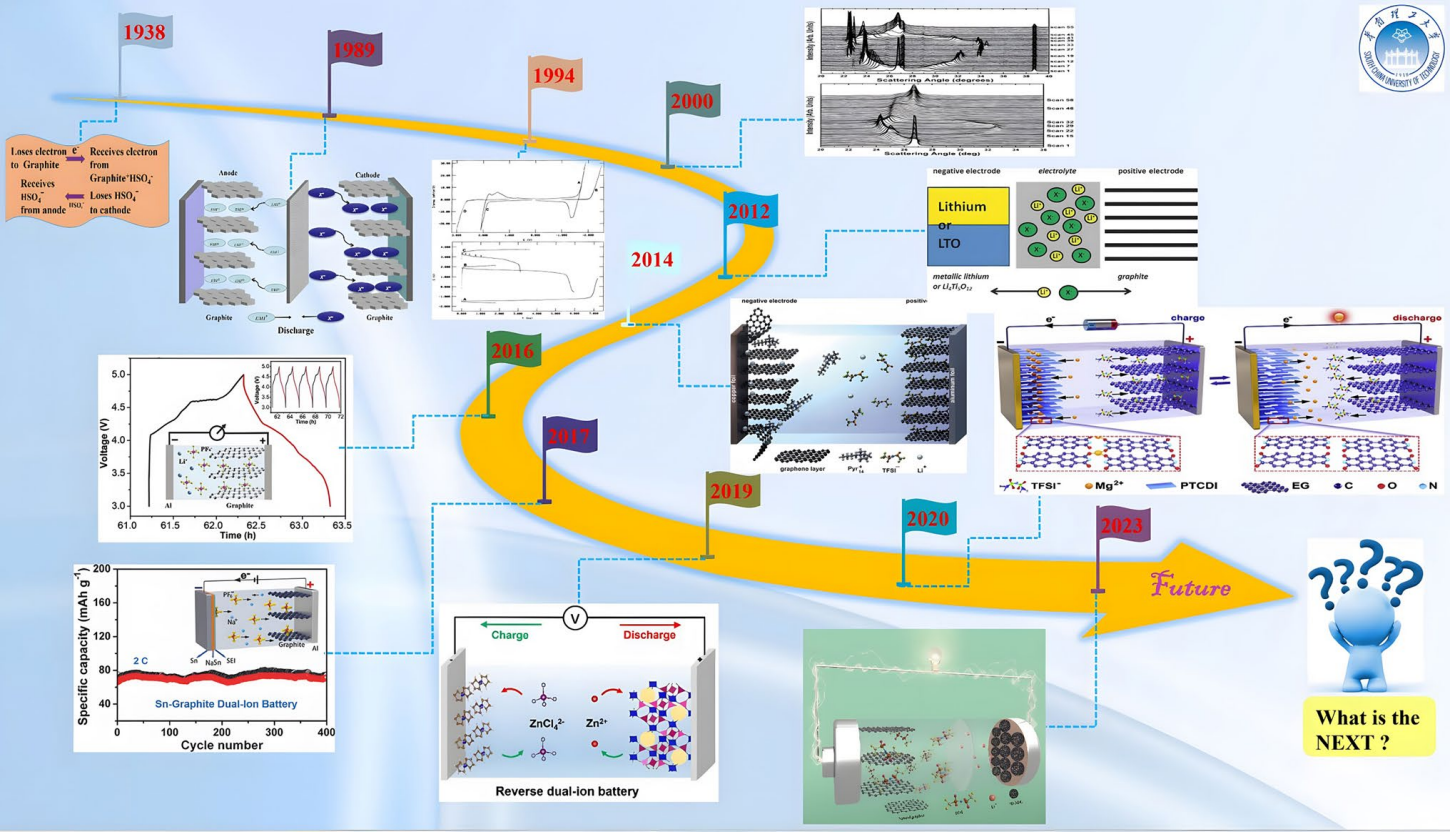
Development history of dual-ion batteries. A type of graphite-based battery, reproduced with permission [48]. Copyright 1938, Wiley. The concept of "double graphite cell" or "double intercalation", reproduced with permission [49]. Copyright 1989, US Patent. The room-temperature ionic liquid-based DGBs, reproduced with permission [50]. Copyright 1994, The Electrochemical Society. The demonstration that the insertion of active ions is a staged process, reproduced with permission [51]. Copyright 2000, The Electrochemical Society. Formally introduced the concept of a "dual-ion battery", reproduced with permission [53]. Copyright 2012, The Electrochemical Society. The 5.2-V class dual-graphite battery, reproduced with permission [54]. Copyright 2014, The Royal Society of Chemistry. The Li-DIBs based on Al anode, reproduced with permission [56]. Copyright 2016, Wiley. The Na-DIBs and K-DIBs based on Sn anode, reproduced with permission [57, 58]. Copyright 2017, Wiley. The first reverse dual-ion battery, reproduced with permission [60]. Copyright 2019, American Chemical Society. The Mg-DIBs, reproduced with permission [61]. Copyright 2020, American Chemical Elsevier. The 6-V class DIBs, reproduced with permission [62]. Copyright 2021, Wiley. The dual-carbon battery with a capacity over 350 mAh g^−1^, reproduced with permission [63]. Copyright 2023, Wiley

In order to better understand the dual-ion battery, a brief review of its development history is described in Fig. 2. As an innovative battery energy storage system, DIBs have been developed in leaps and bounds in recent years, but the related concept of anion insertion was introduced as far back as 1938, when Rüdorff and Hofmann confirmed the reversible insertion of HSO_4_^−^ into graphite and prepared a type of graphite-based battery [48]. Later in 1989, McCullough et al. introduced the concept of "double graphite cell" or "double intercalation" to explain the reaction process of the cell [49]. Then in 1994, Carlin et al. reported the room-temperature ionic liquid-based dual-graphite batteries (DGBs), demonstrating that the large-size 1-ethyl-3-methylimidazolium (EMI^+^) cation could be reversibly intercalated in the graphite anode [50]. In 2000, Seel et al. demonstrated that the insertion of active ions into electrodes is a stage process through in situ XRD characterization [51]. Ishihara et al. explored the effect of graphite crystallinity on the intercalation of reactive ions in 2007, proving that an increase in crystallinity favors the intercalation of reactive ions [52]. It was not until 2012 that the concept of the "dual-ion battery" was formally put forward by Placke et al. and has been applied ever since [53]. In 2014, Read et al. reported the realization of a 5.2 V high-voltage dual-graphite battery based on fluorinated electrolytes and additives [54]. In 2015, Dai et al. reported the Al-DIBs based on Al metal anode, while Tang et al. discovered the Li-DIBs based on Al anode in 2016 [55, 56]. To further reduce the cost, in 2017, Sheng et al. reported the sodium-based dual-ion batteries (Na-DIBs) based on Sn anode, and Ji et al. debuted the potassium-based dual-ion batteries (K-DIBs) with Sn and Pb foil as anode, respectively [57, 58]. In 2018, Wang et al. reported the calcium-based dual-ion batteries (Ca-DIBs) that could cycle stably at room temperature [59]. The concept of a reverse dual-ion battery (RDIB) based on ferrocene nanocomposite anode was first proposed by Wu et al. in 2019 [60]. In 2020, Lei et al. debuted the magnesium-based dual-ion batteries (Mg-DIBs) based on 3,4,9,10-perylenetetracarboxylic diimide (PTCDI) organic anode [61]. While Tong et al. reported a type of DIBs based on a high-concentration sulfone-based electrolyte in 2021, which could further increase the oxidation potential to 6.0 V [62]. More recently, Yuan et al. proposed a novel class of DIBs based on an innovative concentrated organic solvent-ionic liquid hybrid electrolyte in 2023, which can increase the specific discharge capacity (SDC) beyond 350 mAh g^−1^ [63]. While Wang et al. increased the median discharge voltage of DIBs to 4.67 V through the synergistic solvation effect of anions, which further advance the practical application of DIBs [64].

**Fig. 3:**
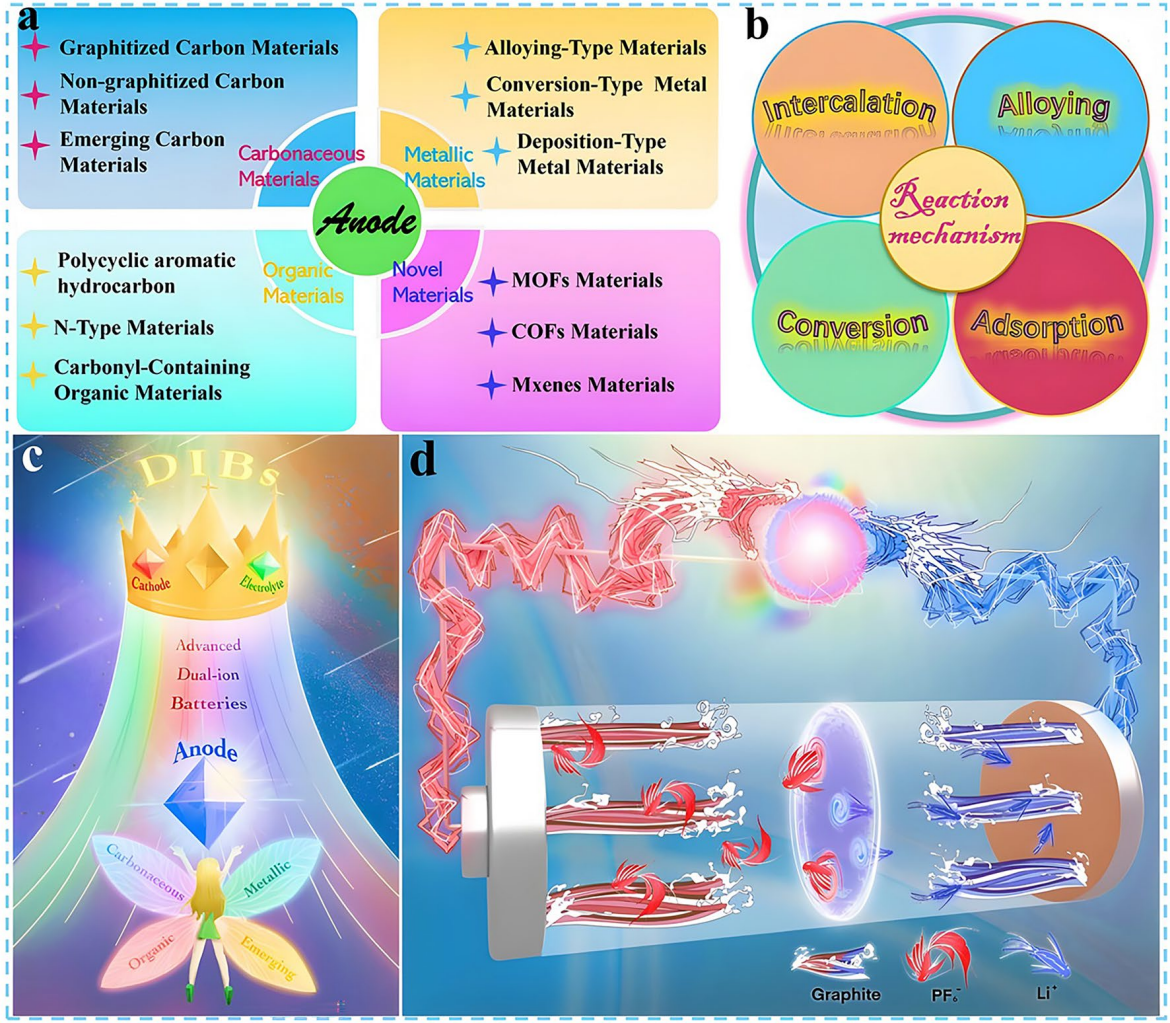
**a, c** Four types of anode materials applied in DIBs systems. **b, d** Corresponding reaction mechanisms

Nevertheless, compared with state-of-the-art of LIBs, the current research on DIBs is still in the primary stage with the technology still immature. Although the electrochemical reactions on the anode side of DIBs are similar to that of LIBs, in fact, to match the rapid insertion kinetics of anions on the cathode side and consider the compatibility with electrolyte system which also serves as an active material, the anode materials play an extremely crucial role, and there is an urgent demand for rational structural design and performance optimization. Therefore, in the following sections, the research progress of DIBs anode materials will be discussed in detail, including the synthesis strategy, structural optimization, performance characterization and reaction mechanism of anode materials. Moreover, the current challenges are summarized and the potential solutions, as well as the future development direction of anode materials for DIBs are put forward. Meanwhile, it is also expected that this review can draw the attention of more researchers to DIBs, attract more people to join the research field of DIBs, and promote its practical application faster. As displayed in Fig. 3a, c, based on the unique physicochemical properties of the material itself, the anode materials currently applied in DIBs can be broadly divided into four categories, which are carbonaceous materials, metallic materials, organic materials, and emerging materials in recent years such as MOFs, COFs, and MXenes materials. Accordingly, there are four main working mechanisms involved in these materials, namely, intercalation reaction, alloying reaction, conversion reaction, and adsorption reaction (Fig. 3b, d). In the following, these four types of materials will be discussed separately, aiming to provide readers with a clearer understanding of DIBs, especially the anode materials, and to promote the development of advanced DIBs.

## Carbonaceous Materials

### Graphitized Carbon Materials

**Fig. 4:**
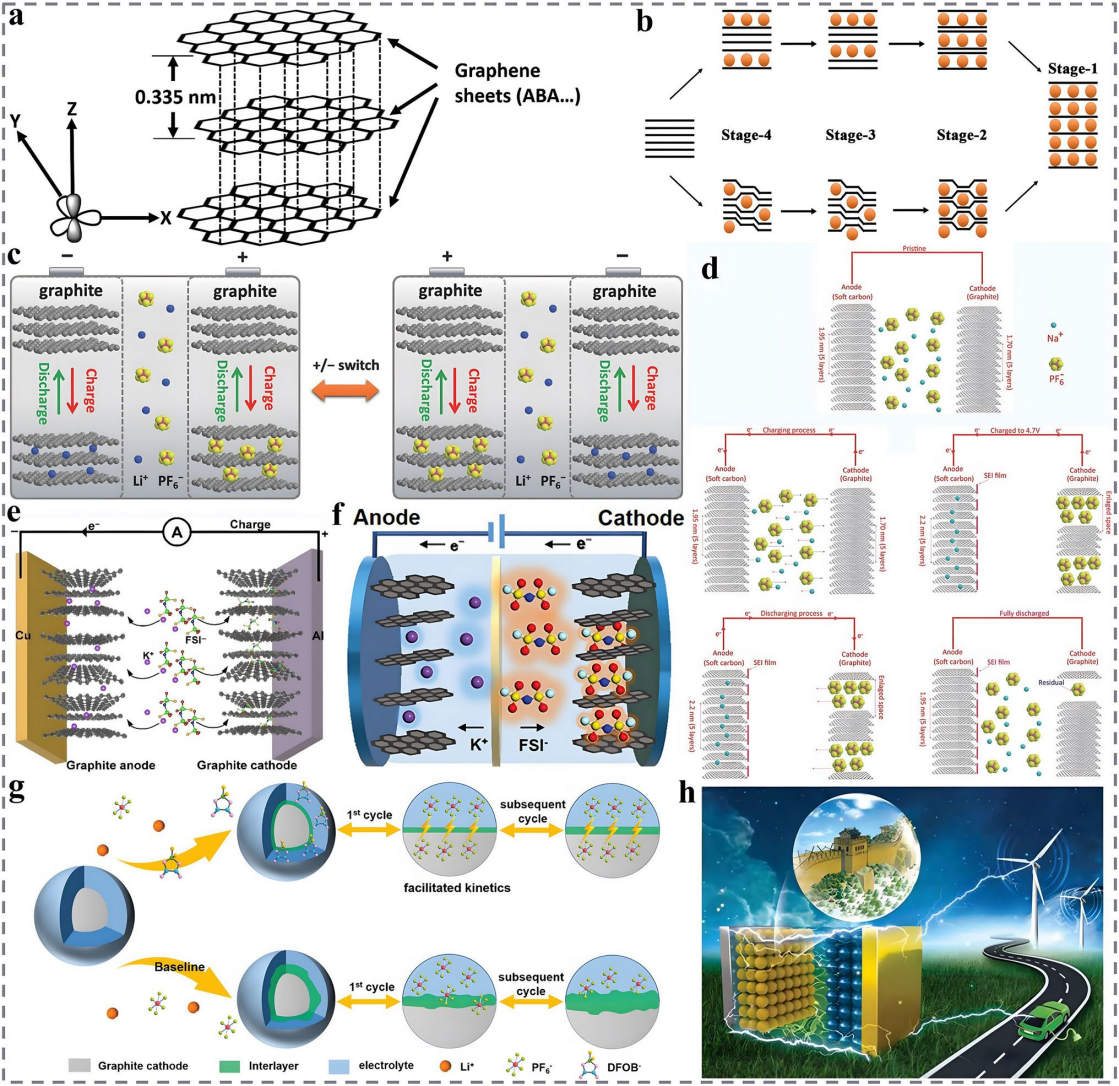
Schematic representation of the reaction mechanism and the corresponding modification strategies for graphitized materials. **a** Crystal structure of graphite, reproduced with permission [70]. Copyright 2011, Elsevier. **b** Schematic illustration of different stages of intercalated graphite, reproduced with permission [74]. Copyright 2014, Royal Society of Chemistry. **c** Working principle of the polarity-switchable DGDIBs, reproduced with permission [75]. Copyright 2018, Wiley. **d** Schematic diagram of NDIBs at the states of pristine, charging process, fully charged, discharging process, and fully discharged, reproduced with permission [77]. Copyright 2017, Wiley. **e** Schematic diagram of the working mechanism of KDGBs, reproduced with permission [78]. Copyright 2023, Springer Nature. **f** Battery configuration and working mechanism of the K-ion dual-graphite battery, reproduced with permission [79]. Copyright 2020, Wiley. **g** Illustration of the CEI evolution during cycling in electrolyte with and without LiDFOB additive, reproduced with permission [84]. Copyright 2021, Wiley. **h** Schematic diagram of the chemical coating and in situ polymerization of CEI on LTO-modified MCMB, reproduced with permission [85]. Copyright 2019, Wiley

As mentioned above, the development of DIBs was initially facilitated by the study of graphite intercalation compounds (GICs). Meanwhile, the inspiring thing about graphite is that it exhibits redox duality, which can not only accommodate anions (PF_6_^−^, TFSI^−^, ClO_4_^−^, BF_4_^−^, TfO^−^, etc.), but also store cations (Li^+^, Na^+^, K^+^, Zn^2+^, Ca^2+^, Mg^2+^, Al^3+^, etc.) [65–69]. Therefore, graphite-based materials not only have been one of the most successful materials applied in the DIBs cathode system, but also is the dominant anion host material of DIBs at present. It is well known that graphite is a three-dimensional (3D) material consisting of two-dimensional (2D) graphene sheets stacked together in ABAB order through π–π interactions and van der Waals forces stacking with a layer spacing of 0.335 nm (Fig. 4a) [70, 71]. This unique layered structure features excellent mechanical strength (130 GPa) and high Young's modulus (1 TPa), allowing stable and reversible intercalation/de-intercalation of charge carriers [72, 73], wherein the reaction formula of the cations insertion into the graphite layered structure can be expressed as follows:

4 $${\text{xC}} + {\text{M}}^{{{\text{n}} + }} + {\text{n}}^{{{\text{e}} - }} \Leftrightarrow {\text{M}}_{{\text{n}}} {\text{C}}_{{\text{x}}}$$

Interestingly, when the intercalation reaction occurs, the reactive ions are not intercalated into the graphite layer structure at the same time, but rather, the process is carried out step by step in a certain order. This process is defined as "staging", as shown in Fig. 4b, and the stage number represents the number of graphene layers between adjacent intercalated active ions [74]. However, graphite-based anode materials face a relatively slow desolvation process, leading to unsatisfactory cation reaction kinetics on the anode side, which is difficult to match the fast anion insertion kinetics on the cathode side. Therefore, rational design of the electrolyte system or relevant modification of the material is needed to avoid the desolvation process and improve the cation reduction reaction rate and transport kinetics on the anode side. Specifically, Feng et al. ingeniously designed a polarity-switchable DIBs presented in Fig. 4c, in which graphite was used as both anode and cathode, and the electrolyte was 2 M LiPF_6_-3 wt% VC [75]. During the charging process, anions and cations shift toward the cathode and the anode, respectively, when the polarity is switched, the roles of the cathode and the anode are interchanged, the reversible intercalation/de-intercalation of active ions can still be achieved. Impressively, the electrodes are continuously activated during polarity switching, while the functional additive VC facilitates the formation of a more stable SEI and reduces the anion insertion potential. Thus, it effectively inhibits the further decomposition of the electrolyte and leading to faster reaction kinetics and superior electrochemical performance relative to the initial battery system, with a median voltage as high as 4.5 V and a high energy density (ED) of 227 Wh kg^−1^ (based on the total mass of anode and cathode). Xu et al. introduced a fluorinated solvent and additive (1.7 M LiPF_6_ in FEC: EMC = 4:6, w:w + 5 mM HFIP) into a DIB system based on a mesophase carbon microspheres (MCMB) anode [76]. This fluorinated electrolyte can in situ generate a protective SEI on the MCMB that is rich in inorganic components such as Li-F and is mechanically strong, which significantly enhances the Li^+^ transport kinetics and support the efficient insertion of Li^+^ into the anode at potentials as low as 0.2 V, thus enabling the DIBs to perform redox reactions at 5.2 V.

Besides, several DIBs systems based on sodium and potassium salt electrolytes have been reported. For example, Lu et al. developed a high-performance sodium-based DIB (SDIB) with soft carbon anode and 1 M NaPF_6_-EC-DMC electrolyte (Fig. 4d), which delivered a high SDC of 103 mAh g^−1^ and a high discharge platform of 3.58 V at 200 mA g^−1^ [77]. Even at a high current density of 1000 mA g^−1^, the SDIB can be stably cycled for 800 cycles with a capacity retention (CR) of 81.2%. Li et al. reported a potassium-based DIB (KDIB) based on graphite anode and KFSI-TEP electrolyte with a salt-to-solvent molar ratio of 1:1.3 (Fig. 4e), which exhibited a high operating voltage of 4.3 V and favorable stability with a CR of 98% after 350 cycles at 1 A g^−1^ [78]. In particular, the electrolyte employed is highly non-flammable, which in turn significantly improves the safety of DIBs. To further increase the operating voltage and energy density of KDIBs, a kind of DIBs based on high-concentration potassium-based electrolyte (5.2 m KFSI-TMS) and expanded graphite (EG) cathode has been developed. This high-concentration electrolyte displays an oxidation potential of up to 6.0 V, which promotes the reaction kinetics of active ions and notably improves the intercalation reversibility and capacity of K^+^ on the graphite anode side, and the employed TMS solvent is non-flammable, thereby boosting the cycling performance, energy density, and safety of DIBs [79]. The constructed proof-of-concept KDIBs exhibit an SDC of 83.4 mAh g^−1^ at 100 mA g^−1^ and a CR of 81% after the 400 cycles at 300 mA g^−1^ (Fig. 4f). The ED is up to 130 Wh kg^−1^ (based on the total mass of cathode, anode, and electrolyte), which is the highest value reported at that time. Moreover, dual-graphite batteries based on metal-free electrolyte systems such as pure ionic liquids (ILs) have also been developed. Yuan et al., respectively, investigate the DIBs based on PP_14_TFSI and Pyr_14_TFSI ionic liquid electrolytes with composite graphite (CG) anode, the results indicate that the PP_14_TFSI electrolyte demonstrates a wider electrochemical window, better compatibility with the anode, and the ability to form thinner and stable SEI. The corresponding DIBs based on PP_14_TFSI pure ionic liquid electrolyte possess a median discharge voltage up to 4.4 V and excellent cyclic performance, which can be stably cycled for 600 cycles without capacity degradation [80, 81]. Additionally, a lower self-discharge rate is presented due to the higher binding capability between PP_14_^+^ and the anode after intercalation compared to Pyr_14_^+^, resulting in a more difficult spontaneous detachment of PP_14_^+^.

However, although exhibiting decent cation storage performance, graphitized anode materials are limited by some shortcomings such as severe structural exfoliation and continuous decomposition of the electrolyte during the repeated intercalation/de-intercalation process at high potential, resulting in performance degradation of anode, reduction of the cycle life and Coulombic efficiency, which undoubtedly hinders the further development of DIBs [82, 83]. Accordingly, researchers have adopted appropriate modification strategies to address these drawbacks. The introduction of suitable electrolytes or functional electrolyte additives can form a SEI layer with better performance, which can play a good passivation effect on the surface of the anode, promote the compatibility between the anode and the electrolyte, inhibit the further decomposition of the electrolyte and boost the kinetics of the active ions transport [37, 38, 43, 53, 54]. Specifically, as shown in Fig. 4g, a robust artificial electrode–electrolyte interfacial layer is fabricated on the surface of graphite electrode (MTI SAG-R) upon the introduction of 5 wt% lithium difluoro(oxalato)borate (LiDFOB) film-forming additive, which in turn notably improves the electrochemical performance of the dual-graphite battery [84]. The DIBs exhibit an ED of 179.8 Wh kg^−1^ (based on the total mass of cathode and anode) and a high SDC of 97.6 mAh g^−1^ at 10C (1C = 100 mA g^−1^). After 6500 cycles, the DIBs still show a CR of 92.4% with Coulomb efficiency (CE) remains at 99.4%. Functional modulation of morphology and structure is also a commonly employed modification strategy for DIBs. By adopting Li_4_Ti_5_O_12_ (LTO) with functional activity to modify the MCMB anode and pre-lithium treatment, as shown in Fig. 4h, it can not only maintain the structural integrity of electrode to avoid the collapse of graphite layer structure, but also improve the thermodynamic behavior of active ions and inhibit the further decomposition of electrolyte [85]. The corresponding dual-ion full battery exhibits a high ED of about 200 Wh kg^−1^ (based on the total mass of cathode and anode) with a CR of 93.5% after 1000 cycles at 5C. Additionally, heteroatom doping and layer spacing modulation strategies are also attractive for improving the performance of DIBs. Therefore, a type of modified graphite anode (N-LIDG-800) with large layer spacing and nitrogen atom doping is synthesized for DIBs by a one-step catalytic pyrolysis method, which possesses a remarkably higher layer spacing than that of pristine graphite (0.51 vs 0.335 nm) and provides more storage space for reactive ions [86]. Furthermore, the defects induced by nitrogen doping not only strengthen the adsorption of reactive ions and reaction kinetics, but also supply additional active sites. This synergistic effect enables the DIBs to achieve an ultra-high SDC of 240 mAh g^−1^ at 1C and be able to cycle stably for 2400 cycles.

### Non-Graphitized Carbon Materials

Compared with graphitized carbon, non-graphitized carbon materials exhibit batter electrochemical performance, such as:

1. Relatively larger layer spacing, generally greater than 0.38 nm [87].
2. Higher theoretical capacity, usually greater than 500 mAh g^−1^ [88].
3. Better rate capability and stability, and its special amorphous structure not only facilitates the storage and transport of reactive ions, but also exerts better buffering ability against the volume expansion caused by the insertion of reactive ions [89, 90].
4. Exhibit inherent superiority for the storage of Na^+^ and K^+^ [91, 92].
5. Potential cost advantage, which can be synthesized from a variety of abundant precursors with simple preparation process [93, 94].

**Fig. 5:**
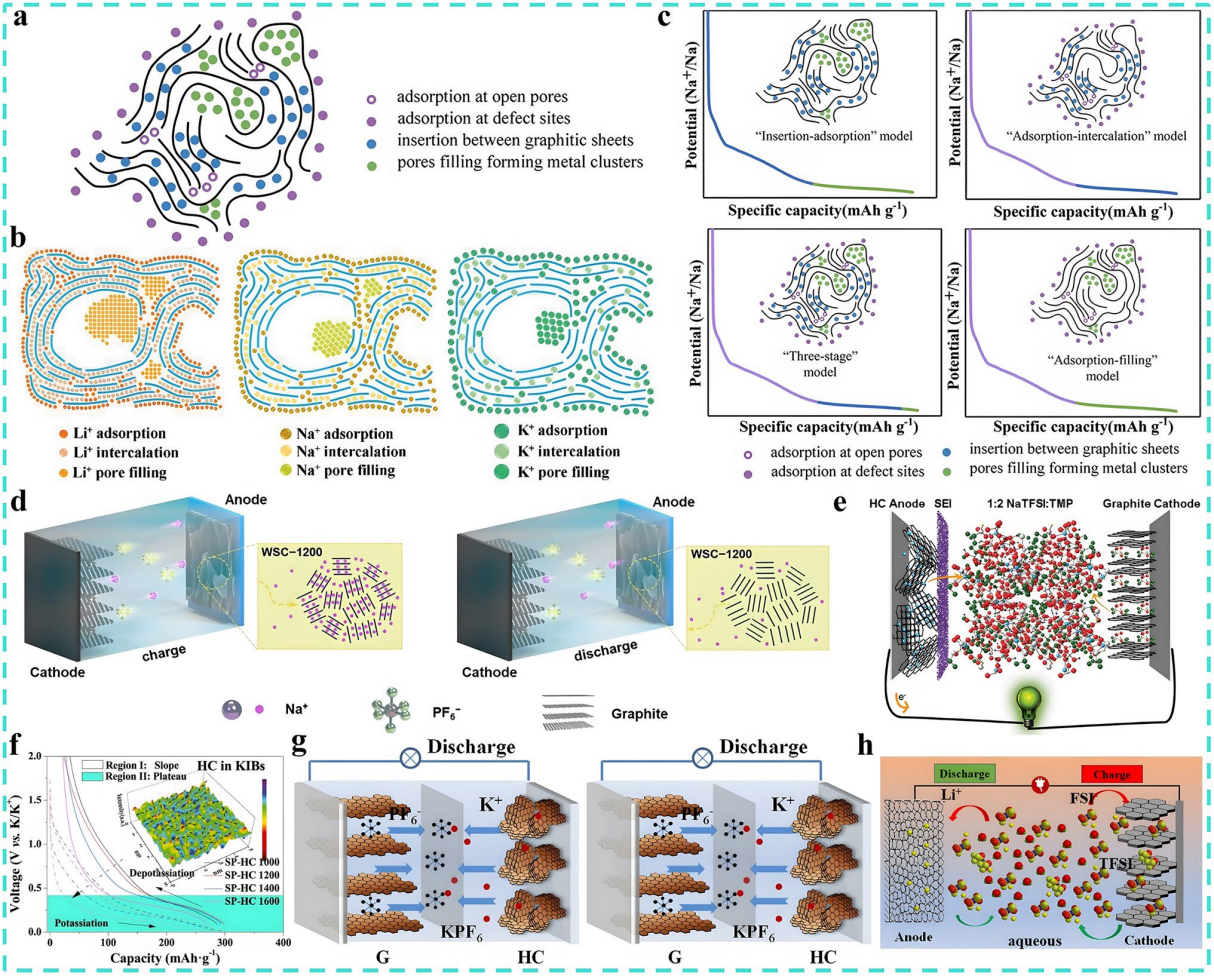
Storage mechanism of non-graphitized carbon anode materials and their application in DIBs. **a** Microstructure of hard carbon and the major active sites with the ability to store reactive ions, reproduced with permission [98]. Copyright 2022, Wiley. **b** Potential storage mechanisms for alkali metal ions in non-graphitized carbon, reproduced with permission [89]. Copyright 2021, Wiley. **c** Four types of storage mechanisms for hard carbon, reproduced with permission [98]. Copyright 2022, Wiley. **d** Schematic illustration of the WSC–1200/K SDIBs, reproduced with permission [105]. Copyright 2023, Elsevier. **e** Schematic illustration of the Na^+^-DCB using NaTFSI-TMP electrolyte, reproduced with permission [106]. Copyright 2018, Wiley. **f** Percentage of pseudo-graphite regions at different temperatures and **g** the schematic illustration of the AK-DIB with SP-HC-1200 anode, reproduced with permission [107]. Copyright 2019, Elsevier. **h** Schematic image for aqueous DIB using AC anode and LiFSI-LiTFSI bisalt electrolyte, reproduced with permission [113]. Copyright 2021, Elsevier

Although the structure of non-graphitized carbon materials is complex and diverse, there are abundant active sites for the storage of active ions. As illustrated in Fig. 5a, these active sites can be broadly divided into the following categories [95–97]:

1. Adsorption on open pore surfaces, influenced by the specific surface area of materials.
2. Adsorption of reactive ions at defect sites, including edges, heterogeneous elements, and vacancies, etc., influenced by precursor material and degree of defects.
3. Insertion of reactive ions in graphene layers, influenced by the occupancy of pseudo-graphite nanodomains.
4. Pore filling for the formation of quasi-metallic clusters, influenced by the number and size of pores.

Especially, Fig. 5b summarizes the potential storage mechanisms for alkali metal ions in non-graphitized carbon [89]. Among them, hard carbon (HC), as a typical non-graphitized carbon anode material with decent electrochemical performance, has received more and more attention and is widely applied in Li/Na/K-ion batteries [98, 99]. In recent years, HC materials have also been gradually implemented in DIBs with promising outcomes, even delivering better performance than graphite [100–102]. HC features a house of cards-like structure, consisting of curved and irregularly distributed graphene nanosheets with an amorphous structure, which is difficult to graphitize even at temperatures above 3000 °C [103, 104]. The exploration of the energy storage mechanism is essential to understand the influence of the anode materials' structure on their electrochemical performance and to guide the pre-design and synthesis of materials. Based on the theoretical calculations and experimental investigations, various mechanism models have been proposed. Although controversially, the energy storage mechanisms of HC can be roughly classified into four types, including "intercalation-adsorption", "adsorption-intercalation", "stage-to‐stage" and "adsorption-pore filling" models, as presented in Fig. 5c [98]. A number of researchers have already expanded their research direction to the application of HC in DIBs, e.g., Huang et al. prepared the biomass walnut shell-derived hard carbon (WSC) via a two-step pyrolysis method and attempted to improve the Na^+^ storage performance by modulating the area ratio of the capacitance-controlled disordered region and the diffusion-controlled pseudo-graphitic region (DR/PGR) [105]. As a result in Fig. 5d, the DIBs based on a WSC anode with an optimal DR/PGR ratio and 1 M NaPF_6_-EC/DMC (1:1, v/v) electrolyte exhibited an ultra-high SDC of 245.6 mAh g^−1^ with an ED of 172.06 Wh kg^−1^ (based on the total mass of cathode and anode). The battery system also shows a long cycle life of 30,000 cycles at 5 A g^−1^ with a low decay rate of 0.049% per cycle. To further improve safety, a non-flammable trimethyl phosphate-based concentrated electrolyte system (NaTFSI-TMP) is developed for SDIBs [106]. The electrolyte is also capable of forming a stable SEI on HC anode, which inhibits the decomposition of electrolyte and displays a decent rate capability (Fig. 5e). In this case, the Na-DIB can deliver an operating voltage of 4.0 V with no degradation after 750 cycles. As mentioned previously, the pseudo-graphite structure in HC is favorable to bring higher capacity and improved rate performance. To this end, Sichuan pepper was used as a precursor, and the HC synthesized at 1200 °C (SP-HC) was discovered to possess abundant pseudo-graphite regions through the exploration and optimization of pyrolysis temperature in Fig. 5f [107]. The K^+^ storage mechanism is investigated via combining in situ XRD characterization and kinetic analysis, and the results demonstrate that the adsorption-insertion-filling synergistic effect of SP-HC brings good storage performance (Fig. 5g). When employed in the DIBs system based on 0.8 M KPF_6_-EC-DEC electrolyte, the full battery exhibits an SDC of 67 mAh g^−1^ with a CR of 90% after 300 cycles. To promote the K^+^ storage capability of HC further, a kind of hard carbon with porous nanoparticles (NOCNBs) derived from lobster shells containing abundant active sites was synthesized by a self-template-assisted pyrolysis strategy, which demonstrates satisfactory K^+^ storage performance [108]. Density-functional theory (DFT) indicates that NOCNBs present a hierarchical multi-scale structure of microporous/mesoporous/macropore with a large layer spacing of 0.4 nm, which greatly enhances the adsorption and diffusion of K^+^, thus facilitating the capacitive-adsorptive storage. As an anode for KDIBs, the system exhibits a median voltage of 4.05 V and a SDC of 89 mAh g^−1^ with a CR of 95% after 2000 cycles.

Activated carbon (AC), as a commonly used adsorbent, is also employed in energy storage due to its high specific surface area (> 2000 m^2^ g^−1^) and good conductivity (~ 60 S m^−1^) [109–112]. Ishihara et al. reported an aqueous DCBs system based on the KS6 graphite cathode and AC anode, specifically, a novel LiFSI/LiTFSI (9:1, molar ratio) water-in-bisalt aqueous electrolyte was designed, and the effect of the variation in solvation structure was additionally investigated in detail [113]. The electronic interactions between the two supporting salts boost the oxidation potential of this aqueous electrolyte to 5.0 V, which widens the electrochemical window of DCBs to 3.1 V (Fig. 5h). The corresponding full battery delivers a SDC of 72 mAh g^−1^ and a high ED of 214 Wh kg^−1^ (based on the mass of anode) with no capacity degradation at 500 mA g^−1^ for 100 cycles. However, the cost of this water-in-salt aqueous electrolyte is too expensive. To realize the cost advantage, a class of peanut-skin-derived ACs were prepared by the KOH activation method to serve as both cathode and anode of DIB, and the cheap 1 M NaClO_4_-EC-DEC was used as the electrolyte [114]. The constructed DCBs exhibit an ED of 112 Wh kg^−1^ (based on the total mass of cathode and anode) and display stable cycling performance with a CR of 85% after 3000 cycles. While Wang et al. synthesized an AC anode with a hollow sphere structure through the SiO_2_ templating method, and designed the PDIBs based on 1 M-KPF_6_-EC-DEC-PC electrolyte, which exhibited a median voltage of up to 4.3 V and a SDC of 89.8 mAh g^−1^ at 100 mA g^−1^ with a high CR of 96.6% after 200 cycles [115]. Apart from alkali metal ions, AC can also be utilized to store divalent metal ions and non-metal cations. For instance, Placke et al. constructed a type of DCBs based on AC anode, graphite cathode, and magnesium-based ionic liquid electrolyte of 0.3 M Mg(TFSI)_2_-Pyr_14_TFSI, which exhibited a high SDC of 87 mAh g^−1^ with a CE of 98% in the voltage window of 5.2 V and can be stably cycled for 50 cycles without degradation [116]. Zheng et al. proposed the DCBs based on EMImPF_6_ pure ionic liquid electrolyte and AC anode, the full batteries exhibited an ED of 43 Wh kg^−1^ (based on the total mass of cathode and anode) with a CR of 83% after 50 cycles [117]. Other than that, some non-graphitized amorphous carbon materials are also highly promising anode materials for DIBs. As Lu et al. reported a 4.5 V-grade lithium-based full DCBs that amorphous carbon nanospheres were both as cathode and anode, which can achieve a high ED of 206.7 Wh kg^−1^ (based on the total mass of cathode and anode) and be stably cycled for 10,000 cycles with a capacity degradation rate as low as 0.0013% per cycle [118]. Hou et al. synthesized an in situ phosphorus-doped hollow carbon nanorods material (P-HCNs) with a high P doping of 7.5 at.% through the hard template method [119]. When employed in a sodium-based DIB, the full batteries supply an initial Coulombic efficiency (ICE) of up to 73%, an ED of 138 Wh kg^−1^ (based on the total mass of cathode and anode) and a high SDC of 158 mAh g^−1^ at 500 mA g^−1^ with a CR of 78.5% after 1200 cycles. The impressive reversible capacity is attributed to the strong Na^+^ adsorption capability of P=O and P–C bonds as determined through the first-principle calculations. Tang et al. designed a 3D nanoporous locally ordered composite carbon nanowire material (CCNW) for stable K^+^ storage [120]. The proof-of-concept potassium-based DIBs exhibit good stability and decent capacity, which deliver a high SDC of 134.4 mAh g^−1^ at 100 mA g^−1^ and are stable to cycle for more than 1000 cycles. In particular, no binder is required for the preparation of CCNW anode, which further improves the energy density.

### Emerging Carbon Materials

**Fig. 6:**
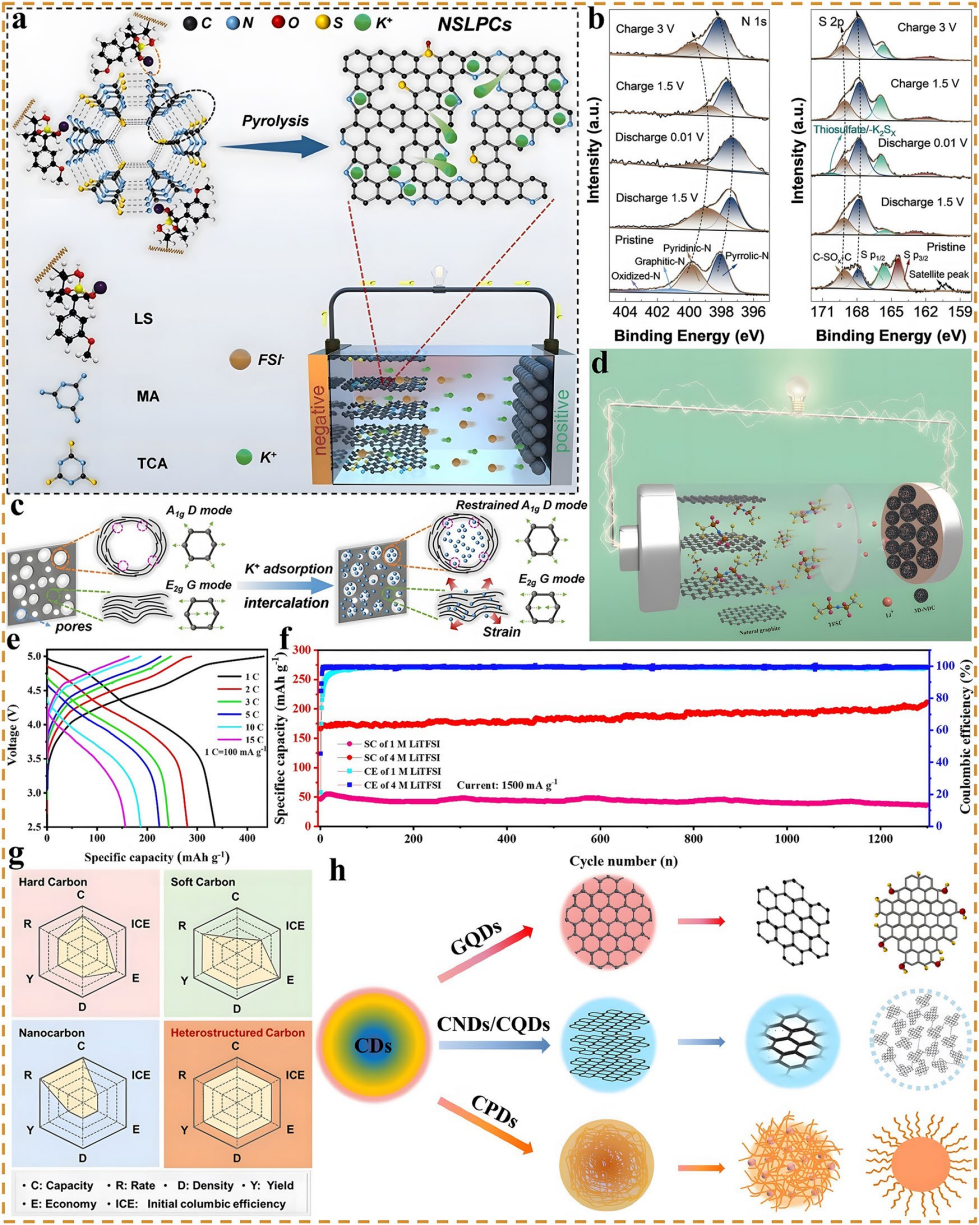
Application of emerging carbon materials in DIBs. **a** Schematic illustrating the configuration of the battery based on NSLPCs anode. **b** Ex situ N 1*s* spectra of NSLPC-700 anode during the charge/discharge process and **c** K^+^ storage in amorphous defect-rich carbons, reproduced with permission [129]. Copyright 2023, Springer Nature. **d** Working mechanism of the full battery based on nitrogen-doped carbon microsphere anode and concentrated organic-IL mixed electrolyte. **e** Galvanostatic charge–discharge curves under different rates and **f** the long-term cycling performance under 15 C, reproduced with permission [63]. Copyright 2023, Wiley. **g** Comparison of the characteristics and energy storage performance of hard, soft, nanostructured and heterostructure carbon, reproduced with permission [135]. Copyright 2021, Wiley. **h** Four types of fluorescent CDs, reproduced with permission [139]. Copyright 2021, Elsevier

Compared with the above-mentioned carbon materials, several emerging carbon materials may exhibit better energy storage capability owing to their unique physicochemical properties, thus making them potentially attractive host materials for cations, such as locally graphitized carbon, heteroatom-doped amorphous carbon, heterostructure carbon and carbon quantum dots. As one of the representatives, locally graphitized carbon acts between graphitized and non-graphitized, while combining the advantages of both [121, 122]. The locally ordered graphitized carbon (LOGC) interconnected with disordered carbon is able to weaken the van der Waals interactions between adjacent graphene sheets, which enhances the transport kinetics of reactive ions and reduces the corresponding diffusion energy barriers [71, 121]. Conversely, the non-graphitized disordered carbon regions not only enable the interconnection of dispersed nanographite domains, but also partially buffer the volume expansion effect caused by the severe ion interactions and provide additional capacitive active storage sites. Yang et al. compared the proof-of-concept potassium-based DIBs systems based on three typical carbon materials (fully graphitized carbon: graphite, non-graphitized disordered carbon: AC and locally ordered graphitized carbon: LOGC) [121]. Experiments and calculations concluded that the graphite-anode-based DIBs exhibit a low SDC of only ~ 50 mAh g^−1^, while the AC-anode-based DIBs also only show a slightly higher SDC of ~ 70 mAh g^−1^. Surprisingly, DIBs based on the LOGC anode display an ultra-high SDC of 232 mAh g^−1^ at 100 mA g^−1^ within 1.5–4.5 V, which shows no degradation after 1000 cycles at 300 mA g^−1^, further proving the great advantage of LOGC. The role of doping effects in carbon materials has also been comprehensively investigated, and the heteroatom doped amorphous carbon materials (HDCMs) are widely employed in the field of electrochemical storage as important candidates [123–125]. By controlling the content and typology of heteroatom-containing reagents, the physicochemical properties of the materials not only can be tuned, but also the morphology can be modulated to optimize the specific surface area and pore volume, thus improving the electrochemical performance of HDCMs [126–128]. Zhao et al. Prepared lignin-derived mesopore-rich disordered carbon materials with 21.6 at.% N doping and 0.8 at.% S doping via employing a novel supramolecular-mediated pyrolysis strategy [129]. In this regard, high N doping induces the generation of abundant defects and active sites, which in turn promotes K^+^ adsorption and reaction kinetics (Fig. 6a). Besides, it is proved that mesopores can also store a considerable number of reactive ions and inhibit the system swelling caused by ion insertion [130, 131]. As shown in Fig. 6b, X-ray photoelectron spectroscopy (XPS) demonstrated that pyridine nitrogen and pyrrole nitrogen undergo a blueshift with decreasing potential, which can be attributed to the large amount of K^+^ adsorbed by the disordered defect structure and active sites. As the potential recovered, a redshift happens and returns to the initial position, a similar phenomenon was observed for S 2*p*. With the decrease and increase of the potential, the reversible redshift and blueshift phenomenon occurs in Fig. 6c. The designed proof-of-concept full batteries exhibit a SDC of 45 mAh g^−1^ at a high rate of 1 A g^−1^ and are capable of stable cycling for 2000 cycles with a CR of 91%. DFT calculations and experiments indicate that only edge-nitrogen doping can induce the creation of defects and active sites, whereas nitrogen oxide with graphitized nitrogen is able to enhance the conductivity and provide a certain pseudocapacitive effect [132]. On this basis, a class of 3D amorphous hierarchical porous carbon microspheres were engineered through one-step direct pyrolysis of melamine foams with high total nitrogen and edge-nitrogen levels of 36.46% and 29.8%, respectively [63]. Such a high edge-nitrogen content can yield plentiful defects and edge-active site, which is expected to contribute to a high discharge capacity. Furthermore, a novel high-concentration organic solvent-ionic liquid hybrid electrolyte (4 M-EMC-Pyr_14_TFSI-5%ES, 1:1, v/v) is designed (Fig. 6d). This novel high-concentration electrolyte not only broadens the electrochemical window and increases the oxidation potential, but also alters the solvated structure of Li^+^, significantly increasing the proportion of anions in the solvated sheath. This anion-dominated solvation structure facilitates the formation of a denser inorganic-rich interfacial layer to passivate the anode, which in turn leads to an improved electrochemical performance. The proposed proof-of-concept DIBs not only offer an ultra-high SDC of 351 mAh g^−1^ at 100 mA g^−1^, but also exhibit good rate capability, maintaining stable GCD curves and smooth charge/discharge platforms at high current densities in Fig. 6e. Even at a high rate of 15C, the full DIBs still provide a stable cycling life up to 1300 cycles without degradation (Fig. 6f), which is one of the best results for the reported Li-based DIBs.

Heterostructure carbon is a kind of functional carbon material with special structure and favorable performance composed of two or more carbon layers with different properties, in which the various carbon layers share different carbon crystal structures, physicochemical characteristics and performance. Therefore, heterostructure carbon materials are capable of performing multiple unique functions simultaneously and can showcase more superior electrochemical performance than carbon materials with a specified structure [133–135]. As illustrated in Fig. 6g, heterostructure carbon is one of the promising functional materials for energy storage, as it is expected to bring improved comprehensive competitive advantages in various aspects (e.g., capacity, rate, ICE, yield, economy, and cycling stability). Shen et al. designed a high edge-nitrogen-doped carbon nanofiber/g-C_3_N_4_ heterostructure carbon material by electrostatic spinning and high-temperature pyrolysis sequentially, which show superior K^+^ storage performance, outstanding rate capability and satisfactory cyclic life [136]. When 3 M KFSI-DME was employed as electrolyte, the full batteries can deliver a high SDC of 106 mAh g^−1^ with an energy density of 62.3 Wh kg^−1^ (based on the total mass of cathode and anode) at 1 A g^−1^ and are stable for 5000 cycles at 5 A g^−1^ with a CE close to 100%. The synthesis and application of carbon dots (CDs) have developed into a dynamic and exciting emerging research field in recent years, and the 2023 Nobel Prize in Chemistry was awarded to the discovery and synthesis of quantum dots, further proving the extraordinary significance and application of CDs. CDs are a class of zero-dimensional carbon nanomaterials with distinctive properties, which consist of ultra-fine, dispersed, quasi-spherical carbon nanoparticles with sizes below 10 nm [137, 138]. As depicted in Fig. 6h, according to the difference of carbon nuclei, CDs can be classified into four categories, namely graphene quantum dots (GQDs), carbon quantum dots (CQDs), carbon nanodots (CNDs), and carbon co-polymer dots (CPDs). Especially, CDs are widely adopted in the field of advanced rechargeable batteries owing to their diverse and fascinating physicochemical properties, structures, and excellent electrochemical activities, which makes them promising electrode materials for energy storage and conversion [139]. Feng et al. prepared a class of GQDs materials through an electrochemical stripping method and employed them as both cathode and anode of DIBs, in which the electrolyte used was 2 M NaPF_6_-DEGDME, which can realize the stable and reversible insertion/desertion of both anions and cations [140]. The designed sodium-based DIBs exhibited an operating voltage of 4.0 V, a SDC of 68 mAh g^−1^, and a high ED of 250 Wh kg^−1^ (based on the total mass of cathode and anode) at 300 mA g^−1^ and can be cycled stably for 200 cycles with a CR of 95%, which represents one of the best performances reported at that time.

In summary, although carbonaceous materials possess the advantages of low cost, environmental friendliness, wide sources, and low insertion potential, they still suffer from some limitations, such as lower theoretical capacity, poor rate performance and structural stability, and higher self-discharge rate. It is possible to improve the specific discharge capacity, cycling performance and inhibit the spontaneous detachment of active ions from the electrode structure from the viewpoint of developing novel carbonaceous materials such as carbon-based materials or carbon-quantum dots with a large layer spacing, surface-active functional group, heterogeneous element doping, a high degree of disorder and defects, and a low interlayer van der Waals effect force. Furthermore, carbonaceous materials are able to store more cations as anode compared to the limited storage of anions at the cathode, which inevitably leads to a capacity mismatch between the cathode and anode. Generally, it is necessary to increase the active mass loading on the cathode side or decrease the loading on the anode side to achieve an active mass loading ratio of 3:1 or even higher for the cathode and anode. Further thinking is needed in the future on how to solve the capacity mismatch problem more rationally. For the convenience of comparison and better understanding by researchers, the configuration and electrochemical performance of DIBs based on various carbonaceous anode materials are listed in detail in Table 1.

**Table 1:** Configurations and electrochemical performance of DIBs based on carbonaceous anode materials

Type	Anode//Cathode Configuration	Electrolyte systems	SDC (mAh g^−1^)	Cyclic performance	Energy density (Wh kg^−1^)	Refs.
GC	Graphite power// Graphite power	2 M LiPF_6_-3 wt% VC	90 at 5C	96% after 1000 cycles at 5C	227 (based on anode and cathode)	[75]
	MCMB//CGP-A12	1.7 M LiPF_6_-FEC-EMC- 5 mM HFIP	80 at C/7	100% after 50 cycles at C/20	/	[76]
	Soft carbon //Graphite	1 M NaPF_6_-EC-DMC	103 at 2C	81.2% after 800 cycles at 10C	/	[77]
	Graphite// Graphite	KFSI-TEP-1:1.3-n:n	45.5 at 1C	98% after 350 cycles at 10C	/	[78]
	EG//Graphite	5.2 m KFSI-TMS	83.4 at 1C	100% after 300 cycles at 1C	130 (based on anode, cathode and electrolyte)	[79]
	Composite graphite//KS6	PP_14_TFSI	82 at 0.3C	100% after 600 cycles at 3C	258 (based on anode)	[80]
	MTI SAG-R//Graphite	3 M LiPF_6_-EMC-5 wt% LiDFOB	103 at 1C	92.4% after 6500 cycles at 10C	179.8 (based on anode and cathode)	[84]
	LTO-modified MCMB//EG	1.0 m LiPF_6_-EMC/SL-1:4	86.9 at 5C	93.5% after 1000 cycles at 5C	200 (based on anode and cathode)	[85]
	N-LIDG-800//Graphite	1 M LiPF_6_-EC-PC-1:1	240 at 1C	75% after 2400 cycles at 10C	/	[86]
NGC	WSC-1200//KS6	1 M NaPF_6_-EC-DMC-1:1	246 at 0.5C	78.4% after 3000 cycles at 50C	172.1 (based on anode and cathode)	[105]
	Hard carbon// Graphite	NaFSI-TMP-1:2-n:n	46.6 at 5C	86.8% after 300 cycles at 1C	/	[106]
	SP-HC 1200//Graphite	0.8 M KPF_6_-EC-DEC-1:1	67 at 1.3C	90% after 300 cycles at 1.67C	/	[107]
	NOCNBs//EG	0.8 M KPF_6_-EC-DEC-1:1	89 at 1C	95% after 200 cycles at 1C	/	[108]
	Activated carbon//KS6	LiFSI/LiTFSI-9–1-n:n-H_2_O	72 at 5C	100% after 100 cycles at 5C	214 (based on anode)	[113]
	SCN-A//SCN-A	1 M NaClO_4_-EC-DEC-1:1	47 at 1C	82% after 3000 cycles at 50C	112 (based on anode and cathode)	[114]
	N-AHCSs//EG	1 M-KPF_6_-EC-DEC-PC	89.8 at 1C	96.6% after 200 cycles at 1C	/	[115]
	Activated carbon//ANCS	0.3 M Mg(TFSI)_2_-Pyr_14_TFSI	55 at 0.5C	100% after 50 cycles at 0.5C	/	[116]
	Activated carbon//AEG	EMImPF_6_	/	83% after 50 cycles at 5C	43 (based on anode and cathode)	[117]
	ANCS//Graphite	1 M LiPF_6_-EC-DEC-1:1	67 at 100C	88.3% after 9000 cycles at 20C	206.7 (based on anode and cathode)	[118]
	P-HCNs-3//EG	1 M NaPF_6_-EC-DMC-1:1	158 at 5C	73% after 1200 cycles at 5C	138 (based on anode and cathode)	[119]
	CCNW//EG	1 M KPF_6_-EC-DEC-1:1	134.4 at 1C	100% after 1000 cycles at 5C	430 (based on the anode)	[120]
LOGC	LOGC//KB	1 M KPF_6_-EC-PC-1:1	232 at 0.5C	100% after 1000 cycles at 3C	/	[121]
HDCs	NSLPC//YP-50F	3 M KFSI-DME	45 at 10C	91% after 1000 cycles at 10C	71 (based on anode and cathode)	[129]
	3D-NDC// Natural graphite	4 M-EMC-Pyr_14_TFSI-1:1–5%ES	351 at 1C	100% after 1300 cycles at 15C	/	[63]
HSC	C_3_N_4_@NCNF// AC	3 M KFSI-DME	106 at 10C	/	62.3 (based on anode and cathode)	[136]
CDs	GQDs//EG	2 M NaPF_6_-DEGDME	68 at 3C	95% after 300 cycles at 3C	250 (based on anode and cathode)	[140]

## Metallic Materials

### Alloying-Type Materials

Compared with carbon-based materials, alloyed materials can undergo alloying reactions with metal ions to form metal binary compounds, thus exhibiting high theoretical capacity [141–143]. Meanwhile, the alloying materials can act as both anode and current collector, thereby reducing the inactive mass, which undoubtedly increases the energy density and is considered as one of the most promising anode materials for next-generation DIBs [144, 145]. The alloying reaction equations involved are as follows, where A is the alloying element and M^n+^ is the metal ion. Due to their nice inherent ability to store cations, these materials have attracted increasing attention in the field of DIBs.

**Fig. 7:**
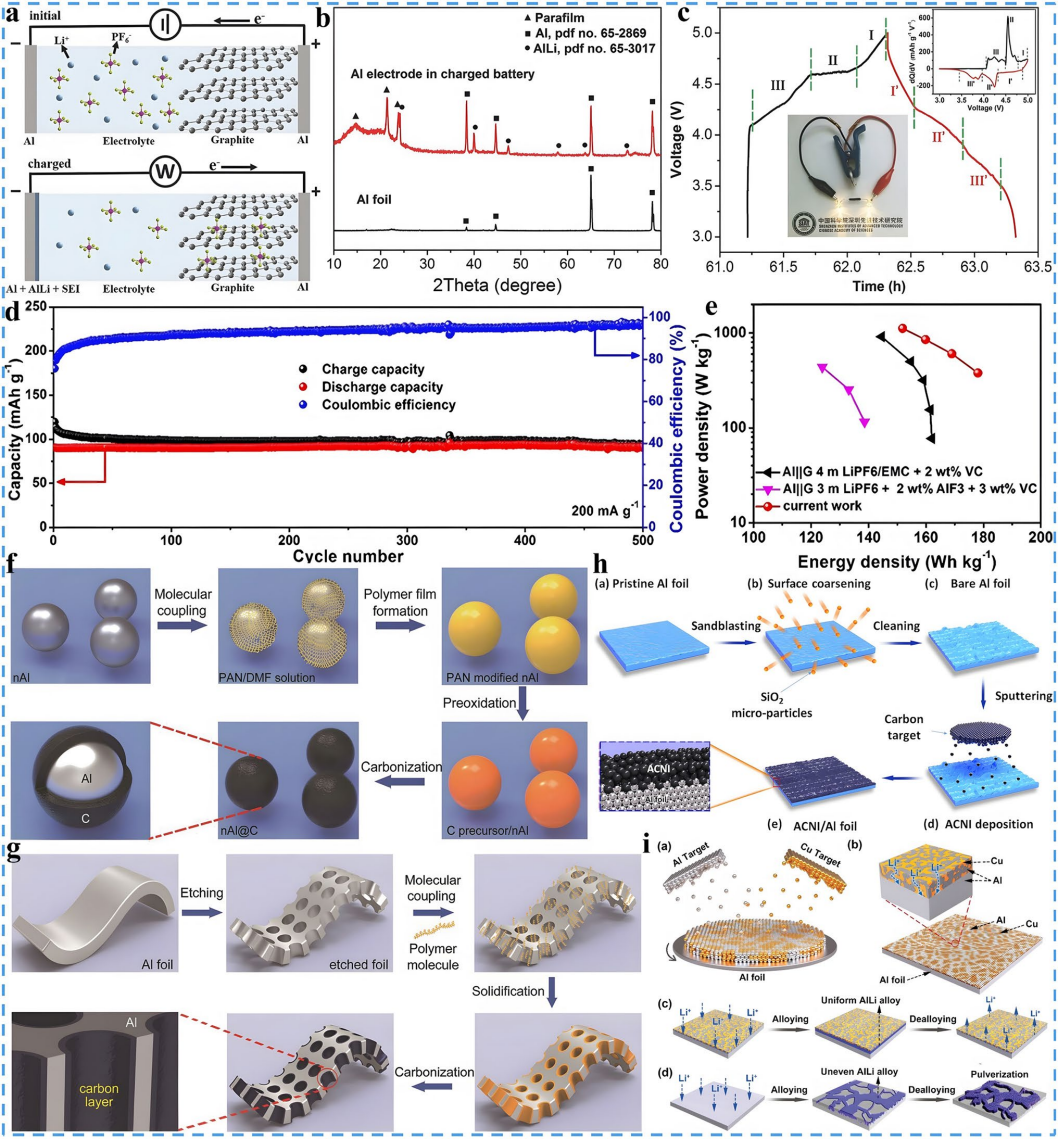
Alloying materials and corresponding modification strategies. **a** Schematic illustration of the AGDIB in the initial and charged state. **b** XRD patterns of a fresh Al foil and an Al electrode in a charged battery and **c** galvanostatic charge–discharge curve of the AGDIB at 0.5C, reproduced with permission [56]. Copyright 2016, Wiley. **d** Long cycling test of the DIBs and **e** Ragone plot of current work and previously reported results of DIBs based on 7.5 m LiFSI electrolyte at 200 mA g^−1^, reproduced with permission [147]. Copyright 2020, Wiley. **f** Schematic illustration of the fabrication process of nAl@C, reproduced with permission [151]. Copyright 2018, Wiley. **g** Schematic illustration of the fabrication process to make the pAl/C anode material, reproduced with permission [152]. Copyright 2016, Wiley. **h** Schematic illustration of the ACNI/Al anode fabrication process, reproduced with permission [153]. Copyright 2021, American Chemical Society. **i** Fabrication of the Cu–Al nanocomposite film on an Al anode and the schematic diagrams of the different Al anode structures, reproduced with permission [154]. Copyright 2019, Wiley

5 $${\text{xA}} + {\text{yM}}^{{{\text{n}} + }} + {\text{nye}}^{ - } \Leftrightarrow {\text{A}}_{{\text{x}}} {\text{M}}_{{{\text{ny}}}}$$

As a typical representative, Al is the first alloy-type material employed as the anode of DIBs, which boasts a satisfactory theoretical capacity (2980 mAh g^−1^) compared to that of graphite (372 mAh g^−1^). Moreover, Al displays superior electrical conductivity (3.8 $\times$ 10^7^ S m^−1^), ductility and low discharge potential, which is one of the most successful anode materials so far [56, 146]. Al was first applied as an alloy-type anode in the Li-DIBs system in 2016, as illustrated in Fig. 7a, during charging the following reactions (6–8) took place:

6 $${\text{Negative}}\;{\text{electrode}}:{\text{Al}} + {\text{Li}}^{ + } + {\text{e}}^{ - } \Leftrightarrow {\text{AlLi}}$$

7 $${\text{Positive}}\;{\text{electrode}}:{\text{xC}} + {\text{PF}}_{6}^{ - } \Leftrightarrow {\text{C}}_{{\text{C}}} ({\text{PF}}_{6} ) + {\text{e}}^{ - }$$

8 $${\text{Overall}}:{\text{Al}} + {\text{Li}}^{ + } + {\text{xC}} + {\text{PF}}_{6}^{ - } \Leftrightarrow {\text{AlLi}} + {\text{C}}_{{\text{C}}} ({\text{PF}}_{6} )$$

In which the Li^+^ come from electrolyte was deposited on the Al anode and undergoes alloying reactions, of which the formation of an AlLi alloy was proved by the X-ray diffraction (XRD) in Fig. 7b [56]. Simultaneously, PF_6_^−^ was intercalated into the graphite cathode, and it can be seen from the galvanostatic charge–discharge (GCD) curves and differential-capacitance curves in Fig. 7c that the process of PF_6_^−^ insertion/desertion from graphite was mainly composed of three main regions: in which the charging process is 4.08 ~ 4.59 V (stage III), 4.59 ~ 4.63 V (stage II), and 4.63 ~ 5.0 V (stage I); each region corresponds to a different anion insertion stage, respectively.

Specially, Al acts as both anode and current collector, greatly enhancing the mass/volume energy density. When natural graphite and 4 M-LiPF_6_-EMC-2%VC are employed as cathode and electrolyte, respectively, the constructed Gr//Al dual-ion batteries (AGDIBs) exhibit a high median voltage of 4.2 V, which can easily light up two LEDs with a nominal voltage of 2.5 V (insert of Fig. 7c). Moreover, the AGDIBs not only achieve an SDC of 104 mAh g^−1^ at 2C and a CR of 88% after 200 cycles, but also exhibit an ED of 222 Wh kg^−1^ (based on the total mass of cathode and anode) at a power density (PD) of 132 W kg^−1^, which is significantly higher than that of commercial LIBs. In order to further improve the energy density and cycle stability, a concentrated electrolyte system based on carbonate electrolyte (7.5 m LiFSI in EC-DMC, 1:1, v/v) was developed [147]. Compared with conventional electrolytes, this high-concentration electrolyte features several advantages:

1. LiF-rich SEI layer can be formed to optimize the structural stability of the Al anode.
2. The oxidation stability of electrolyte is greatly improved, and the insertion capability and cycling stability of the graphite cathode are also enhanced.
3. The dosage of electrolyte is reduced, and the energy density of DIBs is increased.

Accordingly, the proof-of-concept AGDIBs based on this concentrated electrolyte display a SDC of 94 mAh g^−1^ at 200 mA g^−1^ with a CR of up to 96.8% after 500 cycles (Fig. 7d). Furthermore, calculated based on the total mass of electrodes and electrolyte, the ED of AGDIBs is as high as 180 Wh kg^−1^ (based on the electrode materials and electrolyte), which is considerably higher than that of commercial LIBs in Fig. 7e.

However, Al anode not only suffers from severe volume expansion (100% for AlLi) and structural pulverization during alloying/dealloying, but also forms lithium dendrites and generates large amounts of dead lithium. On the one hand, this will lead to an increasing impedance and a sharp decline in capacity and cyclic stability. On the other hand, lithium dendrites would easily puncture the separator, leading to an explosion in serious cases, which brings a certain safety hazard and seriously hampers the practical application of Al-based DIBs [148–150]. Therefore, it is crucial to overcome these challenging problems through wide-ranging techniques and strategies. Encouragingly, promising solutions have been devised to avoid battery failures caused by imperfect aluminum foil anode. First and foremost among them are morphology and structure regulation strategies such as core–shell structure and porous structure design, which are considered to be extremely viable approaches. Tong et al. developed a scalable and low-cost method to synthesize core–shell Al@carbon nanorods (nAl@C) anode with a mangosteen-like structure [151]. The specific synthesis steps are depicted in Fig. 7f, as a result, the nanoscale framework of nAl@C consists of aluminum nanorods and a surface amorphous carbon layer, which is about 5 nm thick and highly conductive and protective, contributing to the formation of a stable SEI film. Compared with pure Al foil, nAl@C shows a better adaptation to mechanical strains and stresses, which enables effective suppression of volume expansion and structural comminution. The DIBs based on the nAl@C anode exhibit good cyclic stability, with up to 94.6% CR even after 1000 cycles at a high rate of 15C in the voltage range of 3–5 V. SEM and TEM characterizations demonstrate that nAl@C still maintains favorable structural integrity. In addition, a 3D porous aluminum foil (pAl/C) coated with a carbon layer was designed as anode and current collector, as illustrated in Fig. 7g, in which the unique porous structure not only enlarges the contact area with electrolyte and offers a rapid active ion and electron transport channel, but also effectively prevents the volume expansion caused by the AlLi alloying process [152]. The constructed DIBs achieve a CR of 89.4% even after 1000 cycles at a low rate of 2C and provide a satisfactory ED of 232 Wh kg^−1^ (based on the total mass of cathode and anode) at a PD of 446 W kg^−1^. The strategy of fabricating artificial SEI also holds great promise for improving the structural stability of the Al anode and inhibiting the excessive decomposition of electrolyte. As shown in Fig. 7h, an amorphous carbon nanointerface (ACNI) with a thickness less than 10 nm was deposited as a functional nanofilm on the surface of Al foil by direct-current magnetron sputtering (DC-MS) [153]. The ACNI acts as an artificial SEI, which not only possesses high mechanical strength and favorable structural stability, but also effectively restrains the continued growth of the already-generated SEI to modify the kinetics of electrochemical reactions. The ACNI anode-based full batteries deliver a SDC of 115 mAh g^−1^ at 2C and are stable for 1000 cycles with a CR of 94%. Interfacial modification engineering is also a simple but effective strategy [154–156]. Figure 7i demonstrates one of the approaches, which employs inactive Cu to modify the interface of active Al and form a Cu-Al heterojunction nanoalloy material (Cu-Al@Al) via co-deposition [154]. Compared with the unmodified Al, Cu-Al@Al not only notably reduces the Li^+^ diffusion barrier and inhomogeneous deposition sites to achieve a homogeneous alloying reaction, but also facilitates the dispersion of volume expansion stresses to improve the structural stability of the Al anode. As a result, the assembled full batteries could achieve 200 stable cycles with a CR of 88% even with an electrode active mass load of up to 7.4 mg cm^−2^, implying the remarkable electrochemical performance far beyond that of commercial LIBs.

**Fig. 8:**
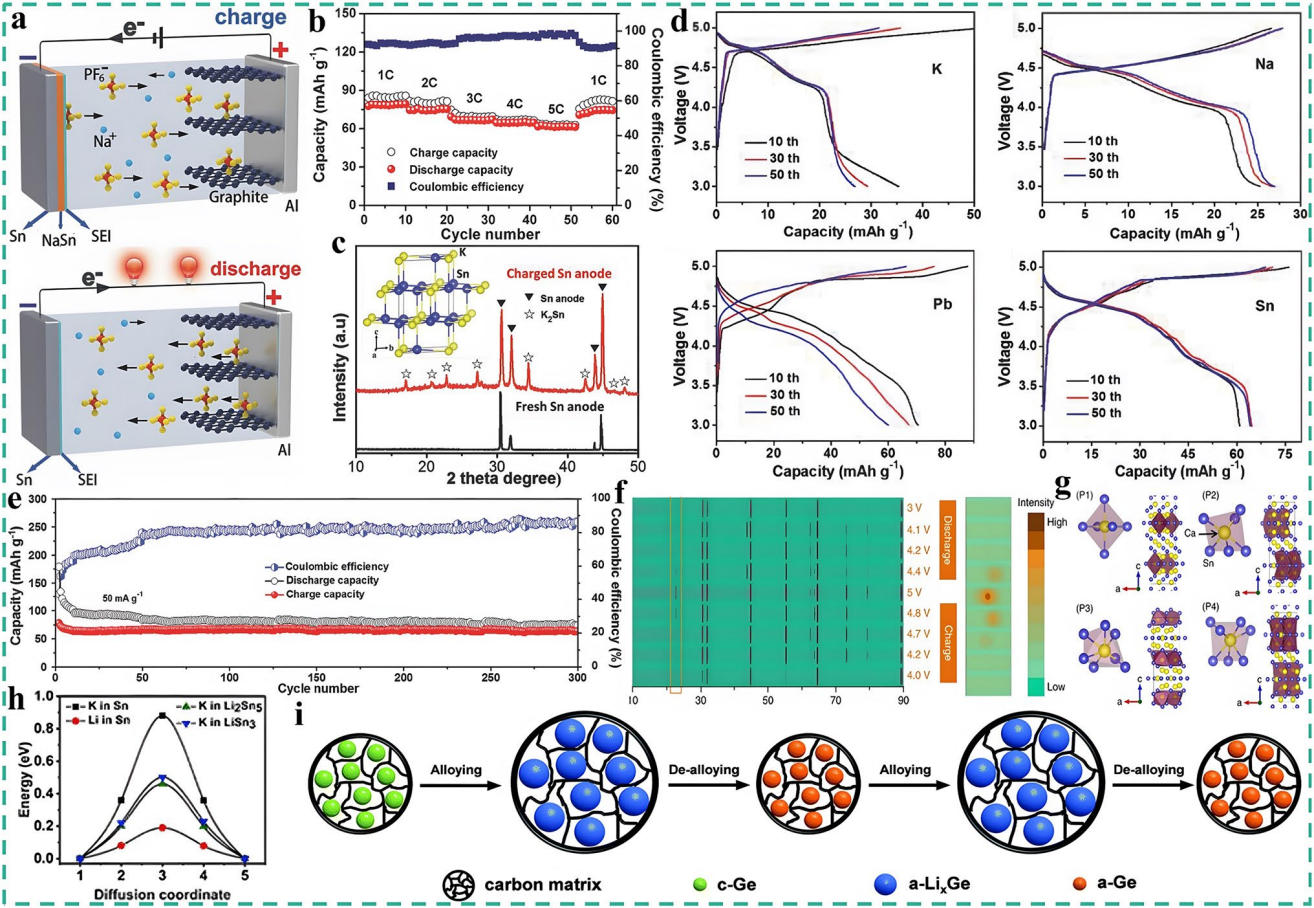
The application and modification strategies of Sn and non-metallic alloys in DIBs. **a** Schematic illustration of the working mechanism of Sn-G DIB and **b** charge/discharge capacities and corresponding Coulombic efficiencies of at various rates, reproduced with permission [57]. Copyright 2016, Wiley. **c** XRD profiles of a fresh Sn foil anode and a Sn anode in a charged battery, the inset shows the crystal structure of K_2_Sn. **d** GCD curves of the K-DIBs with K, Na, Pb, and Sn metal foils as anode and **e** long cycling performance at 50 mA g^−1^ for 300 cycles, reproduced with permission [58]. Copyright 2017, Wiley. **f** XRD profiles of a Sn anode cycled to different charge states and the detailed view of the boxed area showing the XRD patterns of a Sn anode from 22° to 24°. **g** Four different bonding situations for Ca and Sn in the Ca_7_Sn_6_ alloy, reproduced with permission [59]. Copyright 2018, Springer Nature. **h** Diffusion energy barriers of K in Sn, Li_2_Sn_5_, and LiSn_3_ crystals, and Li in the Sn crystal, reproduced with permission [161]. Copyright 2021, Chinese Chemical Society. **i** Proposed schematic to reveal the phase transformation for the Ge/CNF electrode during the cycling process, reproduced with permission [163]. Copyright 2020, Royal Chemical Society

Compared with the Al anode, Sn exhibits higher reactivity and theoretical capacity for non-Li metal cations such as Na^+^, K^+^, Ca^2+^, and Mg^2+^, as well as lower cost and environmental friendliness. So it is also an appealing alloy-type candidate that has been widely utilized in DIBs [157–159]. Tang et al. took the international lead in applying Sn as an anode in different DIBs systems such as Na-based [57], K-based [58], and Ca-based DIBs [59]. As for Na-based DIBs, his group employed expanded graphite (EG) as the cathode and 1 M NaPF6-EC-EMC-DMC (1:1:1, v/v/v) as the electrolyte [57]. As presented in Fig. 8a, during the charge process, Na^+^ in the electrolyte migrates to the anode and participates in the subsequent alloying reaction with Sn to form NaSn alloy.

9 $${\text{Sn}} + {\text{Na}}^{ + } + {\text{e}}^{ - } \Leftrightarrow {\text{NaSn}}$$

At the same time, PF_6_^−^ shifts to the cathode and intercalates into the EG to form graphite-intercalated compounds (GICs). On the contrary, during the discharged state, Na^+^ undergoes a dealloying reaction, and PF_6_^−^ is de-intercalated from EG back into the electrolyte. The constructed Sn-G DIB exhibits excellent cyclic stability and outstanding rate capability as presented in Fig. 8b, and the GCD curves at different rates show clear charge/discharge platforms with small polarization effects. Particularly, Sn acts as both the anode and current collector, which reduces the dead load and dead volume, and this is beneficial for improving SDC and ED. The Sn-G DIB achieves a SDC of 74 mAh g^−1^ at 2C and a high CR of 94% after 400 cycles at 2C in the 2–4.8 V operating voltage range, as well as an ED of 144 Wh kg^−1^ (based on the mass of cathode) at a PD of 150 W kg^−1^. Similarly, for the K-based DIBs, during charging, K^+^ reacts with Sn to form K_2_Sn alloy as proved by XRD in Fig. 8c, the involved reaction equation is as follows:

10 $${\text{Sn}} + 2{\text{K}}^{ + } + 2{\text{e}}^{ - } \Leftrightarrow {\text{K}}_{2} {\text{Sn}}$$

Furthermore, the electrochemical performances based on K, Na, Pb and Sn metal anodes are comprehensively compared [57–59]. Figure 8d represents the corresponding GCD curves for various systems where the anode and electrolyte are EG and 1 M KPF_6_-EC-DMC-EMC (4:3:2, v/v/v), respectively, and it can be seen that all of the systems exhibit reversible electrochemical activities with various degrees [58]. However, the DIBs based on K and Na foil anodes both show low SDCs (< 30 mAh g^−1^), and the Pb foil anode-based DIBs, although possessing a relatively high SDC, the cyclic stability is poor, with the discharge platform gradually disappearing during cycling, and the polarization effect is intensified. Conversely, the DIBs based on Sn foil anode not only display the highest SDC and the best electrochemical performance, but also the GCD curves at different cycles basically overlap with minimal polarization. A 93% CR is achieved after 300 cycles at 50 mA g^−1^ (Fig. 8e), and an ED of 155 Wh kg^−1^ (based on the mass of cathode) is provided at a PD of 116 W kg^−1^. Inspiringly, Sn can even undergo a stable and reversible alloying reaction with Ca^2+^. The feasibility of the Ca-based dual-ion battery based on the Sn anode, natural graphite (NG) cathode and 0.8 M Ca(PF_6_)_2_-EC-PC-EMC-DMC (1:1:1:1, v/v/v/v) electrolyte is verified to be able to work stably at room temperature within a high-voltage window of 3–5 V, breaking through the bottleneck of room-temperature irreversibility in Ca-ion batteries [59]. As shown in Fig. 8f, in situ XRD was employed to trace the alloying/dealloying reaction of Ca^2+^ with Sn. At the onset, only diffraction peaks corresponding to tetragonal Sn are observed, when further charged to 4 V, a new characteristic peak appears at 22.8°, which is attributed to the (201) peak of Ca_7_Sn_6_, indicating that Ca^2+^ undergoes alloying reaction with Sn. As charging continues, the intensity of the (201) peak increases and reaches its strongest level when fully charged, and the signal of the (201) peak diminishes during the subsequent discharge process, suggesting that a dealloying reaction has occurred. The signal does not disappear completely after being fully discharged, which is due to the slow kinetics of the solid-state electrochemical reaction. Additionally, four possible crystal structures of Ca_7_Sn_6_ in Fig. 8g are simulated by DFT, two of which are a Ca atom surrounded by six nearest Sn atoms to form a distorted octahedron (P1 and P2), while the other two are surrounded by seven nearest Sn atoms and form a distorted decahedron (P3 and P4). Furthermore, the binding energies of P1-P4 are calculated separately, all of which are negative and P1 > P2 > P3 > P4, indicating that P4 possesses the most stable structure, which is consistent with the reversible alloying/dealloying process between Ca^2+^ and Sn. Finally, the as-constructed Ca-DIBs achieve an average discharge voltage of up to 4.45 V with a SDC of 72 mAh g^−1^ at room temperature and exhibits excellent stability with a 95% CR for 350 cycles at 100 mA g^−1^, which is considerably higher than other reported performance on Ca-ion batteries. In addition to the above-mentioned metal cations, Sn can react with divalent Mg^2+^ in a reversible alloying reaction, and the Mg-based DIBs designed on the basis of this reaction principle display exceptional electrochemical performance [160]. The corresponding SDC at 100 mA g^−1^ is 133 mAh g^−1^, and the CR after 1000 cycles at 1000 mA g^−1^ is up to 97.3% with an ED of 394 Wh kg^−1^ (based on the mass of cathode) at the PD of 293 W kg^−1^. Unfortunately, Sn experiences more severe volume expansion (120% for NaSn) and structural pulverization during alloying than that of Al anode, ultimately leading to a battery failure. Similarly, the above strategies used to improve Al alloy are equally applicable to Sn. Jiang et al. proposed an ionic-drill strategy to enhance the K-Sn alloying reaction [161]. Specifically, 20 atom% Li^+^ was added to the K^+^-based electrolyte, this small portion of Li^+^ exhibits a lower diffusion energy barrier and faster reaction kinetics than K^+^ and can act as an ionic drill, thus preferentially alloying with Sn to form tin-rich phases such as Li_2_Sn_5_ and LiSn_3_, which significantly decreases the diffusion energy barrier of K^+^ (Fig. 8h), opening up the diffusion channel and accelerating the kinetics for K^+^ considerably. Moreover, the introduction of Li^+^ can also form a LiF-rich SEI layer on the Sn surface with higher mechanical strength, which can effectively inhibit the volume expansion caused by K-Sn alloying. Thanks to the ion-drill strategy, the constructed KDIBs showed unprecedented results, with a SDC of 106 mAh g^−1^ and a median voltage of 4.1 V at 5C and can be stably cycled for 500 cycles without capacity degradation.

In addition to Al and Sn, there are other metals that are capable of reversible alloying reactions with metal cations. Zhang et al. reported a heteroatom-doped hierarchical porous antimony nanoparticle/carbon nanofiber-modified Sb alloy (HPSbCNFs) as a flexible freestanding anode for high-performance K-DIBs [162]. HPSbCNFs with extraordinary mechanical flexibility, hierarchical porous structure and high content of nitrogen doping can mitigate volume variation during alloying, enhance electronic and ionic conductivity and supply additional active sites for K^+^ storage. The fabricated K-DIBs deliver an ultra-high SDC of 440 mAh g^−1^ with a high medium discharge voltage of 4.5 V at 200 mA g^−1^ and can be cycled stably for 1440 cycles at 500 mA g^−1^. While Zhou et al. integrated Ge nanoparticles into one-dimensional (1D) carbon nanofiber to form a nanocomposite Ge/CNFs anode and applied it to Li-DIBs (Ge/CNFs-G DIB) [163]. As shown in Fig. 8i, during the charge process, the initial crystalline Ge experiences a phase transition to form an amorphous Li_x_Ge alloy, and then the amorphous Li_x_Ge alloy is converted to amorphous Ge by dealloying. Besides, the 1D carbon nanofibers can effectively suppress the volume expansion induced by the alloying process and maintain the structural stability, as well as shorten the Li^+^ diffusion pathway and improve the conductivity. The corresponding Ge/CNFs-G DIB exhibited a SDC of 281 mAh g^−1^ at 250 mA g^−1^ and are capable of stable cycling for 500 cycles at 2500 mA g^−1^. Moreover, as a powerful supplement to the non-metallic alloying-type anode materials, Si and red phosphorus also possess superior intrinsic ability to store metal cations [164–168]. Among them, the alloy reaction equations of Si and Li are as follows:

11 $${\text{Si}} + {\text{xLi}}^{ + } + {\text{xe}}^{ - } \Leftrightarrow {\text{Li}}_{{\text{x}}} {\text{Si}}$$

However, the volume expansion of Si after complete lithiation can be up to 300%, which severely hampers the application of the Si anode in DIBs. To this end, a flexible interface design strategy that can adjust stress distribution is proposed [169]. By constructing the Si anode on a soft nylon fabric modified with a conductive Cu-Ni transition layer, the flexible interface between Si and soft polymer substrate is rationally devised to regulate the alloying stress of the Si anode, which endows Si with remarkable flexibility and stability, thus greatly improves the structural stability of Si. The designed DIBs achieve a record-breaking ultra-high rate capability of 150C with a SDC of 96 mAh g^−1^ at 2C and a high CR of 97% after 2000 cycles at 10C. Red phosphorus has been proven to be an excellent Na^+^ storage anode material due to its low electrochemical plateau, high theoretical capacity and elemental abundance. Yu et al. prepared RP/CNT@GO composite anode via ultrasound-self-assembly, paired with the graphite cathode and 1 M NaPF_6_-EC-DMC-EMC (1:1:1, v/v/v) electrolyte, and the corresponding proof-of-concept P-G SDIBs exhibit an operating voltage of 3.9 V [170]. The alloying reaction between Na^+^ and red phosphorus during charging and discharging is demonstrated as follows by XRD and XPS techniques:

12 $$3{\text{Na}}^{ + } + {\text{P}} + 3{\text{e}}^{ - } \Leftrightarrow {\text{Na}}_{3} {\text{P}}$$

Furthermore, the P-G SDIBs delivered a reversible capacity of up to 373 mAh g^−1^ with an ED of 176 Wh kg^−1^ (based on the total mass of cathode and anode) at 200 mA g^−1^ and are capable of stable cycling for 400 cycles.

### Conversion-Type Metal Materials

**Fig. 9:**
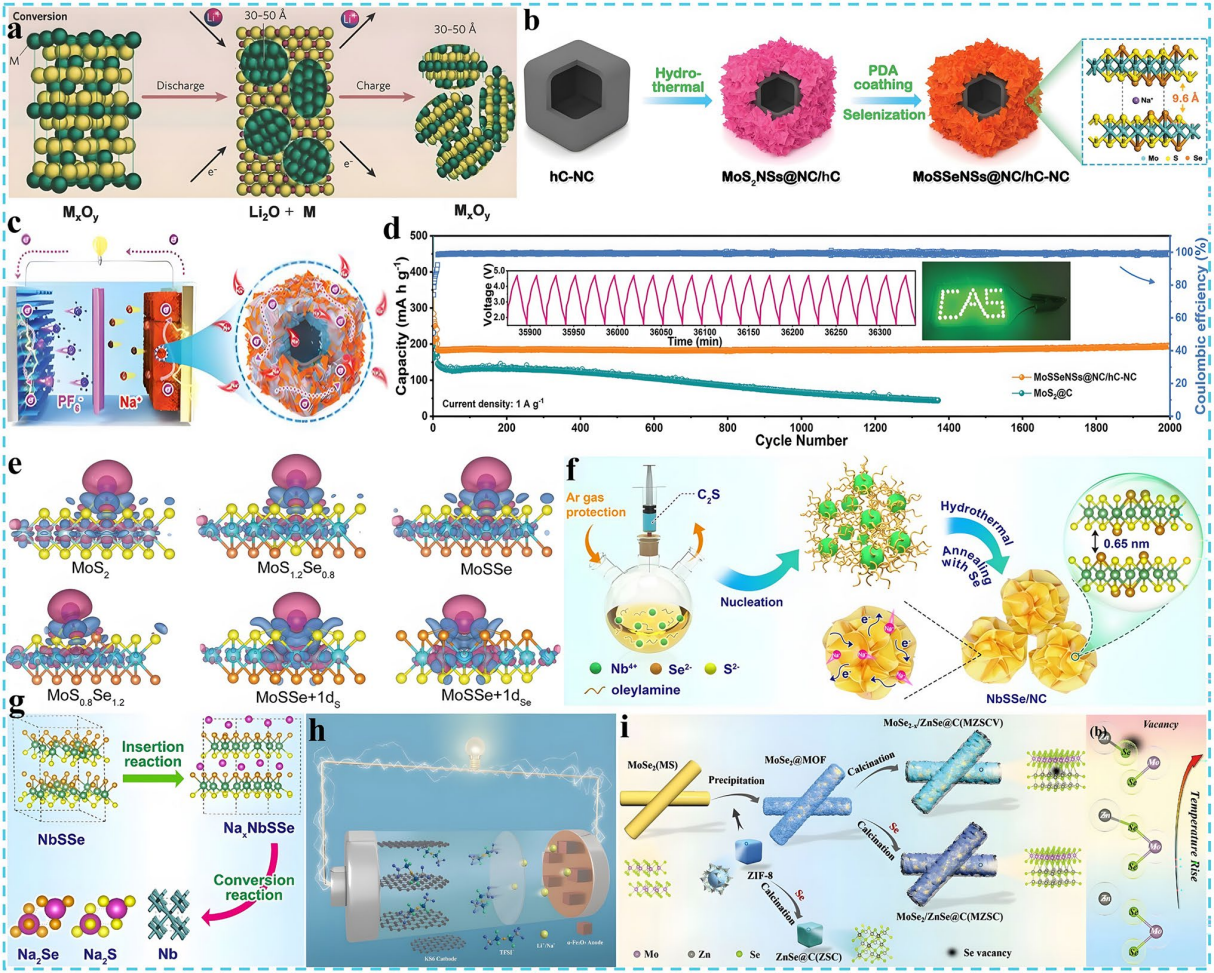
Conversion materials and the corresponding modification strategies. **a** Schematic of the reaction mechanism based on conversion reaction, reproduced with permission [174]. Copyright 2018, Wiley. **b** Schematic illustration for the synthesis process of MoSSeNSs@NC/hC-NC. **c** The operating principle of DIBs based on MoSSeNSs@NC/hC-NC anode. **d** Long-term cycling stability at 1 A g^−1^. **e** Electron density differences of intercalated Na in MoS_2_, MoS_1.2_Se_0.8_, MoSSe, MoS_0.8_Se_1.2_, MoSSe with one S defect and MoSSe with one Se defect, reproduced with permission [179]. Copyright 2021, Wiley. **f** Schematic illustration for the synthesis process of NbSSe/NC and **g** corresponding electrochemical mechanism schematic, reproduced with permission [180]. Copyright 2023, Springer Nature. **h** Operating principle of DIBs based on nanocubic α-Fe_2_O_3_ anode, reproduced with permission [181]. Copyright 2022, Elsevier. **i** Schematic diagram of the fabrication process of the MoSe_2-x_/ZnSe@C, reproduced with permission [183]. Copyright 2023, Wiley

Conversion materials typically refer to transition metal sulfides, selenides, or oxides, etc., as shown in Fig. 9a [171–174]. These materials can perform a conversion reaction with active cations and undergo a phase decomposition process to transform into the metal elements and the corresponding sulfides or oxides, as presented in the following reaction formula:

13 $${\text{T}}_{{\text{a}}} {\text{N}}_{{\text{b}}} + {\text{bxM}}^{ + } + {\text{bxe}}^{ - } \Leftrightarrow {\text{aT}} + {\text{bM}}_{{\text{x}}} {\text{N}}$$

where T is a transition metal element, N is a non-metal element (S, O, Se, F, P, N), M is an alkali metal element (Li, Na, K) and x is the formal oxidation state of N. Conversion-type materials indicate promising prospects as anode materials for DIBs because of their high theoretical capacity and relatively long cycle life. Nevertheless, the unmodified conversion active materials commonly experience serious volume expansion and structural collapse after the phase transition or phase decomposition process, resulting in a low discharge capacity and poor cyclic stability in practice [175–178]. So it is urgent to adopt corresponding measures to overcome these drawbacks in order to boost the electrochemical performance. Wen et al. present a delicately designed nitrogen-doped carbon film-modified MoSSe nanosheets anode (MoSSeNSs@NC/hC-NC) loaded on hollow cubic nitrogen-doped carbon for efficient Na^+^ storage, which plays a role of "killing three birds with one stone" [179]. Specifically, as illustrated in Fig. 9b, the hollow cubic carbon skeleton and the nitrogen-doped carbon film realize the double carbon coating layer inside and outside, which not only prevent the volume expansion and enhance the structural stability, but also extend the contact area with electrolyte and promote the reaction kinetics. Moreover, Se doping not only creates abundant defects and adds active sites, but also enlarges the interlayer spacing and conductivity, which promotes the transport and storage of Na^+^. Compared with unmodified MoS_2_@C, the Na-DIBs based on MoSSeNSs@NC/hC-NC anode display high SDC, good rate capability and cyclic stability. On the basis of the operating principle in Fig. 9c, the DIBs provide a SDC of 150 mAh g^−1^ even at 5 A g^−1^ and exhibit a long lifetime of 2000 cycles at 1 A g^−1^ (Fig. 9d), as well as an ED of 101 Wh kg^−1^ (based on the total mass of cathode and anode) at 1212 W kg^−1^. Further, the Na^+^ storage behavior of MoSSeNSs@NC/hC-NC was explored by DFT calculations. Compared with pure MoS_2_, the binding energy ΔE is remarkably reduced when Se elements and corresponding anionic defects are introduced, and the charge-density difference model of Fig. 9e also indicates that the charge density is more likely to cluster around the sites of Se doping and defects with narrower band gap near the Fermi energy level, proving that Se doping and its induced defects provide a better Na^+^ adsorption capacity. To further improve the energy density, structural stability and intrinsic conductivity, a single-phase ternary NbSSe/NC nanocompound enriched with defective microstructure was prepared by the synthesis steps depicted in Fig. 9f [180]. NbSSe/NC possesses a wide layer spacing of 0.65 nm and a conductivity of 3.23 × 10^3^ S m^−1^, which is favorable for the transport of ions and electrons. A reversible conversion reaction happens during the charging process as shown in Fig. 9g, and the specific reaction equation is as follows:

14 $${\text{NbSSe}} + 4{\text{Na}}^{ + } + 4{\text{e}}^{ - } \Leftrightarrow {\text{Nb}} + {\text{Na}}_{2} {\text{S}} + {\text{Na}}_{2} {\text{Se}}$$

The wide layer spacing and abundant defect architectures enable the structure of NbSSe/NC to remain intact even after repeated phase transitions. The proof-of-concept DIBs show negligible capacity decay after 1000 cycles at 0.5 A g^−1^ and still deliver a high ED of 230.6 Wh kg^−1^ based on the total mass of the cathode and anode. Beyond strategies such as heteroatom doping and carbon coating, directly designing the target product to feature a specific morphology at the nanoscale is also beneficial in enhancing the performance of alloying materials. Wu et al. synthesized an α-Fe_2_O_3_ anode material with a nanocubic structure as exhibited in Fig. 9h through a nanostructure engineering strategy, which can display excellent Li^+^/Na^+^ storage performance and structural stability without any other modifications [181]. While the conversion reaction involved is as follows:

15 $${\text{Fe}}_{2} {\text{O}}_{3} + 6{\text{Li}}^{ + } /{\text{Na}}^{ + } + 6{\text{e}}^{ - } \Leftrightarrow 2{\text{Fe}} + 3{\text{Li}}_{2} {\text{O}}/{\text{Na}}_{2} {\text{O}}$$

The full batteries share a high SDC of 192 (Li-DIBs) and 197 (Na-DIBs) mAh g^−1^ at 5C, respectively, and there is no decay even after 520 cycles, which is also evidenced via TEM characterization that the structure of α-Fe_2_O_3_ is still stable and not destroyed. Furthermore, the self-discharge rate of the Na-DIBs system is as low as 0.1% h^−1^, which is notably lower than that of the reported DIBs based on conversion-type anodes. Zhu et al. have designed a class of MoS_2_ anode with penne-like structure using a microwave-solvothermal method, which shows enlarged layer spacing and enhanced structural stability relative to unmodified MoS_2_, thus improving the discharge capacity and cycle performance [182]. The constructed sodium-based DIBs provide a SDC of 65 mAh g^−1^ at 2C with a CR of 85% after 200 cycles. Heterostructure engineering is considered a promising improvement strategy in the field of energy storage in conversion materials as it can yield specific heterogeneous interfaces and ionic vacancies and promote the transfer of electrons and ions due to its powerful interfacial synergistic effect, which remarkably enhances the electrochemical performance and indicates an exceptional potential. Qian et al. introduced metal–organic frameworks (MOFs) from structurally ordered MoSe_2_ nanorods and finally formed a bimetallic molybdenum selenide/zinc selenide@carbon composite (MoSe_2-x_/ZnSe@C) with a coaxial heterojunction and vacancy structure induced by metal electronegativity for Na^+^ storage (Fig. 9i) [183]. The reversible electrochemical reactions occurred during charging and discharging as follows:

16 $${\text{MoSe}}_{2} + 4{\text{Na}}^{ + } + 4{\text{e}}^{ - } \Leftrightarrow {\text{Mo}} + 2{\text{Na}}_{2} {\text{Se}}$$

17 $${\text{ZnSe}} + 2{\text{Na}}^{ + } + 2{\text{e}}^{ - } \Leftrightarrow {\text{Zn}} + {\text{Na}}_{2} {\text{Se}}$$

18 $$13{\text{Zn}} + {\text{Na}}^{ + } + {\text{e}}^{ - } \Leftrightarrow {\text{NaZn}}_{13}$$

The DFT calculations present evidence that the heterogeneous structure improves the adsorption of MoSe_2-x_/ZnSe@C on reactive ions and facilitates the ion transport, while the vacancies enhance this effect and boost the conductivity. Moreover, in situ electrochemical impedance spectroscopy (EIS) demonstrates that MoSe_2-x_/ZnSe@C exhibits a high capacitive contribution with a tendency of decreasing charge-transfer impedance (Rct) value during charging, leading to a faster ion transport. Consequently, the full batteries exhibit a SDC of 209 mAh g^−1^ at 0.5 A g^−1^ and an ED of 131 Wh kg^−1^ (based on the total mass of cathode and anode) at 275 W kg^−1^, which can be cycled stably for 1000 cycles at 1 A g^−1^.

### Deposition-Type Metal Materials

Deposition-based anode materials, such as metals Li, Na, K, and Zn, can directly act as anode and collector for DIBs and display satisfactory electrochemical performance due to their high theoretical capacity, low reduction potential and superior conductivity [184–187]. Specifically, the following deposition/stripping reactions will occur during charging and discharging:

19 $${\text{M}}^{{{\text{n}} + }} + {\text{ne}}^{ - } \Leftrightarrow {\text{M}}$$

Among them, lithium metal shares the highest theoretical capacity (3862 mAh g^−1^), the lowest standard reduction potential (−3.04 V) and the smallest density (0.53 g cm^−3^), which holds a great application prospect for realizing high energy density DIBs. A kind of Gr//Li-DIBs based on 3 M LiPF_6_-EMC show a SDC of 95 mAh g^−1^ at 0.1C with good rate capability and high operating voltage, and still deliver a CR of 90% with an average CE of 98.5% at a high rate of 12C [188]. Besides, the Gr//Zn DIBs based on Zn metal anode and ionic liquid electrolyte offer an SDC of 57 mAh g^−1^ at 2C, a median discharge voltage of 1.6 V and a CR of 86% after 500 cycles [189]. However, these metal anodes could cause the formation of metal dendrites and dead metals during the deposition/stripping process, which would undoubtedly result in the continuous consumption of electrolyte and the re-formation of SEI, thus leading to a rapid decay of capacity and CE [190–192]. More seriously, it may puncture the separator and cause safety hazards such as short circuit or even fire. Similarly, some relevant solutions can be drawn from the modification strategies of alloying and conversion metal materials, such as the engineering of morphological structures, the construction of protective interfaces and the selection and concentration of electrolytes and additives. Wu et al. proposed to fabricate a highly conductive and robust artificial protective layer on the surface of lithium metal, which can effectively prevent the formation of lithium dendrites and greatly enhance the stability compared to the unmodified lithium metal anode [193]. The designed Gr//Li-DIB boasts a SDC of 95.2 mAh g^−1^ at 1 A g^−1^ and a CR of 89.6% after 500 cycles. While Sun et al. introduced a LiNO_3_ additive into the carbonate electrolyte, which is able to form a dense and smooth SEI rich in high conductivity Li_3_N, which is highly favorable for Li^+^ transport and nucleation, and inhibits the generation of lithium dendrites and the decomposition of the electrolyte [194]. The resultant Li-G DIB realizes a SDC of 91 mAh g^−1^ at 2C with a CR of 97% after 300 cycles, and delivers an ED of 243 Wh kg^−1^ (based on the mass of cathode) at 234 W kg^−1^. Zheng et al. designed a type of Zn metal anode with a unique plate-like stacking structure through the strategy of epitaxial metal electrodeposition, which effectively reduces the lattice strain of Zn and alters the deposition pattern of Zn^2+^ [195]. This enables Zn^2+^ to be deposited preferentially parallel to the electrode surface during the reaction process, which successfully avoids the formation of Zn dendrites and improves the cycling stability.

Metallic anode materials are expected to bring excellent electrical conductivity, high discharge capacity and superior energy density, but the serious volume expansion effect, structural exfoliation and pulverization, irreversible intermediate phases, and the generation of metallic dendrites will occur during charging and discharging, which result in electrochemical performance degradation and battery failure. On the one hand, it can be attempted to directly employ monovalent alkali metals as independent anode, which is promising to bring higher theoretical capacity and energy density. On the other hand, constructing an artificial SEI functional layer on the electrode surface or developing heterostructure metals or alloys can also enhance the electrochemical performance, enhance the reaction kinetics, and improve the transport of reactive ions to a certain extent. Furthermore, to facilitate comparison and better understanding, Table 2 lists the configurations and electrochemical performance of DIBs based on various metallic anode materials.

**Table 2:** Configurations and electrochemical performance of DIBs based on various metallic anode materials

Type	Anode//Cathode Configuration	Electrolyte systems	SDC (mAh g^−1^)	Cyclic performance	Energy density (Wh kg^−1^)	Refs.
Alloy	Al//Natural graphite	4 M LiPF_6_-EMC- 2wt% VC	104 at 2C	88% after 200 cycles at 2C	222 (based on anode and cathode)	[56]
	Al//EG	7.5 m LiFSI-EC-DMC-1:1	94 at 2C	96.8% after 500 cycles at 2C	180 (based on anode, cathode and electrolyte)	[147]
	nAl@C// Graphite	4 M LiPF_6_-EMC-5wt% VC	105 at 2C	94.6% after 1000 cycles at 2C	148 (based on anode and cathode)	[151]
	pAl/C//NG	4 M LiPF_6_-EMC-5wt% VC	104 at 2C	89.4% after 1000 cycles at 2C	232 (based on anode and cathode)	[152]
	ACNI/Al//EG	4 M LiPF_6_-EMC-5wt% VC	115 at 2C	94% after 1000 cycles at 2C	/	[153]
	Sn//Graphite	1 M NaPF_6_-EC-DMC-EMC	74 at 2C	94% after 400 cycles at 2C	144 (based on the cathode)	[57]
	Sn//EG	1 M KPF_6_-EC-DMC-EMC	66 at 0.5C	93% after 300 cycles at 2C	155 (based on the cathode)	[58]
	Sn//NG	0.8 M Ca(PF_6_)_2_-EC-PC-EMC-DMC	72 at 1C	95% after 350 cycles at 1C	/	[59]
	3 Mg/Mg_2_Sn// x-PVCz	2 M Mg(TFSI)_2_-ACN	133 at 1C	97.3% after 1000 cycles at 10C	394 (based on the cathode)	[160]
	Sn//EG	1 M KPF_6_-EC-EMC-DMC-PC	106 at 5C	100% after 500 cycles at 5C	/	[161]
	HPSbCNFs// Graphite	1 M KPF_6_-EC-DMC	440 at 2C	61.5% after 1440 cycles at 5C	/	[162]
	Ge/CNFs// Graphite	1 M LiPF_6_-EC-DMC	281 at 2.5C	74.2% after 500 cycles at 25C	/	[163]
	Si/Cu-Ni//EG	4 M LiPF_6_-EMC-2wt% VC	96 at 2C	97% after 2000 cycles at 10C	/	[169]
	RP/CNT@GO// Graphite	1 M NaPF_6_-EC-DMC-EMC	373 at 2C	76% after 140 cycles at 5C	176 (based on anode and cathode)	[170]
Conversion	MoSSeNSs@NC/hC-NC//EG	3 M NaPF_6_-EC-DMC-EMC	204 at 1C	91% after 2000 cycles at 10C	101 (based on anode and cathode)	[179]
	NbSSe/NC//EG	3 M NaPF_6_-EC-DMC- 7% FEC	62 at 0.5C	89.1% after 1000 cycles at 10C	230.6 (based on anode and cathode)	[180]
	α-Fe_2_O_3_//KS6	1 M Li/NaTFSI-Pyr_14_TFSI	227 at 2C	100% after 520 cycles at 5C	/	[181]
	MoS_2_/C//EG	1 M NaPF_6_-EC-EMC-DMC	65 at 2C	85% after 200 cycles at 2C	/	[182]
	MoSe_2-x_/ ZnSe@C//EG	1 M NaPF_6_-EC-EMC-DMC	209 at 0.5C	53% after 1000 cycles at 10C	131 (based on anode and cathode)	[183]
Deposition	Li//Graphite	3 M LiPF_6_-EMC	95 at 0.1C	86.4% after 2000 cycles at 12C	/	[188]
	Zn//EG	1 M Zn(TFSI)_2_-Pyr_14_TFSI	57 at 2C	86% after 500 cycles at 10C	/	[189]
	SEI-modified Li //Graphite	4 M LiPF_6_-EMC	95.2 at 10C	89.6% after 500 cycles at 10C	/	[193]
	Li// Graphite	4 M LiPF_6_-EMC-LiNO_3_	91 at 2C	97% after 300 cycles at 2C	243 (based on the and cathode)	[194]

## Organic Materials

Organic materials are completely free of metal elements and consist of abundant and sustainable elements such as C, H, O, N, and S, which are low cost, environment-friendly, and renewable [196–198]. Moreover, the electrochemical performance of redox-active organic materials can be tailored by grafting specific molecular frameworks or functional groups, thus exhibiting structural designability [199–201]. Specifically, organic materials generally store reactive ions based on an adsorption/desorption reaction mechanism without involving reactions such as ion insertion, alloying, and conversion, which do not alter the structure and valence bonds of organic materials, leading to low reaction energy barriers, promising rate capability, and superior cyclic stability [202, 203]. What is more, owing to the outstanding chemical versatility exhibited by organic materials, which can bring design flexibility for optimizing discharge capacity, energy/power density, operating voltage, physicochemical properties, and synthesis process. They are gradually becoming an attractive candidate electrode material for green and sustainable electrochemical energy storage devices and represent a great potential for application in DIBs. However, the development of organic electrode materials in DIBs is still in the primary stage, and there is a lack of in-depth research on their energy storage mechanisms. In order to help researchers better understand the advancement of organic materials in DIBs, a systematic exposition will be conducted from the following aspects.

### Polycyclic Aromatic Hydrocarbon

**Fig. 10:**
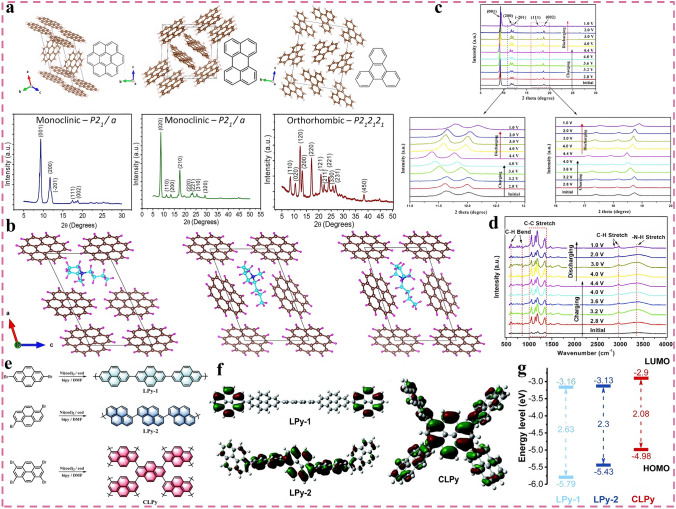
Application of PAHs anode in DIBs. **a** Crystal structures of the studied PAHs with their XRD patterns and space groups, reproduced with permission [204]. Copyright 2018, American Chemical Society. **b** Optimized structures of the different orientations of BMP^+^-intercalated coronene anode, reproduced with permission [207]. Copyright 2020, Wiley. **c** XRD and **d** FT-IR spectra of the coronene anode in various states before cycling and through the second charge–discharge cycle, reproduced with permission [208]. Copyright 2020, Wiley. **e** Preparation routes of polypyrenes. **f** HOMO diagram and **g** the energy levels of LPy-1, LPy-2 and CLPy, reproduced with permission [209]. Copyright 2020, Royal Society of Chemistry

Polycyclic aromatic hydrocarbons (PAHs) refer to aromatic hydrocarbons containing two or more condensed benzene rings, which are similar to graphite in terms of large conjugated π-bonds, are graphene nanofragments with a size in the range of 1–5 nm consisting of *sp*^2^ hybridization of carbon atoms [204, 205]. Figure 10a gives a list of the crystal structures and corresponding XRD models of several PAHs with diverse numbers of benzene rings in different axial directions. It can be observed that PAHs are structurally ordered and stacked by van der Waals forces into a π–π conjugated system with a huge expansion and a high degree of crystallinity and can accommodate additional electrons that escape on the lowest unoccupied molecular orbital (LUMO) or the highest occupied molecular orbital (HOMO), which is greatly conducive to the storage of reactive ions [69, 204, 205]. Theory combined with experiments indicates that, on the one hand, the smaller the molecular conjugate size of PAHs, i.e., the less the number of benzene rings, can provide higher SDCs, which is attributed to the fact that small-sized PAHs share more edge-active sites, this is favorable for the adsorption of more reactive ions. On the other hand, small-sized PAHs also exhibit a lower charge/discharge plateau, which is due to their larger LUMO–HOMO gap, resulting in a reduced electron density and thus a smaller binding energy with active ions [69, 206–208]. However, this also leads to a worse cyclic stability of small-sized PAHs because of their higher solubility in electrolyte, which causes a drastic degradation or even failure of the battery performance [196, 200, 204].

As one of the typical PAHs, coronene shows satisfied electrochemical performance and has been widely reported to be utilized as a cathode in DIBs for the storage of anion [206]. Interestingly, coronene also exhibits an efficient cation storage capability, but the related work has rarely been reported. Pathak et al. reported the application of coronene as an anode in ionic liquid-based DIBs systems with BMPCl-AlCl_3_ as electrolyte and compared the storage of BMP^+^ in coronene and graphite in detail. The DFT calculations demonstrate that the binding energy of BMP^+^ with coronene anode is 1.71 eV, which is lower than that of graphite (2.36 eV), proving that coronene exhibits better thermodynamics for the faster reversibility of the reactive ions insertion/desertion into/from coronene [207]. Besides, the volume expansion of BMP^+^ when totally intercalated into the crystal structure of coronene is 53%, which is significantly lower than that of graphite (148%), implying that coronene shows better structural stability as well as more superior cation storage capability than graphite. Specifically, to deeply probe the optimal binding sites of BMP^+^ in coronene anode, three possible intercalation orientations (S1, S2, and S3 orientations) of butyl and methyl chains in the BMP^+^ structure at different spatial sites on the coronene unit cell are simulated by AIMD as depicted in Fig. 10b. The reaction energy barriers in these three orientations are calculated separately, and it is discovered that the butyl and radical occupy the c-direction and negative a-direction of the coronene unit cell, respectively, with the lowest energy barrier (S1, 0 eV), which are remarkably lower than the barrier that occupies the negative a-direction and c-direction (S3, 1.24 eV) as well as the barriers occupying the negative a-direction and positive b-direction (S2, 4.96 eV). This is attributed to the fact that BMP^+^ is located in the vacancies and edge sites of coronene on the S1 orientation, which implies a more favorable reaction kinetic behavior and is highly conducive to the storage of BMP^+^, whereas on the S2 and S3 orientations, the BMP^+^ and coronene suffer from severe molecular repulsion and exhibit a high degree of instability. The crystal structure evolution of the coronene anode during charging and discharging is further explored by XRD and FT-IR [208]. At the pristine state, the XRD in Fig. 10c displays the typical (001), (200), (201), (111), and (002) diffraction peaks, while the characteristic peaks between 500–1000 cm^−1^ in the FT-IR belong to the C–H out-of-plane bending vibration of coronene, and the peaks between 1000–1500 cm^−1^ are attributed to the C–C stretching vibration of the aromatic ring (Fig. 10 d). During the charge process, the XRD peaks are gradually shifted to the left, accompanied by a decrease in peak intensity. Meanwhile, the C-H and N–H characteristic peaks appear at 2880 and 3400 cm^−1^, indicating that the cations are intercalated into the crystal structure of coronene and participate in the electrochemical reaction. In the subsequent discharge process, all the peaks return to the initial position with recovered intensity, and the characteristic peaks belonging to coronene in Fig. 10d basically remain unchanged throughout the whole process, which indicates that the reaction is highly reversible as well as the excellent structural stability of coronene. For further improving the electrochemical performance of PAHs, Ji et al. have synthesized a class of polypyrenes with different connection modes and electronic structures and deeply investigated their structure-performance correlations [209]. In particular, three types of polypyrenes (LPy-1, LPy-2, and CLPy) are synthesized by nickel-catalyzed Yamamoto coupling reactions as shown in Fig. 10e, in which LPy-1 and LPy-2 are in the linear linkage mode, while CLPy is in the reciprocal cross-linking mode. The N_2_ adsorption/desorption curves indicate that CLPy possesses the relatively highest BET specific surface area and the largest percentage of mesoporous structure, which effectively enhances the electrode–electrolyte contact area and facilitates the ion/electron transport, thereby expecting to bring about high redox activity and rate capability. The HOMO orbital distributions and electronic structures of LPy-1, LPy-2, and CLPy are further probed by DFT calculations and Frontier Molecular Orbital Theory (FMOT) to reveal the structural dependence of polypyrenes. The results are presented in Fig. 10f, in which the HOMO orbitals of LPy-1 are distributed on two separated and non-overlapping perylene units, and the HOMO orbitals of LPy-2 are distributed on three consecutive and overlapping perylene units, whereas the HOMO orbitals of CLPy are further extended uniformly along all the perylene units. Specifically, a higher HOMO energy level means stronger electron-donating capability, while a narrower bandgap suggests higher electrical conductivity. This also implies that CLPy exhibits the best active ion storage performance, and the corresponding proof-of- concept DIBs with CLPy anode display the highest SDC of 180 mAh g^−1^ at 50 mA g^−1^ and an ultra-long cycle life of 38,000 cycles at 3000 mA g^−1^ with a CR of 96.4%.

### N-Type Organic Materials

**Fig. 11:**
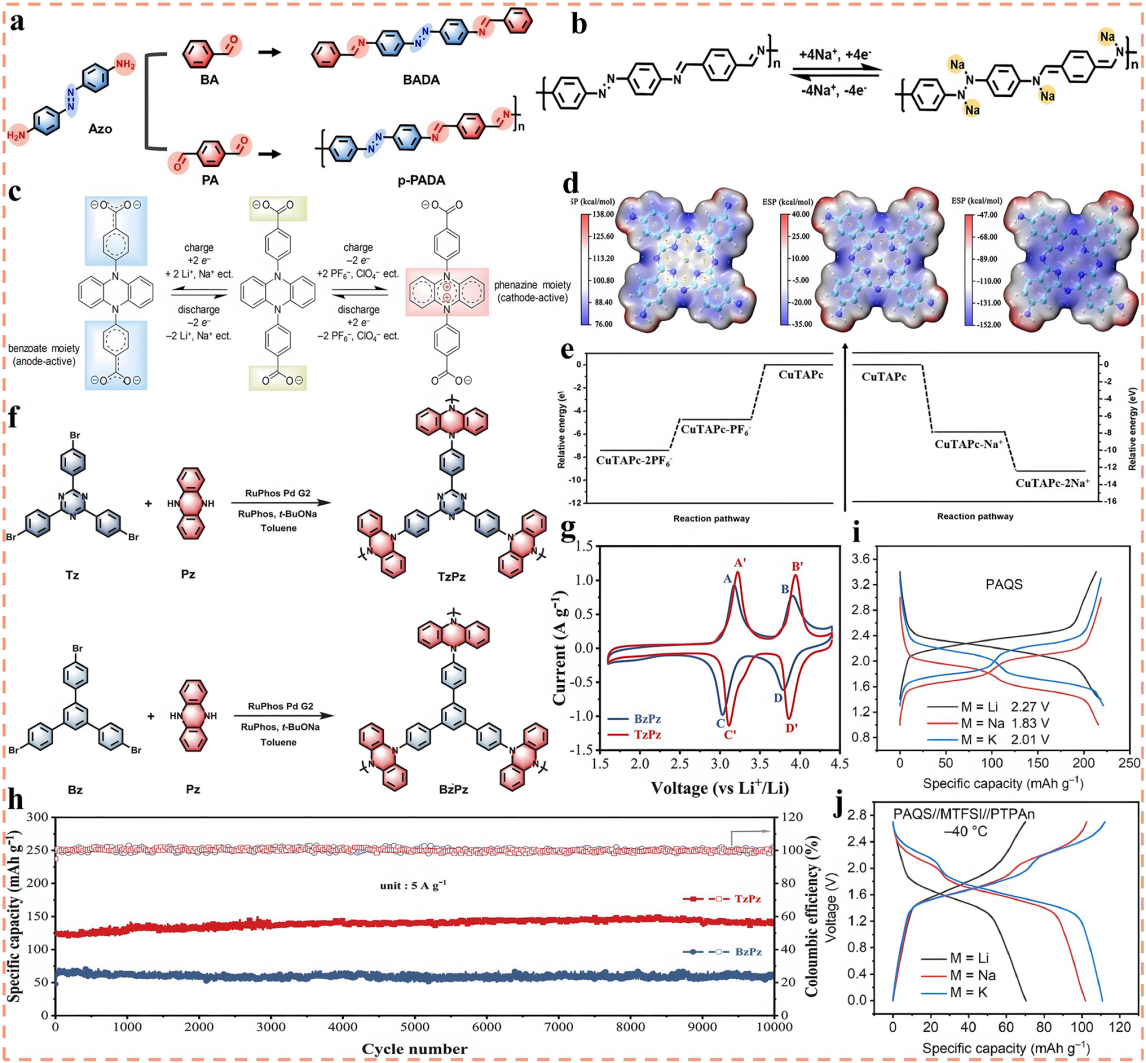
Application and modification strategies of N-Type organic materials in DIBs. **a** Synthetic routes of BADA and p-PADA and **b** structural evolution of p-PADA during redox sodization, reproduced with permission [213]. Copyright 2023, Wiley. **c** Redox mechanism of PZDB, reproduced with permission [214]. Copyright 2019, Wiley. **d** MESP of CuTAPc^2+^, CuTAPc and CuTAPc^2−^ and **e** binding energies between CuTAPc and PF_6_^−^ or Na^+^, reproduced with permission [215]. Copyright 2021, Elsevier. **f** Synthetic routes of TzPz and BzPz. **g** CV curves at 1 mV s^−1^ and **h** cyclic performance at 5 A g^−1^, reproduced with permission [216]. Copyright 2021, Wiley. **i** Typical charge–discharge curves at 100 mA g^−1^ and **j** typical CV curves at 0.1 mV s^−1^ of PAQS, reproduced with permission [216]. Copyright 2023, Elsevier

Generally speaking, the redox properties of active organic compounds depend on the redox groups, that is, the functional groups in the molecules that confers redox activity [210–212]. N-type organics usually behave as functional groups with redox activity in the molecule accept electrons during electrochemical reaction, reduce from the neutral state to form a negatively charged molecular state, and participate in the reversible redox reaction with positively charged reactive cations. N-type organics can be roughly divided into N-functional organics, S-functional organics, and carbonyl compounds (described separately in the next section). Zhang et al. employed p-phenylenedicarboxaldehyde and 4,4'-diazoaniline (Azo) to conduct the Schiff base reaction as illustrated in Fig. 11a and synthesized two conjugated Azo compounds for the storage of Na^+^, one of which is the small molecule compound benzaldehyde-4,4’-diazoanilidine (BADA), and the other is the polymer terephthalaldehyde-4,4’-diazoanilidine (p-PADA) with linear and extended repeating units [213]. The HOMO–LUMO energy levels of the two compounds are calculated by FMOT, where PADA shares a narrower energy gap, implying higher conductivity, which is due to the extended π-conjugated structure of p-PADA promoting the electron delocalization. In addition, p-PADA displays a more stable structure than BADA and is virtually insoluble in electrolyte. Therefore, p-PADA exhibits good rate capability and cyclic stability. It is further demonstrated via FT-IR that p-PADA shows a reversible Na^+^ storage mechanism as illustrated in Fig. 11b, where the N = N and C = N bonds are converted to N–N^−^ and C-N^−^ and reacted with Na^+^ during sodiumation, and then revert to N = N and C = N bonds during subsequent de-sodiumation.

Surprisingly, a type of N-functional organic compound 4,4′-(phenazine-5,10-diyl)dibenzoate anion (PZDB) based on the phenazine skeleton exhibits bipolarity and can be utilized as both cathode and anode for the storage of anions and cations [214]. As revealed in Fig. 11c, both the phenazine moiety and benzoate moiety can be applied as redox-active centers for the storage of reactive ions. The FMOT and aromaticity analyses indicate that the phenazine and benzoate moieties display highly localized redox activity, which notably strengthens the structural stability of PZDB. The constructed all-organic symmetric DIBs display a SDC of 53 mAh g^−1^ and an ED of 127 Wh kg^−1^ (based on the total mass of cathode and anode) at 1C with a CR of 97% after 200 cycles. Except for PZDB, phthalocyanine, which combines both donor and acceptor structures, can also be regarded as a bipolar reactive organic molecule that could provide or accept electrons in redox reactions. Moreover, the cavities within the structure of phthalocyanine could associate with a given metal ion to form a metal phthalocyanine, which contributes to the enhancement of redox activity and hence improves the electrochemical performance. On this basis, the bipolar metal phthalocyanine complex-CuTAPc was constructed and acts as the cathode and anode of sodium-based DIBs [215]. The bipolar reactivity of CuTAPc was deeply analyzed by molecular electrostatic potential (ESP), as depicted in Fig. 11d, the ESP distributions of oxidized (CuTAPc^2+^), neutral, and reduced states (CuTAPc^2−^) of CuTAPc, with the blue and red regions representing negative and positive MESP values, respectively. The results demonstrate that the -C-N group exhibits a remarkably positive MESP value for CuTAPc^2+^, yet a remarkably negative MESP value for CuTAPc^2−^. Accordingly, during the charge process, CuTAPc as the cathode can be oxidized to CuTAPc^2+^ and combined with anions, while CuTAPc as the anode can be reduced to CuTAPc^2−^ and bound to cations. The variation of the binding energy for CuTAPc during the electrochemical reaction was further analyzed by DFT calculations. A higher binding energy (−7.44 and −12.47 eV) was exhibited when CuTAPc was reacted with an anion (PF_6_^−^) and a cation (Na^+^), respectively, suggesting that CuTAPc as a bipolar electrode is favorable for storing anions and cations (Fig. 11e). In particular, CuTAPc demonstrates stronger binding energies when interacting with the second anion and cation, indicating that it is feasible for CuTAPc to perform a four-electron reaction in combination with two anions/cations. The designed full batteries deliver a high SDC of 195.7 mAh g^−1^ at 50 mA g^−1^, a median discharge voltage of 3.2 V, and an ED of 324 Wh kg^−1^ (based on the mass of anode) at a PD of 7481 W kg^−1^. However, the DIBs displayed an underwhelming cyclic performance, with a CR of only 58.2% after 2500 cycles at 1 A g^−1^. In order to further promote the cycle life of N-type organic anode-based DIBs, a class of conjugated microporous polymer materials (TzPz) with D-A structure were designed by Buchwald-Hartwig polycondensation reaction based on the donor–acceptor structural design principle [216]. Compared with BzPz without D-A structure, TzPz exhibits high-level redox reversibility and theoretical capacity. Specifically, the D-A structure increases the conjugation degree of TzPz but decreases the HOMO–LUMO energy gap, which both enhances the structural stability and facilitates the transport of electrons along the polymer chain, and it is expected to bring about excellent cyclic and rate performance. Furthermore, TzPz possesses a higher percentage of micropores and exhibits a larger specific surface area, which exposes more active sites and enhances the transport and storage of active ions. This was evidenced by the cyclic voltammetry (CV) curves in Fig. 11g, where TzPz displays sharper and more symmetrical redox peaks with higher response peak current densities, implying the better redox reversibility, and the total area of the CV curves for TzPz is larger than that of BzPz, which indicates a higher discharge capacity. As a result, the TzPz electrode-based DIBs deliver a higher SDC of 192 mAh g^−1^ at 0.2 A g^−1^ and can be cycled stably for more than 10,000 cycles at 5 A g^−1^ (Fig. 11h).

The application of S-functional organic anode materials in DIBs is relatively rare, and the current research is mainly focused on poly(anthraquinonyl sulfide) (PAQS) materials. As Song et al. systematically analyzed and compared the redox thermodynamics and kinetics behavior of PAQS electrodes in Li^+^/Na^+^/K^+^-based systems [216]. Figure 11i illustrates the GCD curves of these three systems at 100 mA g^−1^, and all SDCs are close to the theoretical capacity of PAQS (225 mAh g^−1^), indicating that the electrochemical reactions of PAQS with metal cations are sufficiently performed. For the Li^+^-based system, only one pair of charge and discharge platforms appear with the highest corresponding charge and discharge potentials, whereas for Na^+^ and K^+^, not only present two pairs of platforms, but also the voltage plateaus are much smoother with lower potentials. The average charge and discharge potentials of the three systems are further quantified, and the mean values of them is defined as the average redox or thermodynamic potential as well as the electrochemical polarization. The consequences indicate that the average redox potential of Li^+^/ Na^+^/K^+^ decreases sequentially, whereas a larger average redox potential implies a greater reaction energy barrier, polarization effect and a higher negative potential, which suggests that the redox kinetics improves with increasing ionic radius in this system. Therefore, when PAQS was applied as anode for DIBs, the lower the average redox potential, the higher the output voltage and the weaker the polarization, along with the high redox kinetics are expected to deliver a superior electrochemical performance. As demonstrated in Fig. 11j, the discharge voltage and SDC of the Li^+^/Na^+^/K^+^-based system increased sequentially, the charge and discharge platforms are flatter sequentially, and the gaps between the platforms are reduced in turn, i.e., the polarization is weakened sequentially. Xiao et al. has investigated a class of DIBs based on PAQS anode and pure EMImTFSI ionic liquid electrolyte [217]. It should be noted that the PAQS were pre-oxidized with H_2_SO_4_ acidification to activate the sulfone group of PAQS and enhance the adsorption capability of the active-site S = O bond, which remarkably improves the cation-storage ability and conductivity with respect to the untreated PAQS. The designed full batteries exhibit a SDC of 128.3 mAh g^−1^ at 2C with superior rate capability. However, the size of cations in the ionic liquid electrolyte is notably larger than that of metal cations, resulting in irreversible structural destruction of PAQS during cycling, which in turn leads to a sharp capacity decay and battery failure. In this context, a type of PAQS with an accordion-like shape is synthesized by high-temperature polycondensation, its special architecture promotes both ion and electron transport and suppresses the volume expansion caused by the insertion of large-sized ionic liquid cations, which consequently improves the rate and cyclic stability [217]. The corresponding DIBs not only offer a SDC of 111.8 mAh g^−1^ at 10C, but also can be cycled stably for 1000 cycles at an ultra-high rate of 120C without capacity degradation.

### Carbonyl-Containing Organic Materials

**Fig. 12:**
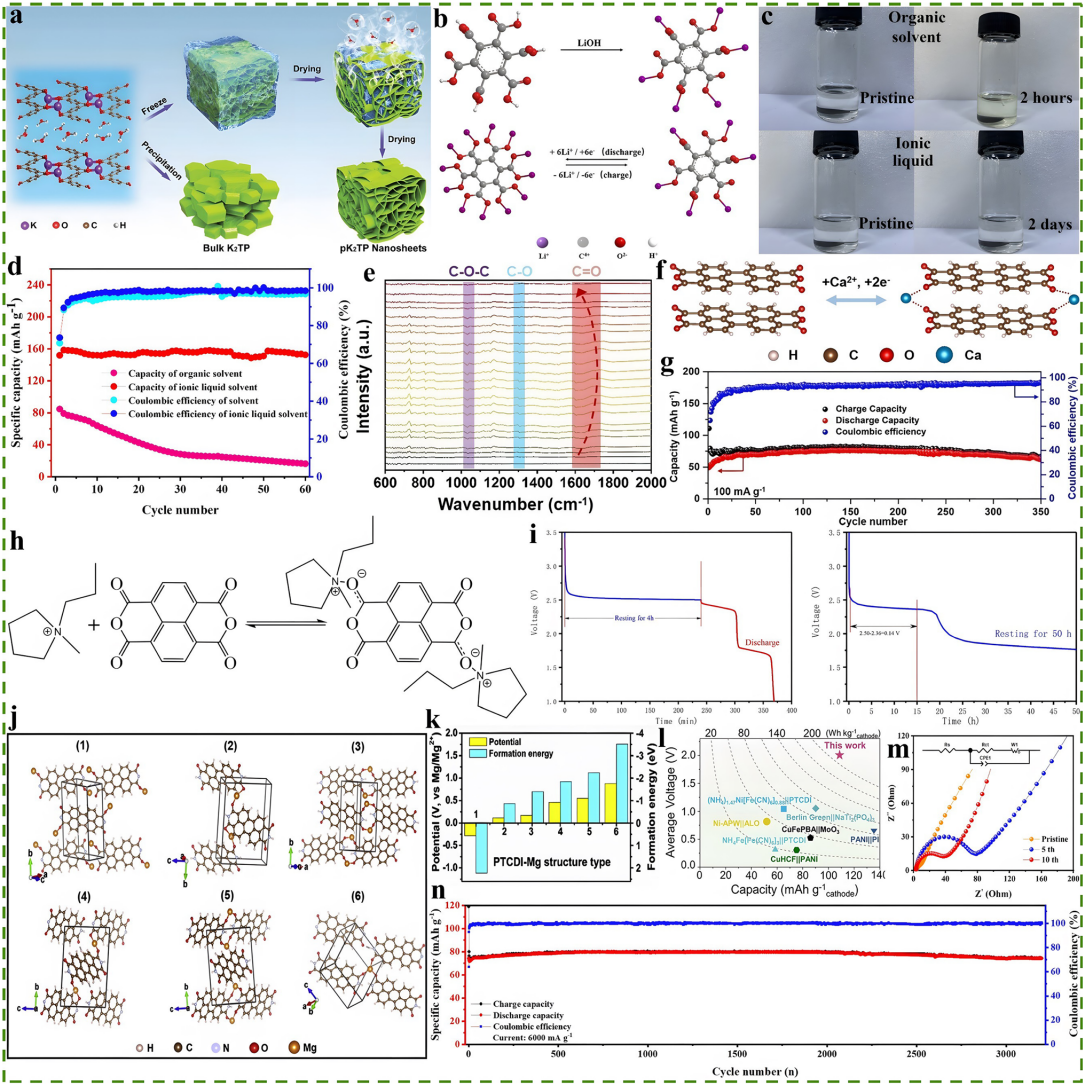
Application of carbonyl compounds in DIBs. **a** Schematic illustration of the synthesis routes for pK_2_TP nanosheets and bulk K_2_TP, reproduced with permission [221]. Copyright 2020, Wiley. **b** Scheme of the synthesis process of Li_6_C_12_O_12_ and the storage mechanism of Li_6_C_12_O_12_ electrode for LIBs, reproduced with permission [222]. Copyright 2023, Elsevier. **c** Solubility test of PTCDA in ionic liquid and organic solvent and **d** cycle performance based on organic and ionic liquid solvent at 100 mA g^–1^, reproduced with permission [223]. Copyright 2021, American Chemical Society. **e** In situ FT-IR results for PTCDA anode. **f** Proposed reaction mechanism of PTCDA with Ca^2+^ and **g** long cycling test of PTCDA||graphite Ca-DIB at 100 mA g^−1^, reproduced with permission [224]. Copyright 2022, Wiley. **h** Reaction mechanism of NDCTA and Pyr_14_^+^ and **i** voltage–time curve of battery during standing for 4 and 50 h, reproduced with permission [225]. Copyright 2021, Elsevier. **j** Unit cells of different PTCDI-Mg intercalation structures and **k** the corresponding calculated formation energy and potential, reproduced with permission [61]. Copyright 2020, Elsevier. **l** A comparison of graphite||PTCDI battery with reported NH_4_^+^-based full battery systems, reproduced with permission [229]. Copyright 2022, Wiley. **m** EIS and its fitting diagram and **n** the long-term cycling performance under 60C of PNTO//KS_6_-DIB, reproduced with permission [230]. Copyright 2022, American Chemical Society

Carbonyl organic materials, due to their inherent advantages such as high theoretical capacity, flexible and diverse structural designability, stable structure, fast reaction kinetics, and environmental friendliness, are currently the most widely researched anode materials for DIBs, demonstrating enormous potential for innovative electrochemical energy storage applications [218–220]. The storage mechanism of carbonyl compounds can be summarized as the reversible formation of the free radical anion C-O^−^ from C = O through enolization reaction and adsorption of reactive cations to reach the neutral state. The carbonyl compounds commonly employed as anode for DIBs are mainly carboxylates, anhydrides, ketones, imides, and their derived polymers. For example, 3D porous dipotassium terephthalate nanosheets (pK_2_TP) have been synthesized via acid–base reaction and freeze-drying method by Yu et al. [221]. In this process, the freeze-drying step is crucial because ice crystals are generated as templates in the molecular interlayers of K_2_TP, and the sublimation of ice crystals into water vapor during the subsequent drying process led to the formation of 3D porous nanosheets of K_2_TP that are cross-linked with each other as shown in Fig. 12a. Compared to the bulk aggregates formed without freeze-drying, pK_2_TP exhibits decent conductivity, reaction kinetics, surface pseudocapacitance, and structural stability. When pK_2_TP is used as anode of DIBs with the EG cathode and 1 M KPF_6_ EC-EMC-DMC (4:3:2, v/v/v) electrolyte, the constructed pK_2_TP//EG-DIBs exhibit a SDC of 68 mAh g^−1^ at 2C, and can be cycled for 2000 cycles at 5C without capacity degradation with a median discharge voltage stabilized around 2 V. To further improve the ability of carboxylate carbonyl compounds to store reactive ions, hexalithium salt of mellitic acid (Li_6_C_12_O_12_) was synthesized by one-step green synthesis of H_6_C_12_O_12_ and LiOH in ethanol solution, and Li_6_C_12_O_12_ is expected to perform a reversible six-electron reaction with Li^+^ (Fig. 12b), which brings about a high SDC [222]. Firstly, it is verified by assembling Li_6_C_12_O_12_//Li half-cell, which shares a high SDC of 527 mAh g^−1^ at 0.21 A g^−1^ and decreases to 455 mAh g^−1^ after 10 cycles owing to the decomposition of electrolyte and partially irreversible electrode reaction. The capacity gradually rises and stabilizes at around 730 mAh g^−1^ during the subsequent cycles, which is attributed to the adequate penetration of electrolyte, the formation of a stable SEI as well as the sufficient activation of the Li_6_C_12_O_12_ electrode. This strongly proves that Li_6_C_12_O_12_ indeed features excellent capability for Li^+^ storage. As a result, it displays an outstanding electrochemical performance when used as anode for DIBs, and the assembled full batteries provide a SDC of 80 mAh g^−1^ and an ED of 284 Wh kg^−1^ (based on the mass of anode) at 110 mA g^−1^ with a CR of 91% after 160 cycles. However, carboxylate carbonyl compounds are characterized by poor stability, insufficient reaction and low conductivity, and there are still no effective measures that can address the corresponding shortcomings.

To further improve the discharge capacity and cycle life of DIBs, perylene-3,4,9,10-tetracarboxylic acid dianhydride (PTCDA), an anhydride-based carbonyl compound with multiple carbonyl functional groups, was employed as an anode for more efficient storage of active cations [223]. Especially, organic small molecules are easily soluble in organic carbonate solvents due to the principle of similarity solubility, which will result in unsatisfactory electrochemical performance. Therefore, pure ionic liquid of Pyr_14_TFSI without solvent molecules is used as solvent and the solubilization behavior of the PTCDA electrode in ionic liquid and carbonate solvent (EC-EMC v/v, 1:1) is comparatively analyzed. Compared with the organic solvent, the ionic liquid electrolyte is completely composed of anions and cations without solvent molecules, it is thermally stable and non-flammable, features a wide electrochemical window, avoids solvent co-insertion, and exhibits high compatibility with PTCDA anode. It can be observed from Fig. 12c that the PTCDA electrode can exist stably in the ionic liquid without dissolution and can express better electrochemical performance over a wider voltage range. Correspondingly, the full DIBs based on the PTCDA anode and ionic liquid electrolyte exhibit a high SDC of 153 mAh g^−1^ with a CE of 98.5% at 2C, as the capacity does not decay after 60 cycles, and the relevant GCD curves at different cycles basically overlap and present smooth and clear charge and discharge platforms (Fig. 12d). In contrast, PTCDA shows poor stability in carbonate solvents and a severe dissolution phenomenon, with the color of the solvent changing from colorless to yellow. The corresponding DIBs based on organic carbonate electrolyte exhibit a SDC of only 82 mAh g^−1^ at 2C and suffer a serious decay during the subsequent cycles. Apart from the adoption of ionic liquids, increasing the concentration of electrolyte can also inhibit the dissolution of PTCDA and deliver favorable electrochemical performance. Li et al. constructed the DIBs based on the PTCDA anode, EG cathode, and a kind of calcium-based electrolyte with a high concentration of 3.5 m Ca(FSI)_2_ in EC-PC-EMC-DMC (2:2:3:3, v/v/v/v) [224]. Due to the utilization of concentrated electrolyte, the free molecules in solvent are significantly reduced and present as contact ion pairs and aggregates, which greatly enhances the structural stability of electrodes and broadens the electrochemical window. As illustrated in Fig. 12e, the storage mechanism of Ca^2+^ in PTCDA was explored by an in situ FT-IR technique. During the charge process, the stretching pattern of C=O characteristic peaks gradually presents positive and negative transmittance values at 1700 and 1605 cm^−1^, respectively, which implies that C=O is reduced. Meanwhile, both the C–O at 1306 cm^−1^ and C–O–C at 1029 cm^−1^ show negative transmittance values, which further proves that –C=O is reduced to –C–O^−^ and participate in the electrochemical reaction with Ca^2+^ (Fig. 12f). During the subsequent discharge process, the C=O and C–O characteristic peaks recover to the initial state, indicating the reversibility of the reaction. Accordingly, the proof-of-concept Ca-DIB provides a SDC of 75.4 mAh g^−1^ at 1C with a CR of 84.7% after 350 cycles (Fig. 12g). Similarly, naphthalene tetracarboxylic dianhydride (NTCDA) also exhibits decent electrochemical activity and can be used to store large-sized non-metallic cations. When utilized in the pure Pyr_14_TFSI ionic liquid electrolyte system with the NG cathode, the metal-free DIB (NGB) constructed is able to express a low self-discharge rate. Specifically, the C=O active site of the NTCDA anode can undergo a reversible electrochemical reaction with Pyr_14_^+^ as shown in Fig. 12h, and shares a SDC of 114 mAh g^−1^ at 0.1C [225]. When the NGB was shelved for 15 h, the voltage drops by only 0.14 V (Fig. 12i), and the voltage attenuation rate is calculated to be as low as 0.0095 V s^−1^, which indicates that the binding capability of NTCDA with Pyr_14_^+^ is so robust that it is difficult for Pyr_14_^+^ to spontaneously disengage. Furthermore, a kind of ketone carbonyl compound 5,7,12,14-pentaphenyltetraketone (PCT) can reversibly store Pyr_14_^+^ and exhibit a relatively higher SDC [226]. Accordingly, the designed metal-free DIBs display a high SDC of 164.5 mAh g^−1^ at 0.1C with a CR of 92.2% after 100 cycles at 5C. Nevertheless, the DIBs show an unsatisfactory self-discharge rate of 4.68% h^−1^ after resting for 5 h. Further consideration needs to be given in the future on how to effectively reduce the self-discharge effect of ketone-based organic DIBs.

Imide carbonyl compounds are currently the most suitable class of organic anode active materials for DIBs due to their relatively more stable structure and higher reactivity. Lei et al. reported a type of magnesium-based DIBs based on the insoluble small molecule 3,4,9,10-perylenetetracarboxylic acid diimide (PTCDI) anode, which exhibit decent cyclic performance [61]. Firstly, the insertion mechanism of Mg^2+^ into PTCDI was explored by first-principles calculation and the reasonable PTCDI-Mg coordination mode and its crystal structure are predicted. Figure 12j presents the six possible PTCDI-Mg structures and their corresponding formation energy values and potential values. When two Mg^2+^ are inserted into the crystal structure of PTCDI, three possible coordination modes are observed: connection between C = O and Mg^2+^ contained dangling binding (Structure 1), shoulder-to-shoulder into a chain (Structure 2) and head-to-head into a ring (Structure 3). However, the PTCDI-Mg structure in these three cases displays a large formation energy and a low potential, implying that the structure of PTCDI was quite unstable when two Mg^2+^ are inserted as shown in Fig. 12k. On the contrary, when one Mg^2+^ is inserted, it displays low formation energies and high potential values, indicating that this is feasible. In particular, the electrostatic repulsion exhibited when a single Mg^2+^ with threefold coordination of adjacent carbonyl is notably lower than that of structures 4 and 5 with two coordination into a chain or a ring, which implies that structure 6 possesses the lowest formation energy and the highest potential value and is the most likely coordination structure. The Mg-DIBs constructed on this basis deliver a SDC of 56.1 mAh g^−1^ at 5C with a CR of 95.7% after 500 cycles. Beyond the promising ability to store Mg^2+^, PTCDI is also capable of efficiently storing K^+^, Ca^2+^, and NH_4_^+^ with outstanding electrochemical performance [227–229]. For example, as PTCDI is applied to the system of NH_4_^+^-based aqueous DIBs, the full batteries boast a high operating voltage of up to 2.75 V, a SDC of 107.9 mAh g^−1^, and an ED of 200 Wh kg^−1^ (based on the mass of cathode), which is significantly higher than that of the currently reported aqueous system of ammonium-ion batteries (Fig. 12l) [229]. To further optimize the discharge capacity and cyclic stability of imides, a feasible approach is to synthesize polymer-polyimides (PI) based on imide repeating units. Wu et al. designed a type of PI active material (PNTO) for Na^+^ storage via high temperature polycondensation, which ingeniously incorporates the urea ligand during the polycondensation process, thereby introducing an additional reactive functional group of C = O into the backbone of PNTO, this strategy both enhances the structural stability and achieves higher theoretical capacity [230]. The proof-of-concept full batteries (PNTO//KS_6_-DIBs) are assembled for electrochemical performance measurements. Electrochemical impedance spectroscopy (EIS) in Fig. 12m shows that the charge transfer impedance Rct values show a tendency to increase and then decrease during cycling, which indicates that the PNTO electrode is favorable for the transport of Na^+^. Consequently, the PNTO//KS_6_-DIBs achieve an ultra-high SDC of up to 227.6 mAh g^−1^ at 1C and can be cycled stably for 250 cycles without degradation. Furthermore, the DIBs display superior rate capability and cyclic stability at high rate, with a SDC of 74.2 mAh g^−1^ even at an ultra-high rate of 60C as well as a 100% CR after 3200 cycles (Fig. 12n). Even in aqueous electrolyte systems, PI also features great potential for application. Zhu et al. developed a kind of aqueous all-organic Mg-based DIBs based on the PI anode and a polyaniline cathode [231]. The full batteries offer an ED of 29.1 Wh kg^−1^ (based on the total mass of the electrode materials and electrolyte) at 5C, and are stable for 6000 cycles at a high rate of 50C with a CR of 86.2%, implying the fast reaction kinematics.

In conclusion, organic materials, especially carbonyl compounds, are increasingly emerging as a promising category of anode materials for green and sustainable DIBs due to their wide sources, adjustable structure, and superior performance. However, the poor conductivity, severe solubility in the electrolyte and high operating potential of organic materials limit their further development. The synthesis of organic electrode materials with high capacity, high conductivity and low solubility by molecular engineering strategy is the focus of current research. In addition, the construction of all-organic DIBs can also significantly reduce the cost and bring a great environmental advantage, which is expected to realize large-scale green and sustainable electrochemical energy storage applications. Besides, for the convenience of comparison and better understanding by researchers, the configuration and electrochemical performance of DIBs based on various organic anode materials are listed in Table 3.

**Table 3:** Configurations and electrochemical performance of DIBs based on various organic anode materials

Type	Anode//Cathode Configuration	Electrolyte systems	SDC (mAh g^−1^)	Cyclic performance	Energy density (Wh kg^−1^)	Refs.
PAHs	Coronene// Graphite	Pyr_14_TFSI	73.3 at 3C	93% after 100 cycles at 3C	102 (based on the anode)	[208]
	CLPy//EG	30 m ZnCl_2_-H_2_O	180 at 0.5C	96% after 38000 cycles at 30C	/	[209]
N-Type Materials	p-PADA//EG	1 M NaPF_6_-Diglyme	294 at 50C	78.3% after 1000 cycles at 50C	/	[213]
	PZDB//PZDB	1 M LiPF_6_-EC-DEC-1:1	53 at 1C	90% after 200 cycles at 1C	127 (based on anode and cathode)	[214]
	CuTAPc// Graphite	1 M NaPF_6_-EC-DEC-1:1	196 at 0.5C	58.2% after 2500 cycles at 10C	324 (based on the anode)	[215]
	TzPz//EG	1 M LiPF_6_-EC-DEC-1:1	192 at 2C	99% after 400 cycles at 2C	150 (based on the anode)	[216]
	PAQS//EG	1 M KTFSI-DME	92 at 5C	86% after 500 cycles at 5C	/	[216]
	PAQS//NG	EMImTFSI	128.3 at 2C	/	/	[217]
	PAQS//KS6	Pyr_14_TFSI	112 at 10C	100% after 1000 cycles at 120 C	114 (based on the anode)	[217]
Carbonyl Materials	pK_2_TP//EG	1 M KPF_6_-EC-DMC-EMC-4:3:2	68 at 2C	100% after 2000 cycles at 5C	/	[221]
	Li_6_C_12_O_12_//LFP	1 M LiPF_6_-EC-DMC	80 at 1.1C	91% after 160 cycles at 1.1C	284 (based on the anode)	[222]
	PTCDA//KS6	1 M NaTFSI-Pyr_14_TFSI	153 at 2C	100% after 200 cycles at 2C	201 (based on the anode)	[223]
	PTCDA//EG	3.5 m Ca(FSI)_2_-EC-EMC-DMC	75.4 at 1C	84.77% after 350 cycles at 1C	/	[224]
	NTCDA//NG	Pyr_14_TFSI	114 at 0.1C	100% after 100 cycles at 2C	/	[225]
	PTO//KS6	Pyr_14_TFSI	165 at 0.1C	92.2% after 100 cycles at 5C	178 (based on the anode)	[226]
	PTCDI//EG	0.5 M Mg(TFSI)_2_-Pyr_14_TFSI	57.6 at 2C	95.7% after 500 cycles at 5C	/	[61]
	PTCDI//Graphite	1 m NH_4_PF_6_-NH_4_PF_6_/ADN-EMC	108 at 2C	88% after 1000 cycles at 2C	200 (based on the cathode)	[229]
	PNTO//KS6	5 M NaTFSI-EC-PC-EMC-DMC	274 at 0.2C	100% after 3200 cycles at 60C	256 (based on the anode)	[230]
	PDI-EDA//PANI	4.5 M Mg(NO_3_)_2_-H_2_O	102 at 5C	86.2% after 6000 cycles at 50C	29.1 (based on electrode materials and electrolyte)	[26]

## Novel Materials

Except for carbonaceous, metallic, and organic materials-based electrodes, several emerging materials such as metal–organic frameworks (MOFs) [232–234], covalent organic frameworks (COFs) [235, 236], and two-dimensional transition metal carbides, nitrides, or carbon-nitrides (MXenes) [237, 238] exhibit tremendous prospects for application in the field of energy storage and have attracted extensive attention in both academia and industry. Although numerous publications have been reported on the applications and achievements of these star materials in various fields, these materials are still in their infancy in electrochemical energy storage, especially in DIBs, and still face a lot of challenges to be solved urgently due to the short research time, insufficient understanding, and lack of systematic characterization and analysis as well as intrinsic mechanism exploration [239–241]. Here, we comprehensively summarize the applications of MOFs, COFs, and MXenes materials in DIBs in recent years, and systematically explore how these materials exert their unique electrochemical performance in DIBs in terms of both extrinsic synthesis and intrinsic principle.

### MOFs Materials

**Fig. 13:**
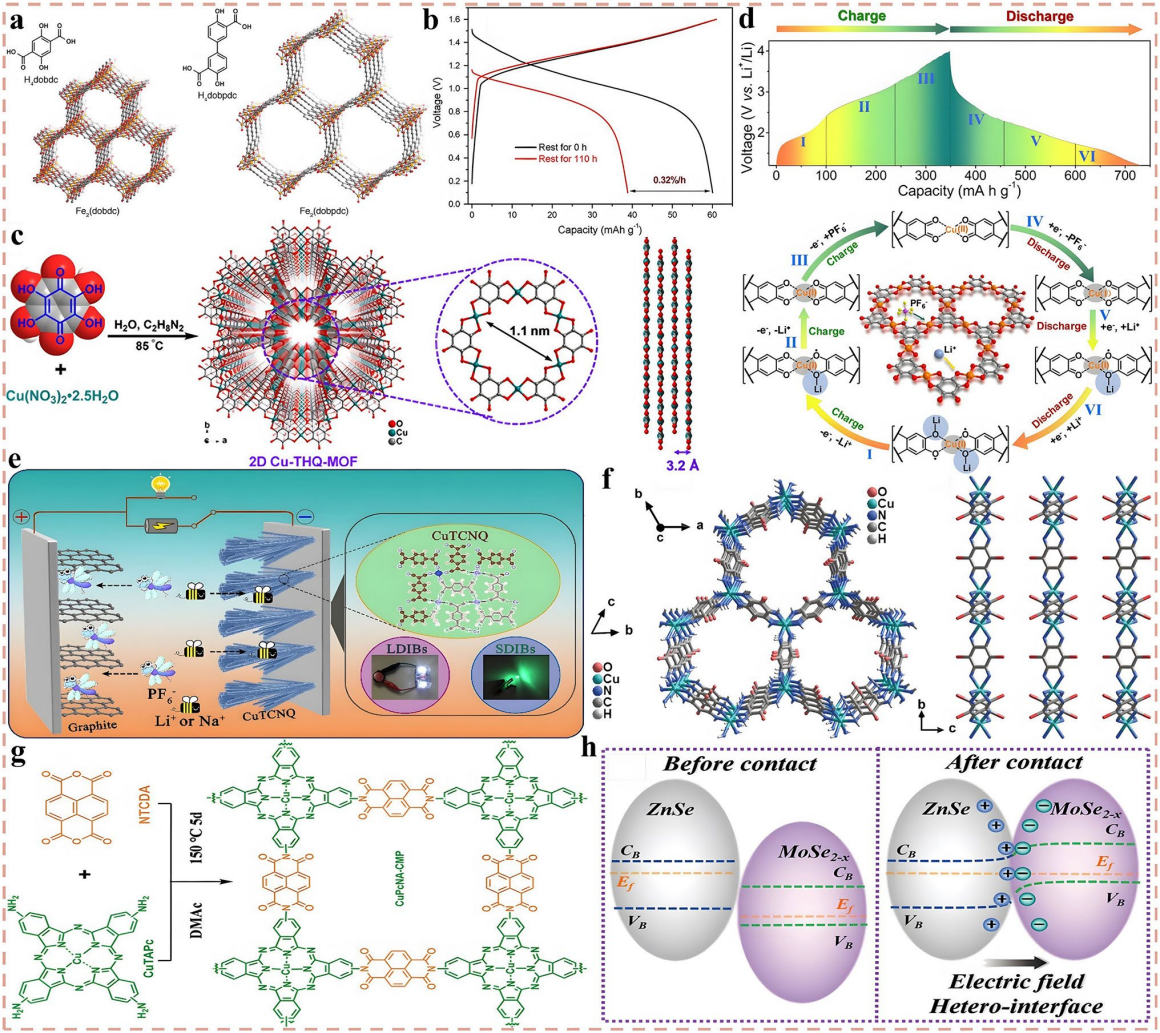
Application of MOFs-based materials in DIBs and the corresponding modification strategies. **a** Structure of Fe_2_(dobdc) and Fe_2_(dobpdc), reproduced with permission [248]. Copyright 2019, American Chemical Society. **b** Charge–discharge profile with or without rest, reproduced with permission [249]. Copyright 2018, Wiley. **c** Representation and unit-cell structure and **d** GCD curves and the evolution of electronic states of the repeating coordination unit of 2D Cu-THQ MOF, reproduced with permission [250]. Copyright 2020, Wiley. **e** Working schematic of DIBs based on CuTCNQ anode, reproduced with permission [251]. Copyright 2022, Elsevier. **f** Structures of two-dimensional layer of 2D-CuTABQ, reproduced with permission [252]. Copyright 2023, Wiley. **g** Synthesis and structure of CuPcNA-CMP, reproduced with permission [253]. Copyright 2021, Wiley. **h** Diagram of the electron transfer between ZnSe and MoSe_2-x_ contact, reproduced with permission [183]. Copyright 2023, Wiley

Metal–organic frameworks (MOFs) are a class of organic–inorganic hybrid coordination porous polymer materials with a periodic network structure formed by interconnecting inorganic metal centers (metal ions or metal clusters) with bridging organic ligands through self-assembly. Unlike either inorganic porous materials or general organic compounds, they feature both the rigidity of inorganic materials and the flexibility of organic materials [242, 243]. As a result, MOFs present huge development potential and attractive perspective in modern materials investigation, and gradually emerge as one of the most sought-after functional materials nowadays. MOFs are widely applied in electrochemical energy storage owing to their large specific surface area, low mass density, high porosity, and tunable pore size, which render them ideal for storing reactive ions [245–247]. However, there are few applications of MOFs in DIBs, and the earliest can only be traced back to 2015. Aubrey et al. synthesized two kinds of MOFs materials Fe_2_(dobdc) and Fe_2_(dobpdc) with porous topological network structure for the first time and applied in DIBs, in which dobdc^4−^ = 2,5-dioxidobenzene-1,4-dicarboxylate, dobpdc^4−^ = 4,4′-dioxidobiphenyl-3,3′-dicarboxylate [248]. Figure 13a depicts the corresponding structure of the framework, specifically, the two MOFs materials share a rigid 3D framework of hexagonal channels, with the five-coordinated Fe^2+^ ions lined up at the vertices in an infinite one-dimensional chain of common-edge square cones. The pore sizes of Fe_2_(dobdc) and Fe_2_(dobpdc) are calculated to be 12 and 21 Å, respectively, which are sufficient to accommodate the insertion/desertion of large reactive ions. In particular, both of the MOFs can store reactive ions in a reversible manner via a topotactic oxidative insertion reaction, which only causes minor lattice shrinkage without structural damage. When used in a sodium-based electrolyte system, the DIBs can release a stable SDC of 90 mAh g^−1^ at 1C with an ED as high as 316 Wh kg^−1^ (based on the mass of anode), which demonstrates a promising Na^+^ storage performance. In order to enhance the Coulombic efficiency and reduce the self-discharge rate of the MOFs-based electrode materials, a kind of MOF with nanosphere morphology-FeFe(CN)_6_ was synthesized by a hydrothermal method, which exhibits favorable electrochemical performance as an anode of DIBs [249]. When employed for the storage of Na^+^, it boasts a relatively high binding capability, which makes it difficult for Na^+^ to spontaneously detach from the crystal structure of FeFe(CN)_6_, thereby effectively reducing the self-discharge rate. The corresponding dual-ion battery system exhibits a SDC of 75.0 mAh g^−1^ and a CE higher than 99.6% at a current density of 0.2 mA cm^−2^ with a CR of 83% after 100 cycles. Furthermore, the self-discharge effect of the system is calculated through the static shelving method, as illustrated in Fig. 13b, where the battery is fully charged to 1.6 V and then subjected to static shelving, which still possesses an open-circuit voltage (OCV) of 1.21 V with a CR of 63.6% after 110 h. Therefore, the self-discharge rate is calculated to be as low as 0.32% h^−1^.

Unfortunately, MOFs usually exhibit unsatisfactory discharge capacity and cyclic stability due to their inherent low conductivity and redox activity. For this purpose, as depicted in Fig. 13c, a type of 2D Cu-THQ MOF with a 2D honeycomb lamellar framework structure has been synthesized via topological self-assembly between THQ and Cu^2+^ using ultra-small tetrahydroxy-1,4-quinone (THQ) organic linker coordinated with Cu(NO_3_)_2_ [250]. The 2D Cu-THQ MOF not only features abundant redox-active centers and nanoscale pore sizes, but also shows excellent electrochemical performance with a high conductivity of 2.15 × 10^–3^–0.16 μS cm^−1^ from 30 to 110 °C. When utilized in the lithium-based system, 2D Cu-THQ MOF underwent a 3-electron redox reaction during charging and discharging as concluded by CV test, and shares a SDC of up to 387 mAh g^−1^ as well as an ED of 775 Wh kg^−1^ (based on the mass of anode) at 50 mA g^−1^ with a CR of 85% after 100 cycles, which is one of the best performances reported in MOF-based electrode materials. The mechanism involved of Li^+^ storage is explored in depth by electron paramagnetic resonance (EPR) and X-ray absorption spectroscopy (XAS). The results are shown in Fig. 13d, where a repeating unit in the 2D Cu-THQ MOF backbone experiences a two-electron redox reaction in a voltage range of 1.2–3.2 V during the charge process, which corresponds to the conversion of C = O to C–O^−^ and coordination with 2 Li^+^ (steps I and II). After charging to 4.0 V, the third redox reaction occurs, and copper ion is oxidized from Cu^I^ to Cu^II^, accompanied by the insertion of the third Li^+^ (step III). During the subsequent discharge process, the reduction of Cu^II^ (step IV) and the reversible transformation of C–O^−^ to C=O (steps V and VI) take place sequentially, accompanied by the reversible desertion of Li^+^. However, all of the above MOFs-based DIBs display relatively low operating voltages. To solve this thorny issue, Wang et al. have synthesized a broom-like copper-tetracyanoquinodimethane (CuTCNQ) MOF with redox activity for reversible storage of Li^+^/Na^+^ [251]. When applied as anode for DIBs, as exhibited in Fig. 13e, the full batteries exhibit a high operating voltage of 4.26 V with fast reaction kinetics. The DIBs delivered a SDC of 195.4 mAh g^−1^ and more than 200 stable cycles at 0.1 A g^−1^, as well as a SDC of 106.2 mAh g^−1^ even at a high current density of 5 A g^−1^.

Interestingly, several MOFs materials can be used as both cathode and anode for storing active anions and cations, respectively, due to their redox bipolarity. A category of extensible conjugated coordination polymer (CuTABQ) synthesized by using 2,3,5,6-tetra amino benzoquinone (TABQ) ligand and Cu metal ions through π-d conjugation effect can effectively promote the long-range delocalization of electrons, and thus possesses higher conductivity and structural stability than conventional MOFs [252]. As illustrated in Fig. 13f, TABQ is arranged in a π-d conjugate stacking arrangement along the c-axis to constitute a 2D network topology with octahedral geometry, which is favorable for the storage and transport of active ions. When employed in 4 M NaPF_6_-DME-based high-concentration electrolyte system, it can act as both cathode and anode for the reversible storage of Na^+^ and PF_6_^−^ with outstanding electrochemical performance. The full batteries deliver a reversible SDC of 273.9 mAh g^−1^ and 170 mAh g^−1^ at 100 mA g^−1^ and 5 A g^−1^, respectively, and can be cycled stably for more than 2000 cycles with an average decay rate of 0.01% per cycle, implying good rate capability and cyclic stability. Moreover, a class of MOFs-CuPcNA-CMP with bipolar and bi-redox centers was synthesized via the iminization reaction of copper (II) tetraaminephthalocyanine (CuTAPc) and NTCDA (Fig. 13g), which can efficiently store Li^+^ cations and PF_6_^−^ anions based on multi-electron redox-reactions at C=O groups and Pc macrocycle [253]. DFT calculations reveal that CuPcNA-CMP possesses a lower HOMO–LUMO energy gap than the two ligands, and the introduction of Cu^2+^ results in spin polarization and a narrower bandgap, which indicates that the excitation of carrier to conduction band is enhanced and the conductivity is improved. Consequently, the organic symmetric DIBs constructed based on the CuPcNA-CMP electrode present a reversible capacity of 245.3 mAh g^−1^ at 0.2 A g^−1^ with a CR of 89% and ~ 100% CE after 500 cycles. When the rate is increased to 1 A g^−1^, the DIBs can still be cycled stably for 3000 cycles and provide a SDC of 155.3 mAh g^−1^. Remarkably, MOFs-derived electrode materials tend to exhibit more extraordinary electrochemical performance. Qian et al. prepared a class of MOFs-derived MoSe_2-x_/ZnSe@C materials with heterostructures through in situ synthesis and high-temperature selenization strategy [183]. The charge density difference (CDD) of MoSe_2-x_/ZnSe@C was calculated by DFT, and it is detected that the heterostructure between MoSe_2-x_ and ZnSe leads to the transfer of 0.62 electrons at the interface from the ZnSe to MoSe_2−x_, resulting in an abundance of holes on the ZnSe side. Meanwhile, the MoSe_2−x_ side gathers a large number of electrons, making the potential of ZnSe higher than that of MoSe_2−x_, as depicted in Fig. 13h. Therefore, a built-in electric field from ZnSe to MoSe_2−x_ is formed at the heterogeneous interface, which not only supplies additional active sites but also accelerates the ion transport. In particular, the carbon coating layer on the surface also mitigates the volume expansion induced by the insertion of reactive ions and enhances structural stability. The DIBs based on MoSe_2-x_/ZnSe@C anode, 1.0 M NaPF_6_-EC/EMC/DMC (1:1:1 v/v/v) electrolyte, and EG cathode share a SDC of 209 mAh g^−1^ and an ED of 131 Wh kg^−1^ at 0.5 A g^−1^ and can be cycled stably for 2000 cycles. Surprisingly, using ferrocene nanocomposite-modified MOF encapsulated in microporous carbon as anode, zinc-doped Prussian blue MOF-Zn_3_[Fe(CN)_6_]_2_ as cathode, and 30 m ZnCl_2_ water-in-salt electrolyte as electrolyte, a proof-of-concept reverse dual-ion battery was proposed [60]. Thanks to the ingenious and unique utilization of water-in-salt electrolyte, it can minimize the dissolution of ferrocene and effectively decrease the insertion potential of anions in anode while increase the insertion potential of cations in cathode, thus realizing the insertion of anions into anode during charging while cations are inserted into cathode. Although the exhibited electrochemical performance is moderate, achieving only a SDC of 30 mAh g^−1^ and an operating voltage of 0.9 V at 1C, with a CR of 58% after 1000 cycles. This unique design of reverse dual-ion batteries breaks the inherent perception of the working mechanism of traditional DIBs and provides decent inspiration for the development of innovative DIBs in the future.

### COFs Materials

Covalent organic frameworks (COFs) are two-dimensional or three-dimensional framework materials with regular pore channels connected by covalent bonds through organic units, which exhibit large specific surface area, designable molecular structure, easy-to-regulate framework, ordered pore structure and modifiable multi-functionality, they display considerable application potential in the fields of adsorption, separation, and catalysis [254–257]. In recent years, the research of COFs in energy storage and conversion has produced some remarkable achievements, especially in the application of electrode materials with the following advantages:

1. The diversity of construction monomers, linking groups, and synthesis methods provides many feasible strategies for the development of COFs with specific active sites and functions [258–260].
2. The large framework structure and strong covalent bonding connections of COFs ensure their stability during redox processes [261, 262].
3. The versatility of the redox-active moieties of COFs and their tunable molecular structures confer great flexibility to the charge carriers [263, 264].
4. COFs are composed of light elements such as C, H, N, and O, which is favorable to enhance the specific discharge capacity and energy density as well as reduce the cost of the battery [265, 266].

**Fig. 14:**
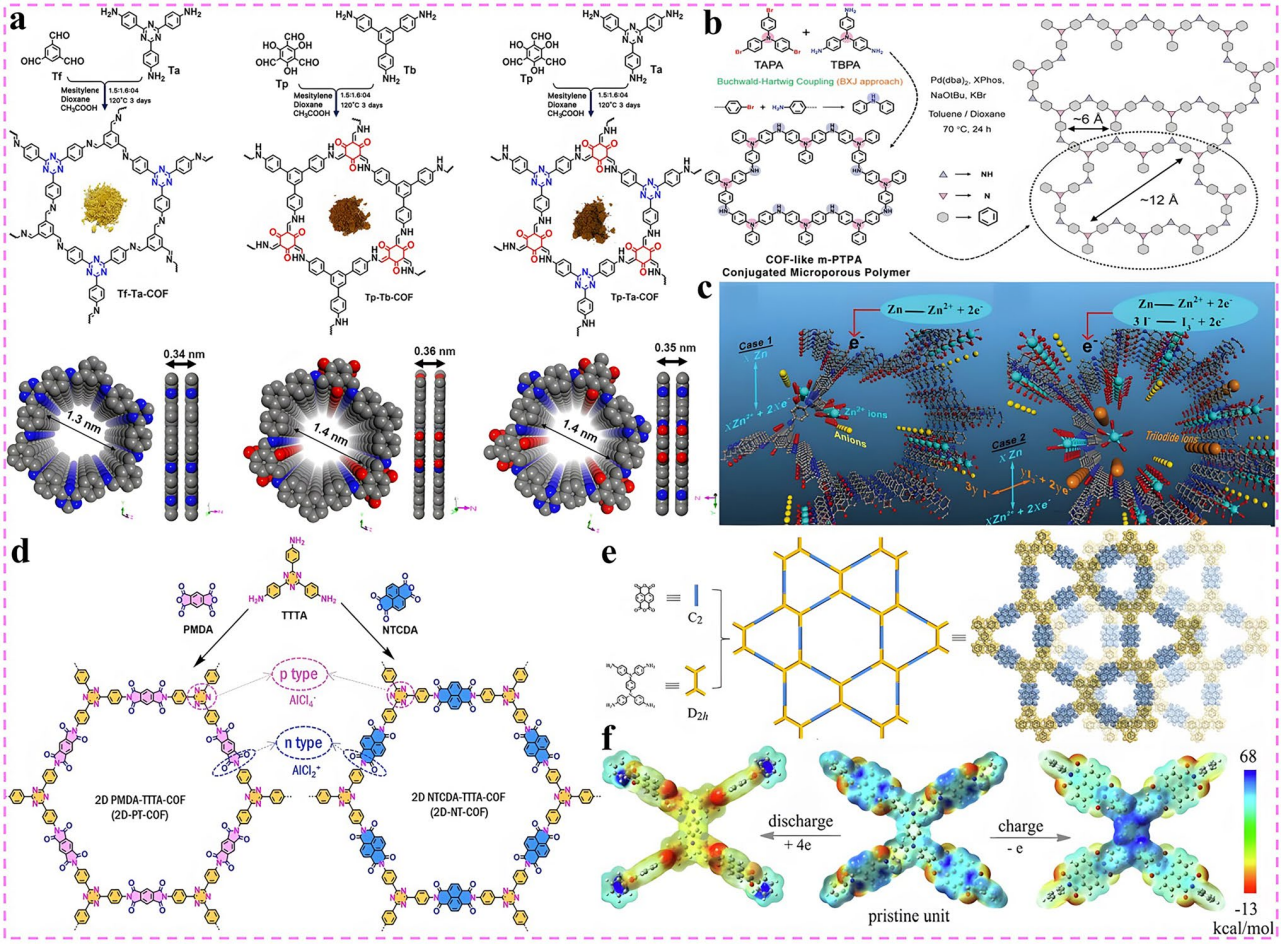
Application of COFs anode materials in DIBs and their modification strategies. **a** Synthetic route for Tf-Ta-COF, Tp-Tb-COF and Tp-Ta-COF, reproduced with permission [267]. Copyright 2022, Wiley. **b** Schematic illustration of the synthesis route of COF-like polytriphenylamine CMP and its structure, reproduced with permission [268]. Copyright 2021, Wiley. **c** Charging-discharging process of Zn-ion battery with IISERP-COF22 electrode, reproduced with permission [269]. Copyright 2023, Wiley. **d** Design and synthetic routes of redox-bipolar 2D-PT-COF and 2D-NT-COF, reproduced with permission [270]. Copyright 2023, Wiley. **e** Synthetic routes and the chemical structure of NT-PICOF and **f** molecular electrostatic potential of the pristine NT-PICOF unit (middle), n-type radical anion (left) and p-type radical cation (right), reproduced with permission [271]. Copyright 2023, Elsevier

Benefiting from the above remarkable advantages, the research of COFs as electrode materials in rechargeable batteries has received extensive attention. Guo et al. prepared a type of Tp-Ta-COF anode material with dual-redox active sites by grafting active sites C=O and C=N onto the skeleton of COF via Schiff base reaction [267]. For comparison, as illustrated in Fig. 14a, only C=O-modified COF (Tf-Ta-COF) and only C=N-modified COF (Tp-Tb-COF) were prepared. Compared with Tf-Ta-COF and Tp-Tb-COF, Tp-Ta-COF modified with double active sites possesses the highest structural stability, the smallest HOMO–LUMO energy gap and the fastest reaction kinetics, which is expected to bring the optimum electrochemical performance. It is proved by in situ FT-IR and Raman characterization that redox-active C=O and C=N are reversibly converted to C–O^−^ and C–N^−^ and bound with Li^+^ during charging and discharging. DFT theoretical calculations further demonstrate that Li^+^ preferentially reacts in the C=O active site and a single repeating unit of Tp-Ta-COF can reversibly store 18 Li^+^, which could bring about a SDC of up to 418 mAh g^−1^ and achieve 800 stable cycles. Besides, COFs can be applied to aqueous dual-ion battery systems and display satisfied electrochemical performance. Through the periodic cross-coupling reaction of tris(4-aminophenyl)amine (TAPA) and tris(4-bromophenyl)amine (TBPA) monomers, Zhang et al. successfully prepared a type of porous polytriphenylamine conjugated polymer (m-PTPA) [268]. On the one hand, m-PTPA features a specific 3D COF conjugation network and a permanent 3D microporous structure with long-range ordered extension by means of π–π conjugation, with pore diameters of mainly 6 and 12 Å (Fig. 14b). This unique porous structure enables the provision of additional active sites to activate its high ionic conductivity. On the other hand, m-PTPA displays a pseudocapacitive contribution share of nearly 100%, which is helpful to realize fast energy storage and enhance the rate capability. When employed in aqueous zinc-based dual-ion batteries, the DIBs exhibit a SDC of ~ 60 mAh g^−1^ and an ED of 236 Wh kg^−1^ (based on the mass of cathode) at 0.5 A g^−1^, but the capacity decays rapidly during the subsequent cycles. To further increase the discharge capacity and cyclic stability of COFs electrode materials in aqueous DIBs, a kind of COF (IISERP-COF22) consisting of squaric acid and chloroglucinol coupled via Schiff bonds has been reported for aqueous zinc-based DIBs [269]. IISERP-COF22 possesses a (3 + 2) multi-channel framework structure via conjugate stacking and abundant active sites to effectively chelate Zn^2+^ (Fig. 14c), which not only facilitates the transport and storage of Zn^2+^, but also effectively inhibits the volume expansion effect triggered by the insertion of Zn^2+^. When applied to the 3 M ZnSO_4_ + 0.2 M ZnI_2_-based aqueous electrolyte system, IISERP-COF22 displays decent Zn^2+^ storage performance. Here, ZnI_2_ acts as a class of redox additive to provide both additional discharge capacity and the ability to form a stable SEI layer containing polyiodide species to reduce the electrode–electrolyte interface resistance. Consequently, the flexible DIBs with high-performance based on IISERP-COF22 are constructed, which exhibit a SDC close to 600 mAh g^−1^ and are capable of stable cycling for more than 6000 cycles without excessive capacity degradation during cycling.

Similarly, it is possible to realize certain COFs with both n-type and p-type active sites through molecular engineering strategies, thus exhibiting redox bipolarity and acting as both anode for storing cations and cathode for storing anions. As presented in Fig. 14d, two 2D COFs of 2D-PT-COF and 2D-NT-COF have been synthesized by the condensation reaction between 4,4',4'-(1,3,5-triazine-2,4,6-triyl) trianiline (TTTA) monomer and two monomers of PMDA and NTCDA, respectively [270]. Both of them achieve redox bipolarity by integrating an n-type imide group and a p-type triazine group into a single backbone. When employed in aluminum-based DIBs, the electrochemical reaction process demonstrates a reversible reduction reaction of an imine molecule with an AlCl_2_^+^ cation as the charge carrier and a reversible oxidation reaction of a triazine molecule with an AlCl_4_^−^ anion as the charge carrier. Benefiting from the highly polymerized rigid framework, unique 2D pore topology, and dense redox active sites, the DIBs based on 2D-NT-COF electrodes achieve a SDC of 132 mAh g^−1^ at 100 mA g^−1^ and a long cyclic stability of 4000 cycles as well as a CE of 99.9% with a degradation rate of less than 0.0007% per cycle at 1 A g^−1^. While Gu et al. also designed a novel class of bipolar polyimide COF-NT-PICOF by employing NTCDA and N,N,N',N'-Tetra(p-aminophenyl)-p-phenylenediamine (TPPDA) monomers with both an imide active site for storing Li^+^ and a quaternary nitrogen active center for storing PF_6_^−^ (Fig. 14e) [271]. It can be observed from the molecular electrostatic potential distribution that the carbonyl portion of the imide moiety exhibits the smallest electrostatic potential value (red portion in Fig. 14f), which corresponds to the n-type active site for storing Li^+^. On the contrary, the highest electrostatic potential value is expressed around the quaternary nitrogen active group (blue portion in Fig. 14f), which corresponds to the p-type active site for storing PF_6_^−^. Besides, it is found that the utilization of electrolyte additives such as VC and FEC instead exhibits worse electrochemical performance, this is due to the fact that VC and FEC decompose on the electrode surface and form an organic layer, which increases the impedance and hinders the transport of electrons and ions. When 1 M LiPF_6_ in EC-DEC (1:1, v/v) electrolyte was adopted, the DIBs display a SDC of 165 mAh g^−1^ at 30 mA g^−1^ and stable cyclic performance for 4000 cycles at 100 mA g^−1^ with a CR of 91%. Strikingly, COFs can also be utilized as a functional electrolyte interfacial layer to enhance the electrochemical performance owing to their abundant pore space, tunable pore size, solvent resistance, and stable physicochemical properties. As Lou et al. realized the in situ growth of lithium-conducting COF on the surface of silicon nanoparticles (Si@COF) via a two-step method, as an artificial SEI layer, Si@COF exhibits excellent conductivity and superior mechanical stability [272]. Consequently, relative to the unmodified Si anode, Si@COF demonstrates higher ICE and discharge capacity, lower charge transfer impedance, and longer cycle life, which can remarkably facilitate the transport of Li^+^ and improve the cyclic stability and rate capability of the full DIBs.

### MXenes Materials

MXenes materials are a type of two-dimensional layered metal carbon/nitride with several atomic layer thicknesses, the chemical formula of which is M_n+1_X_n_T_X_, where M stands for transition metals such as Ti/Zr/V/Mo, X stands for C or N elements, and T_X_ is a surface functional group, usually -OH, -O, -F, and -Cl [273–275]. Due to the presence of hydroxyl groups or terminal oxygen on the surface of MXenes, they possess the metallic conductivity of transition metal carbides [276, 277]. Moreover, since the first reported in 2011, MXenes have been demonstrated to possess satisfactory discharge capacity, specific surface area, mechanical stability, and structural designability, making them become one of the promising anode materials in electrochemical energy storage with a wide range of applications in monovalent alkali metal ion batteries [278–281].

**Fig. 15:**
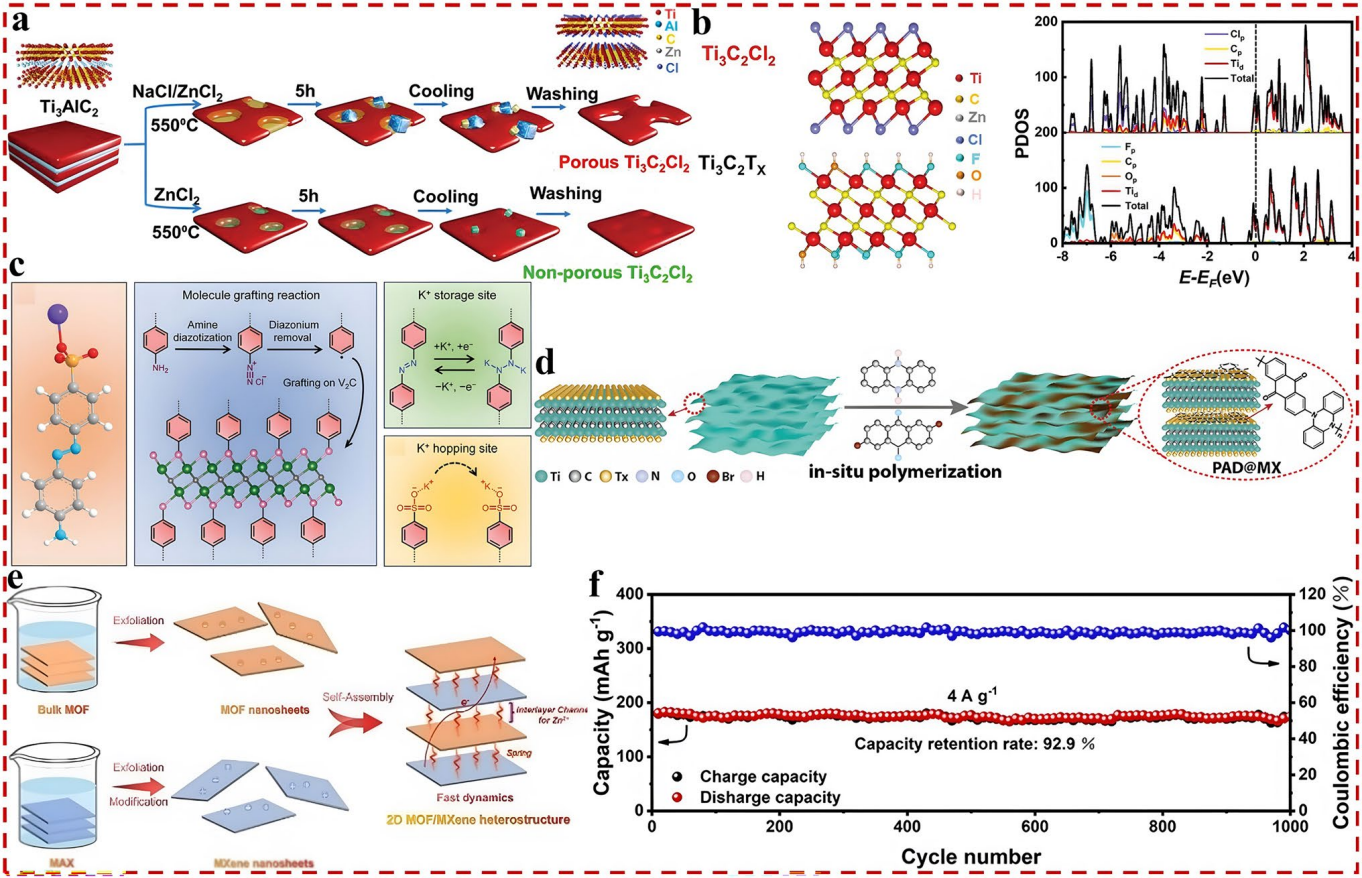
Application of MXenes anode materials in DIBs and their modification strategies. **a** Schematic illustration of in-plane porous Ti_3_C_2_Cl_2_ and **b** optimized geometries and projected density of states of Ti_3_C_2_C_l2_ and Ti_3_C_2_O_0.1_F_1.4_(OH)_0.5_, reproduced with permission [282]. Copyright 2022, Wiley. **c** Schematic illustration of 4-aminoazobenzene-4′-sulfonic acid sodium salt, molecule grafting reaction, the azobenzene unit as the extra K^+^-storage site and the sulfonate anion as the K^+^-hopping site, reproduced with permission [283]. Copyright 2024, Wiley. **d** Synthesis of PAD@MX, reproduced with permission [284]. Copyright 2024, Wiley. **e** Synthesis of Cu-HHTP/MX and **f** long-term cycling tests at 4 A g^−1^, reproduced with permission [285]. Copyright 2023, Wiley

Currently, the research on MXenes in DIBs is in its initial stage, and one of the reasons is that the synthesis condition is too harsh and accompanied by huge risk. In this regard, an eutectic mixture etching strategy for the synthesis of MXenes was proposed Chen et al. [282]. Specifically, as depicted in Fig. 15a, the phase transition of the selected salt melt is manipulated by controlling the temperature and composition of the NaCl/ZnCl_2_ mixture, thus enabling the one-step synthesis of the MAX precursor (Ti_3_AlC_2_) into a Cl-terminated MXene (Ti_3_C_2_Cl_2_) with tunable in-plane porosity, which ensures a substantial increase in the mesoporous and specific surface area of the target MXenes, and hence is expected to deliver higher theoretical capacity and faster reaction kinetics. When Ti_3_C_2_Cl_2_ was employed as anode for DIBs with the graphite cathode and 4 M LiPF_6_-EMC electrolyte, the constructed proof-of-concept full battery delivers a high SDC of 242 mAh g^−1^ at 0.1 A g^−1^ with a median discharge voltage up to 4.5 V. Even at a high rate of 1 A g^−1^, the battery achieves a SDC of 141 mAh g^−1^ and is stable for 1000 cycles with a CR of 83%. The electronic states and Li^+^ adsorption/diffusion behavior of MXenes prepared based on the above eutectic salt etching strategy and the conventional hydrofluoric acid etching method are further compared through DFT theoretical calculations. The analysis shows that due to the abundance of -Cl functional groups on the surface of Ti_3_C_2_Cl_2_, it displays a higher density of states (DOS) at the Fermi energy level in Fig. 15b, indicating better conductivity and lower Li^+^ diffusion energy barrier, which brings about superior electrochemical performance. Except for the storage of Li^+^, MXenes also exhibit decent K^+^ storage properties. For example, Feng et al. grafted multi-functional azobenzene sulfonic acid onto the surface of V_2_C MXene (ASA-V_2_C) through a molecular engineering strategy as shown in Fig. 15c [283]. Notably, ASA-V_2_C not only achieves the efficient storage of K^+^ and large layer spacing, but also provides additional active sites and fast hopping sites for K^+^, and acts as a buffer to mitigate the structural distortion during the insertion/desertion process of K^+^. When applied as the anode of DIBs, the full batteries exhibit a SDC of 51 mAh g^−1^ and an ED of 175 Wh kg^−1^ (based on the total mass of cathode and anode) at 0.1 A g^−1^, with a CR of 86.9% after 500 cycles.

Similarly, as illustrated in Fig. 15d, a bipolar MXene with redox activity was obtained via in situ synthesis of the two-electron n-type repeating unit dibromoanthraquinone and p-type repeating unit dihydrophenazine on lamellar MXene nanosheets (PAD@MX) [284]. In which the dibromoanthraquinone moiety can be employed for storing cations and the dihydrophenazine moiety can be utilized for storing anions. Furthermore, the wide interlayer channels, high conductivity, and large specific surface area of MXene is quite favorable for the storage and the transport of reactive ions and electrons. The designed bipolar MXene was applied for both sodium and potassium-based systems, and the corresponding DIBs deliver SDCs of 104 and 86 mAh g^−1^ as well as EDs of 64 and 54 Wh kg^−1^ (based on the total mass of cathode and anode), respectively, at 200 mA g^−1^, and are able to cycle stably for 500 cycles. With the aim of further improving the discharge capacity and cycle stability of MXenes-based electrode materials in a multi-valent metal-ion battery system, a kind of 2D sandwich-like MOF/MXenes heterostructure composite (Cu-HHTP/MX) was designed by self-assembling with alternating stacks of MOF and MXenes layers [285]. Cu-HHTP/MX presents large ion channels, abundant active sites, improved conductivity, and excellent structural stability. Theoretical calculations indicate that this unique heterogeneous structure significantly reduces the adsorption energy and diffusion energy barriers to Zn^2+^, and electrochemical measurements also confirm the ideal pseudocapacitive behavior of Cu-HHTP/MX, which enhances the kinetics of electrochemical reactions and rate capability. When applied to the Zn^2+^-based system, the full batteries exhibit an ultra-high SDC of 260.1 mAh g^−1^ and an ED of 200.96 Wh kg^−1^ (based on the mass of anode) at 0.1 A g^−1^. Even at a high current density of 4 A g^−1^, the batteries still achieve a SDC of 166.9 mAh g^−1^ with a CR of 92.9% after 1000 cycles (Fig. 15f), demonstrating the excellent electrochemical performance.

Overall, although the above-mentioned emerging star materials are still in their infancy for the application in DIBs, they are increasingly becoming the most promising anode materials due to the excellent electrochemical performance they exhibit. However, the ICE is low and more electrolyte dose is required due to their large active specific surface area, and their intrinsic reaction mechanism and structural evolution have not been fully explored. In the future, on the one hand, active ion compensation such as pre-lithiation could be adopted to increase ICE and reduce the dose of electrolyte. On the other hand, it is necessary to conduct a clearer and more thorough exploration of the energy storage mechanism as well as structural evolution of these materials during charging and discharging processes. Besides, for the convenience of comparison and better understanding by researchers, Table 4 details the configurations and electrochemical performance of DIBs based on various emerging anode materials.

**Table 4:** Configurations and electrochemical performance of DIBs based on various emerging anode materials

Type	Anode//Cathode Configuration	Electrolyte systems	SDC (mAh g^−1^)	Cyclic performance	Energy density (Wh kg^−1^)	Refs.
MOFs	Fe_2_(dobdc)// Graphite	0.6 M NaPF_6_-EC-DMC-3:7	90 at 1C	100% after 50cycles at 1C	316 (based on the anode)	[248]
	FeFe(CN)_6_// NG	1 M NaTFSI-EMImTFSI	75 at 0.2C	83% after 100 cycles at 0.2C	/	[249]
	Cu-THQ//EG	1 M LiPF_6_-EC-DEC-1:1	387 at 0.5C	78% after 1000 cycles at 50C	775 (based on the anode)	[250]
	CuTCNQ// Graphite	4 M LiPF_6_-EMC	80.9 at 1C	67% after 100 cycles at 1C	/	[251]
	CuTABQ//EG	4 M NaPF_6_-DME	273.9 at 1C	80% after 2000 cycles at 50C	/	[252]
	CuPcNA// CuPcNA	4 M LiPF_6_-EMC	245.3 at 2C	89% after 500 cycles at 2C	/	[253]
	MoSe_2-x_/ ZnSe@C//EG	1 M NaPF_6_-EC-EMC-DMC-1:1:1	209 at 0.5C	53% after 1000 cycles at 10C	131 (based on anode and cathode)	[183]
	Fc/C//Zn_3_Fe(CN)_6_	30 m ZnCl_2_-H_2_O	30 at 1C	58% after 1000 cycles at 1C	/	[60]
COFs	m-PTPA//EG	2 M ZnCl_2_-H_2_O	60 at 5C	88% after 1000 cycles at 5C	236 (based on the cathode)	[268]
	IISERP-COF22//NG	3 M ZnSO_4_ + 0.2 M ZnI_2_	600 at 1C	67% after 6000 cycles at 50C	/	[269]
	2D-NT-COF//EG	EMImCl	132 at 1C	97% after 4000 cycles at 10C	/	[270]
	NT-PICOF// Graphite	1 M LiPF_6_-EC-DEC-1:1	165 at 0.3C	91% after 4000 cycles at 1C	/	[271]
MXene	Ti_3_C_2_Cl_2_// Graphite	4 M LiPF_6_-EMC	242 at 1C	83% after 1000 cycles at 10C	/	[282]
	ASA-V_2_C// Graphite power	5 M KFSI-DMC-EC-1:1	51 at 1C	87% after 500 cycles at 1C	175 (based on anode and cathode)	[283]
	PAD@MX// PAD@MX	2.2 M KPF_6_-DEGDME	104 at 2C	74% after 100 cycles at 2C	54 (based on anode and cathode)	[284]
	CuHHTP-MX//EG	2 M Zn(TfO)_2_ -H_2_O	260.1 at 1C	93% after 1000 cycles at 40C	200.96 (based on the anode)	[285]

## Summary and Perspectives

**Fig. 16:**
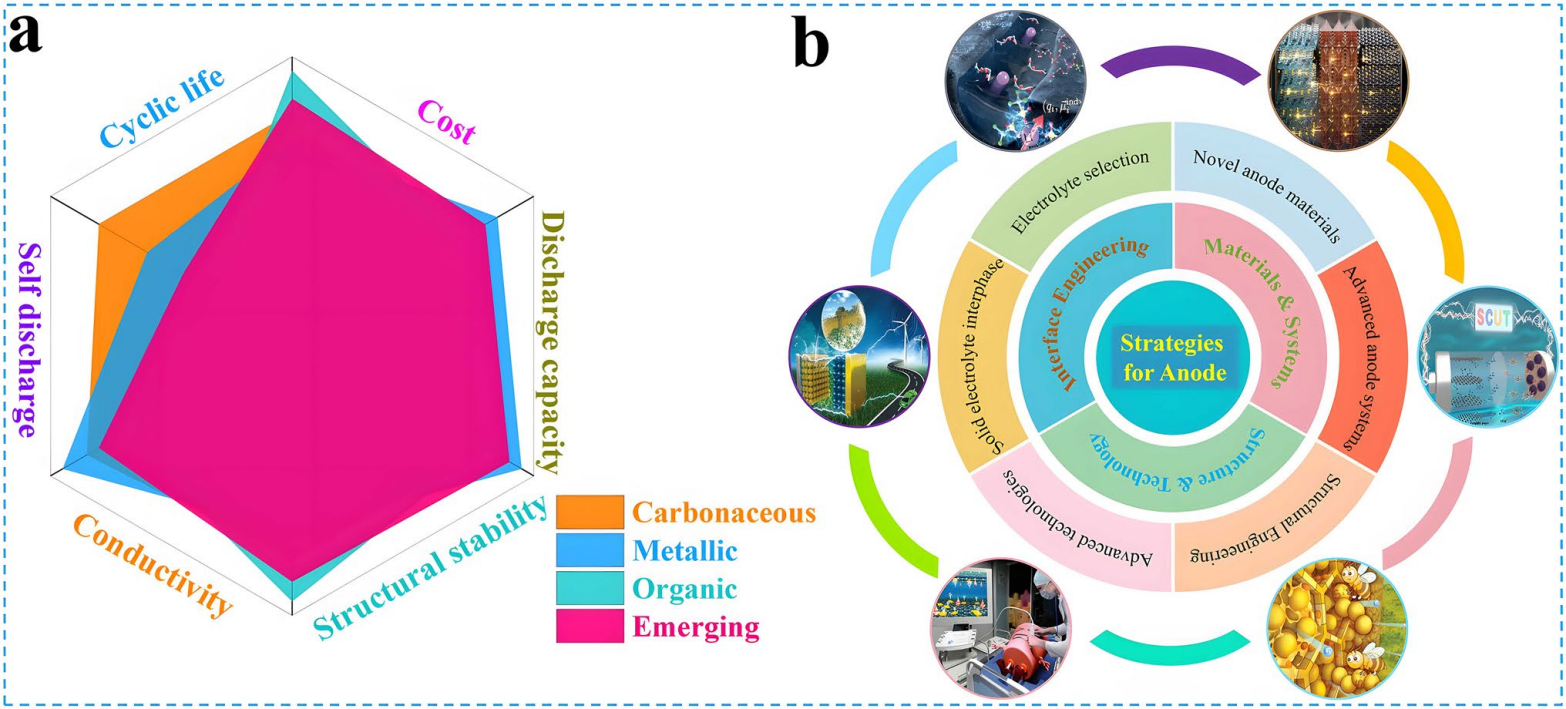
**a** Comparison of the overall performance of the four anode materials. **b** Summary of prospective strategies for DIBs anode materials

Sustainable novel energy and the "peak carbon emission and carbon neutrality" strategy are the themes of today's development, and efficient and sustainable energy storage and conversion technologies are considered the most promising solutions to achieve this goal. Owing to the high operating voltage, low cost and environmental friendliness, dual-ion batteries have attracted more and more attention from researchers as prospective electrochemical energy storage devices in the future [285–287]. In this review, we first summarize the historical development process, structural features and working mechanisms of DIBs. Especially, a comprehensive and exhaustive summary of the categories, morphologies, structures, properties, and reaction mechanisms of DIBs anode materials as well as their applications in Li/Na/K/Mg/Zn/Ca/Fe/Al/Sn/Ge/Se/Si-based DIBs systems are presented. According to the physicochemical properties of the materials, the anode materials for DIBs are mainly categorized into four groups as carbonaceous, metallic, organic, and emerging materials. Although the above-mentioned anode materials achieved some encouraging results after continuous exploration, modification, and optimization in recent years, unlike the mature technology of LIBs and their enormous success in the energy storage market, the development of DIBs is still in its initial stage and facing a lot of challenges in the practical application. As presented in Table 5, different electrode materials exhibit their own strengths and weaknesses. Moreover, Fig. 16a further compares the overall performance of these four materials in terms of cyclic life, cost, discharge capacity, structural stability, conductivity and self-discharge. Specifically, carbonaceous electrode materials typically present high working voltage, low cost, and excellent active ion storage capability, but with limited active sites and low theoretical capacity. Metallic materials are distinguished for their ultra-high theoretical capacity and outstanding conductivity, but the high cost and electrochemical degradation caused by large volume expansion limits their further application. Organic materials are well-known for their wide sources, environmental friendliness and structural designability, but they possess poor conductivity, high operating potential and severe solubility in non-proton electrolytes. Emerging materials exhibit excellent structural stability and satisfactory operating voltages as well as theoretical capacities and have gradually become a research hotspot in recent years. However, their intrinsic reaction mechanisms and storage principles have not been fully explained so far, and there is a lack of suitable characterization techniques. Therefore, it is necessary to explore and develop outstanding anode materials, novel and ingenious synthesis strategies, as well as innovative characterization and analysis technologies to overcome the above constraints and challenges and improve the core competitiveness of DIBs in energy storage, thus achieve large-scale practical applications at an early date. Here, through the schematic in Fig. 16, we concisely outline a set of promising strategies as effective guidance for the next generation of anode materials and thus add wings the advanced DIBs.

**Table 5:** The advantages and disadvantages of different anode materials

Anode materials	Advantages	Disadvantages
Carbonaceous materials	Low cost, easy to prepare	High self-discharge rate
	Environment-friendly	Tend to collapse and spall
	Low insertion potential	Strict electrolyte requirements
Metallic materials	High theoretical capacity	Large volume expansion
	Low self-discharge rate	Prone to metal dendrites
	Favorable conductivity and kinetics	Poor cyclic life
Organic materials	Wide sources	Difficulty in synthesis
	Structural designability	Severe dissolution in electrolyte
	Decent rate capability	High operating potential
Emerging materials	High active specific surface area	Unclear working mechanism
	Large and tunable pore size	Difficult to characterize
	Stable cycling performance	Poor ICE, high electrolyte usage

### Micro-/Nanostructure Design and Morphology Modulation

As mentioned above, many synthetic methods and engineering strategies have been extensively investigated in practice and proved to deliver great improvements and enhancements to the performance of the target products. Among them, the micro-/nanostructure engineering design strategy by constructing specific and controllable morphology is considered to be the most feasible and effective optimization scheme of electrode active materials for energy storage and conversion [288–292]. It has been widely applied to almost all electrochemical energy storage systems, such as alkali metal-ion batteries, and significantly improves the comprehensive performance of the devices. On the one hand, micro-/nanostructure design enhances the compatibility of electrode with electrolyte and is more conducive for electrolyte penetration, which plays a crucial role in increasing the active specific surface area, expanding the number of active sites, facilitating the transport of electrons/ions, and improving the reaction kinetics, which is expected to bring about higher discharge capacity and rate capability. On the other hand, the modulation of specific morphology not only improves the structural stability and suppresses the volume expansion effect, but also exposes more defective active sites for storing reactive ions, which is conducive to delivering superior cyclic stability. However, this strategy also faces the fact that a high specific surface area will consume a large amount of electrolyte on the surface of the anode for the formation of SEI, which will inevitably result in more irreversible capacity loss during the initial cycles, and consequently reduce the ICE. A trade-off needs to be made in the future.

### Electrode/Electrolyte Interface Engineering

The design and optimization of electrode/electrolyte interface is necessary for achieving high-performance DIBs, and this is especially important for the anode side [293–297]. The implication of interfacial modification is to ensure the formation of a superior performing SEI layer, which should possess high mechanical properties and cationic conductivity, and can be stable while achieving rapid cation transport without further consuming electrolyte for replenishment, and especially be able to inhibit the oxidative decomposition of electrolyte at a high potential. Besides, it also offers a protective effect on the anode, being able to selectively allow the reversible insertion of cations but avoiding the co-intercalation of solvent molecules and inhibiting the formation of dendrites as well as the expansion of the anode, which in turn improves the electrochemical performance of DIBs. Currently, high-voltage electrolyte is crucial to improve the electrochemical performance of DIBs, and the high-concentration electrolyte can enable the preferential decomposition of anions (PF_6_^−^, FSI^−^, TFSI^−^) to form SEI enriched with inorganic components such as LiF, which can dramatically enhance the charge–discharge efficiency and cyclic stability of DIBs. However, the employment of concentrated electrolytes not only requires more electrolyte dosage, which inevitably increases the cost and causes serious corrosion of the current collector, resulting in the degradation of cycle life, but also increases the viscosity, reducing the transport kinetics of cations and rate capability of DIBs. To avoid these issues, electrolyte systems with more superior performance need to be developed to match the electrode materials of DIBs. For example, introducing functional electrolyte additives or constructing an artificial electrode/electrolyte interface to form a more stable SEI and enhance the performance of batteries. Furthermore, some innovative electrolyte systems such as phosphorus-based and solid-state electrolytes, not only exhibit decent flame retardancy, but also effectively avoid the generation of dendrites and boost the ionic conductivity. However, the understanding of composition, formation and evolution mechanisms of the electrode/electrolyte interface is still quite limited. In this regard, more in-depth experimental analyses and theoretical calculations should be required for the construction of an ideal electrode/electrolyte interface. Besides, as electrolyte is the single source of active ions and its concentration would be changed with the shift of active ions, which will also affect the electrochemical performance of DIBs to a certain extent. Therefore, the effect of variation in active electrolyte concentration on the stability of electrolyte/electrode interface needs to be further investigated in the future.

### Innovative Anode Materials and Systems

To meet the requirements of sustainable development strategies and electrochemical energy storage devices, the exploitation of innovative and efficient anode material is undoubtedly one of the most important research directions in the future [298–301]. For the reasonable design and modification of anode materials, the following principles should be followed. Advanced anode materials should be characterized by high theoretical capacity and low operating potential to essentially achieve the high specific discharge capacity, remarkable working voltage and energy density of DIBs. More importantly, the anode materials should have efficient cation reduction reaction and rapid transport kinetics to match the fast insertion kinetics of anions on the cathode side and achieve superior rate capability; high binding capability to avoid spontaneous release of active ions, thereby reducing self-discharge rate; favorable compatibility with the electrolyte to form a solid electrolyte interphase (SEI) with good mechanical strength and ion transport, and to realize the stabilization and concentration requirements of the electrolyte under high voltage. Especially, based on the physicochemical properties of the materials themselves, different types of anode materials have their own corresponding optimization strategies. For carbonaceous anode materials, which usually exhibit low theoretical capacity and structural stability, it is possible to try to improve the electrochemical performance from the perspective of developing novel materials such as carbon-based materials with large layer spacing, functional surface-active groups, heterogeneous elemental doping, high degree of disorder and defects, and low interlayer van der Waals force, or carbon-quantum dots. For metallic materials, future attempts can be made to directly adopt alkali metals (lithium/sodium/potassium) as freestanding anode, which is expected to bring higher theoretical capacity and energy density. However, due to the severe volume expansion effect and the formation of metal dendrites, modifications such as the construction of artificial SEI, composite with other materials or the design of 3D structures with unique morphology are essential. Moreover, the development of mixed phase heterostructure metals or alloys with binary or more phases can also improve the electrochemical performance to an extent. For organic anode materials, the synthesis of electrode materials with high capacity, outstanding stability, superior conductivity, and low solubility by using molecular engineering strategies is one of the current research priorities. Besides, the construction of all-organic dual-ion batteries is also expected to greatly reduce the manufacturing cost and considerably enhance the environmental efficiency, which promises to achieve large-scale green and sustainable electrochemical energy storage applications.

### Advanced Characterization Techniques

Generally speaking, the systematic investigation of DIBs is still in the early stage with many technical challenges, and the intrinsic storage mechanism and structural evolution of most electrode materials are explored by some ex situ characterization methods, which are undoubtedly subject to huge errors, and possess serendipity and misdirection. Therefore, it is necessary to combine advanced characterization techniques, DFT and computational simulation to address various scientific and technical challenges, and to deeply investigate the reaction processes, the evolution mechanism, and the decay principle under practical operating conditions, which is of great significance to guide and improve the design of DIBs [302–305]. Furthermore, there is little research reported about the safety of DIBs, and the relevant safety performance tests such as combustion, short circuit, puncture, compression, and gas production need to be conducted for DIBs in the future. In summary, DIBs exhibit attractive performance that is comparable to commercial LIBs and are promising for application as the next generation of rechargeable batteries, but there are still many scientific and technical issues that need more effort to be perfected.
